# Circular RNA *circNrip1* Interacts with SYNCRIP to Promote Neuropathic Pain by Stabilizing *Tlr2* mRNA in Primary Sensory Neurons

**DOI:** 10.1002/advs.202519740

**Published:** 2026-04-09

**Authors:** Xiaozhou Feng, Jifang Xiao, Congcong Huang, Bao‐Chun Jiang, Bing Wang, Xu Li, Shibin Du, Tolga Berkman, Chun‐Hsien Wen, Weihua Cai, Dilip Sharma, Kun Wang, Longfei Ma, Huijuan Hu, Yuan‐Xiang Tao

**Affiliations:** ^1^ Department of Anesthesiology New Jersey Medical School Rutgers The State University of New Jersey Newark USA; ^2^ Department of Physiology Pharmacology & Neuroscience New Jersey Medical School, Rutgers The State University of New Jersey Newark USA; ^3^ Department of Cell Biology & Molecular Medicine New Jersey Medical School, Rutgers The State University of New Jersey Newark USA

**Keywords:** *circNrip1*, dorsal root ganglion, FUS, neuropathic pain, peripheral nerve injury, SYNCRIP, toll‐like receptor 2

## Abstract

Nerve injury–induced gene dysregulation in the dorsal root ganglion (DRG) is considered a key molecular basis for neuropathic pain genesis. Circular RNA is emerging as a critical regulator of gene expression. Here, we reported a novel circular RNA *circNrip1* formed by back‐splicing from exon 3 to exon 2 of the *Nrip1* pre‐RNA. Peripheral nerve injury upregulates *circNrip1*, but not *Nrip1* mRNA, in injured DRG neurons, at least in part due to increased binding of the RNA‐binding protein FUS to *Nrip1* pre‐RNA, thereby promoting *circNrip1* formation. Blocking this upregulation attenuates nerve injury–induced increases in toll‐like receptor 2 (*Tlr2*) mRNA and TLR2 protein levels in injured DRG, as well as nerve injury–induced nociceptive hypersensitivity. Conversely, mimicking this upregulation elevates DRG *Tlr2* mRNA and TLR2 protein expression and produces neuropathic pain‐like symptoms in naïve mice. Mechanistically, upregulated *circNrip1* enhances its binding to the 3′‐ untranslated region (UTR) of *Tlr2* mRNA and to the RNA‐binding protein SYNCRIP, thereby recruiting more SYNCRIP to the *Tlr2* mRNA 3′‐UTR and stabilizing *Tlr2* mRNA in injured DRG neurons. Thus, *circNrip1* contributes to neuropathic pain by promoting SYNCRIP‐triggered stabilization of TLR2 in DRG neurons and represents a promising therapeutic target for intervention.

## Graphical abstract

Model of *circNrip1* (*cNrip1*) upregulation driving neuropathic pain mechanisms. After peripheral nerve injury, increased FUS triggers the formation and upregulation of *cNrip1* in injured DRG neurons. Upregulated *cNrip1* recruits SYNCRIP to the 3′‐UTR of *Tlr2* mRNA by binding to both, thereby promoting SYNCRIP‐triggered *Tlr2* mRNA stability and increasing TLR2 expression on the neuronal membrane.

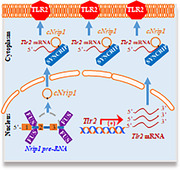

## 1 Introduction

Neuropathic pain is a chronic disorder that arises from damage to, or dysfunction of, the somatosensory nervous system. It is a major public health problem with an incidence of approximately 6.9%–10% of the world population [1]. In the United States, total estimated annual cost of neuropathic pain—including healthcare expenses and productivity losses—reaches approximately $660 billion [1]. Current therapeutic management of neuropathic pain remains largely ineffective, as commonly used medications—including both opioids and non‐opioid drugs—often fail to provide sufficient relief and/or are associated with significant side effects in the majority of patients [2, 3]. Thus, there is an urgent need to identify new targets and mechanisms underlying neuropathic pain, which may pave the way for the development of novel treatments.

Toll‐Like Receptor 2 (TLR2), a member of the TLR family, is expressed on the cell membrane for the recognition of pathogen‐ or damage‐associated molecules and has been implicated in neuropathic pain. TLR2 is expressed in neurons of dorsal root ganglion (DRG), particularly in small and medium primary sensory neurons [4]. The levels of *Tlr2* mRNA and TLR2 protein are time‐dependently increased in injured DRG following peripheral nerve injury [5]. Perineural administration of the TLR2 antagonist ameliorates type 2 diabetes mellitus‐associated neuropathic pain [6], whereas the endogenous agonist of TLR2 induces neuropathic pain‐like symptoms [7]. Moreover, TLR2 knockout mice display impaired inflammation‐ or nerve injury‐induced nociceptive hypersensitivity [4, 8]. Thus, TLR2 increase in injured DRG neurons is likely essential for neuropathic pain genesis. However, the mechanisms underlying this increase remain poorly understood. Elucidating how nerve injury induces TLR2 expression in the DRG may uncover novel therapeutic targets for the treatment of neuropathic pain.

Circular RNAs (circRNAs) are a recently identified class of endogenous noncoding RNAs characterized by covalently closed continuous loops formed through non‐canonical back‐splicing [9]. Due to the absence of the 5’ and 3’ ends, circRNAs are resistant to exonuclease degradation and thereby being more stable than linear RNAs [9]. They are evolutionarily conserved across multiple species and expressed in a tissue‐, cell‐ and development‐specific patterns [10, 11]. circRNAs have been demonstrated to participate in multiple biological processes [12, 13] and disease progressions [14, 15, 16] including neuropathic pain [17, 18] through sponging microRNAs, interacting with proteins, regulating transcription, influencing mRNA splicing, or serving as the template for protein translation, *etc* [19, 20, 21]. Several circRNAs (e.g., circAnks1a and circRNA‐Filip1l) in spinal cord have been shown to contribute to neuropathic pain via sponging microRNAs [17, 18]. However, the role of DRG circRNAs in neuropathic pain remains unclear, as previous studies have primarily used intrathecal delivery for circRNA knockdown, a method that affects both the DRG and spinal cord [22]. Moreover, it remains unclear whether circRNAs contribute to neuropathic pain through mechanisms independent of microRNA sponging.

In this study, we identify a unique and new circRNA and named *circNrip1* (*cNrip1*), because it is generated by back splicing from exon 3 to exon 2 of *Nrip1* precursor RNA (pre‐RNA). *cNrip1* (not *Nrip1* mRNA) is upregulated in neurons of injured DRG, but not spinal cord, following peripheral nerve injury. This upregulation is required for the development and maintenance of nerve injury‐induced nociceptive hypersensitivity by promoting SYNCRIP (synaptotagmin‐binding cytoplasmic RNA‐interacting protein)‐triggered stabilization of TLR2 in DRG neurons. *cNrip1* in the DRG likely acts as an adaptor or recruiter, contributing significantly to the pathogenesis of neuropathic pain.

## 2 Results

### 2.1 Identification of *cNrip*1 and Its Distribution in DRG

**FIGURE 1:**
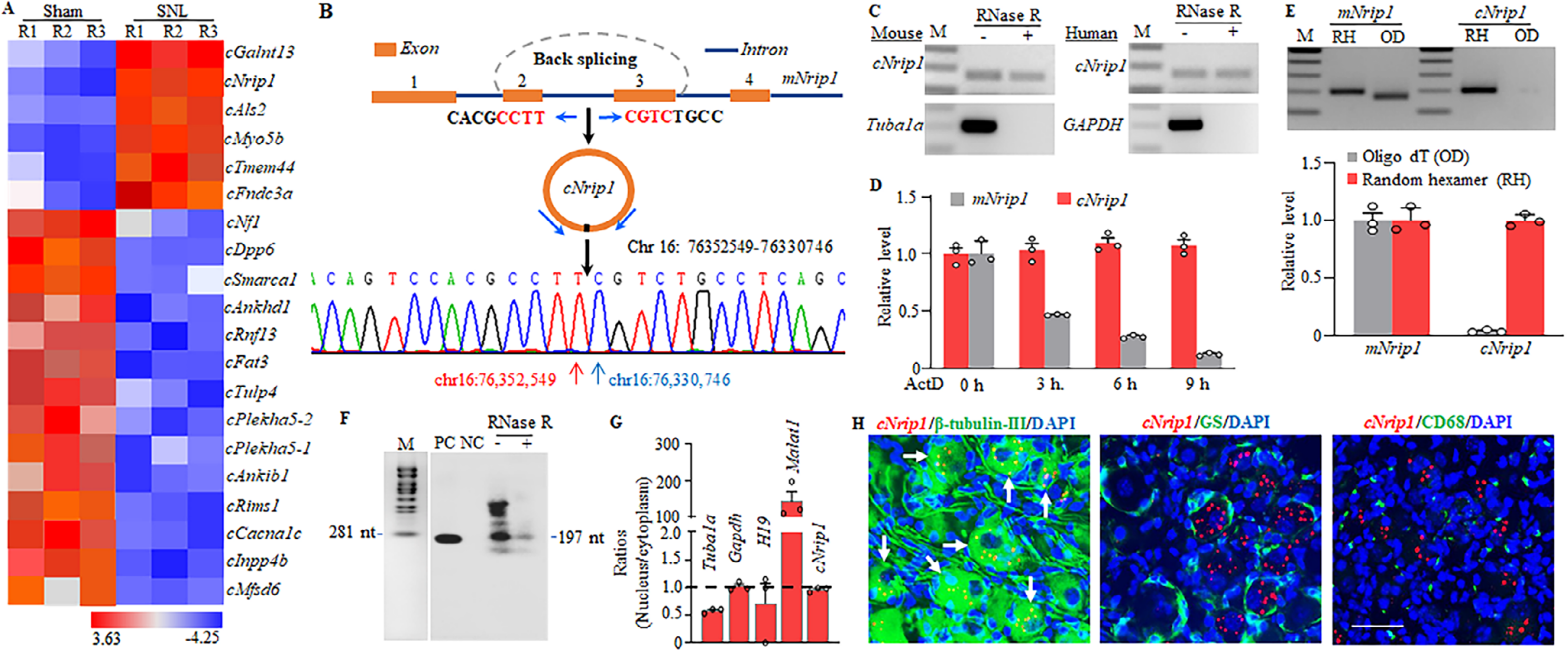
Identification of *cNrip1* in the DRG. (A) Heatmaps showing differentially expressed circRNAs (≥ twofold change) in the ipsilateral L4 DRG of male mice on day 7 following SNL or sham surgery. *n* = 3 repeats (R1‐R3)/group. The scaled heat maps were created using ZA‐score values obtained from RNA sequencing. Red color: high. Blue color: low. (B) Schematic diagram showing the genomic loci of *circNrip1* (*cNrip1*). The junction of *cNrip1* was amplified using back‐to‐back primers and then sequenced by Sanger sequencing. Arrows indicate divergent primers binding to the genome region of *cNrip1*. *mNrip1: Nrip1* mRNA. (C) *cNrip1* expression in the lumbar DRGs from naive mouse and human treated with or without RNase R using RT‐PCR assay with strand‐specific primers. Linear *Tuba1a* and *GAPDH* mRNAs were used as negative controls. M: DNA ladder marker. *n* = 3 repeats/species. (D) Levels of *cNrip1* and *mNrip1* in the cultured DRG neurons at different hours (h) after treatment with actinomycin D (ActD). *n* = 3 repeats/time point. (E) Levels of *cNrip1* and *mNrip1* in cultured DRG neurons were analyzed by quantitative PCR following reverse transcription (RT) using either random hexamer primers (RH) or oligo (dT) primers (OD). *n* = 3 repeats/RT. (F) Northern blotting assay showing *cNrip1* expression in mouse DRGs. The extracted RNA from the DRG was treated with or without RNase R. PC: positive control, in which the plasmid expressed full‐length *cNrip1*. NC: negative control, in which sense probe was used. M: RNA marker. *n* = 3 mice. (G) Distribution of *cNrip1, Tuba1a, Gapdh, H19* and *Malat1* mRNAs in the nucleus and cytoplasm of the cultured DRG neurons from mice*. n* = 3 mice. (H) Representative photomicrographs showing that *cNrip1* (red) was co‐expressed with β‐tubulin III (green, a neuronal marker) in individual DRG cells (arrows; signal particles ≥ 3) and undetected in glutamine synthetase (GS, green, a marker of satellite cell)‐ or CD68 (green, a marker of microphage and monocytes)‐labeled DRG cells in the ipsilateral L4 DRG 7 days after SNL. DAPI: a marker of cellular nucleus. Scale bar: 40 µm.

To identify the role of DRG circRNAs in neuropathic pain, we deeply analyzed our previous circRNA expression profiles from the ipsilateral L4 DRG on day 7 post‐SNL or sham surgery in male mice [23]. Approximately 6 upregulated and 14 downregulated circRNAs with ≥ 1.5‐fold changes were found from 1,252 candidates (Figure 1A). *cNrip1* was one of them that showed the most significant increases in injured DRG (Figure 1A). Our further analysis revealed that *cNrip1* was located on chromatin 16 (76 352 549–76 330 746) and derived from the downstream exon 3 to the upstream exon 2 of the *Nrip1* gene (197 nt; Figure 1B). Sanger sequencing verified this back‐splicing junction (Figure 1B). To validate circular characterization of *cNrip1*, we tested its stability. *cNrip1* from the DRG of mice or human was resistant to RNase R or exonuclease digestion, while linear *Tuba1a* mRNA and *GAPDH* mRNA were easily digested (Figure 1C; Figure S1). *cNrip1* also showed a much slower degradation and longer half‐life than *Nrip1* mRNA (Figure 1D). Unlike *Nrip1* mRNA, the expression of *cNrip1* was difficult to detect when the random hexamer primers were replaced by oligo dT primers (Figure 1E). The results further confirm that *cNrip1* is circular in form.

We also conducted northern blotting assay and confirmed the existence of *cNrip1* at the expected size in mouse DRG that was subjected to RNase R treatment (Figure 1F). Besides DRG, it was also detected in other body tissues (e.g., trigeminal ganglion and spinal cord) of naïve mice (Figure S2). Quantitative analysis of nuclear/cytoplasmic RNA extracted from DRG revealed that *cNrip1* was more enriched in the cytoplasm (Figure 1G). Cellular distribution pattern of *cNrip1* was also examined using BaseScope in situ hybridization assay, followed by immunohistochemistry assay of DRG cell‐specific markers. *cNrip1*‐labeled signal particles were undetected or sparsely detected in naïve or sham DRG, but densely detected in the SNL DRG (Figure 1H). The labeled particles (≥ 3 particles) were more enriched in the cytoplasm than in the nucleus of β‐tubulin III (a specific neuronal marker)‐positive cells (Figure 1H). These particles were undetected in glutamine synthetase (a marker for satellite glial cells)‐ or CD68 (a maker for macrophages and monocytes)‐positive cells in the ipsilateral L4 DRG on day 7 post‐SNL (Figure 1H). The evidence indicates neuronal expression of *cNrip1* in injured DRG.

### 2.2 Peripheral Nerve Injury Upregulates *cNrip1* Expression in Injured DRG Neurons

**FIGURE 2:**
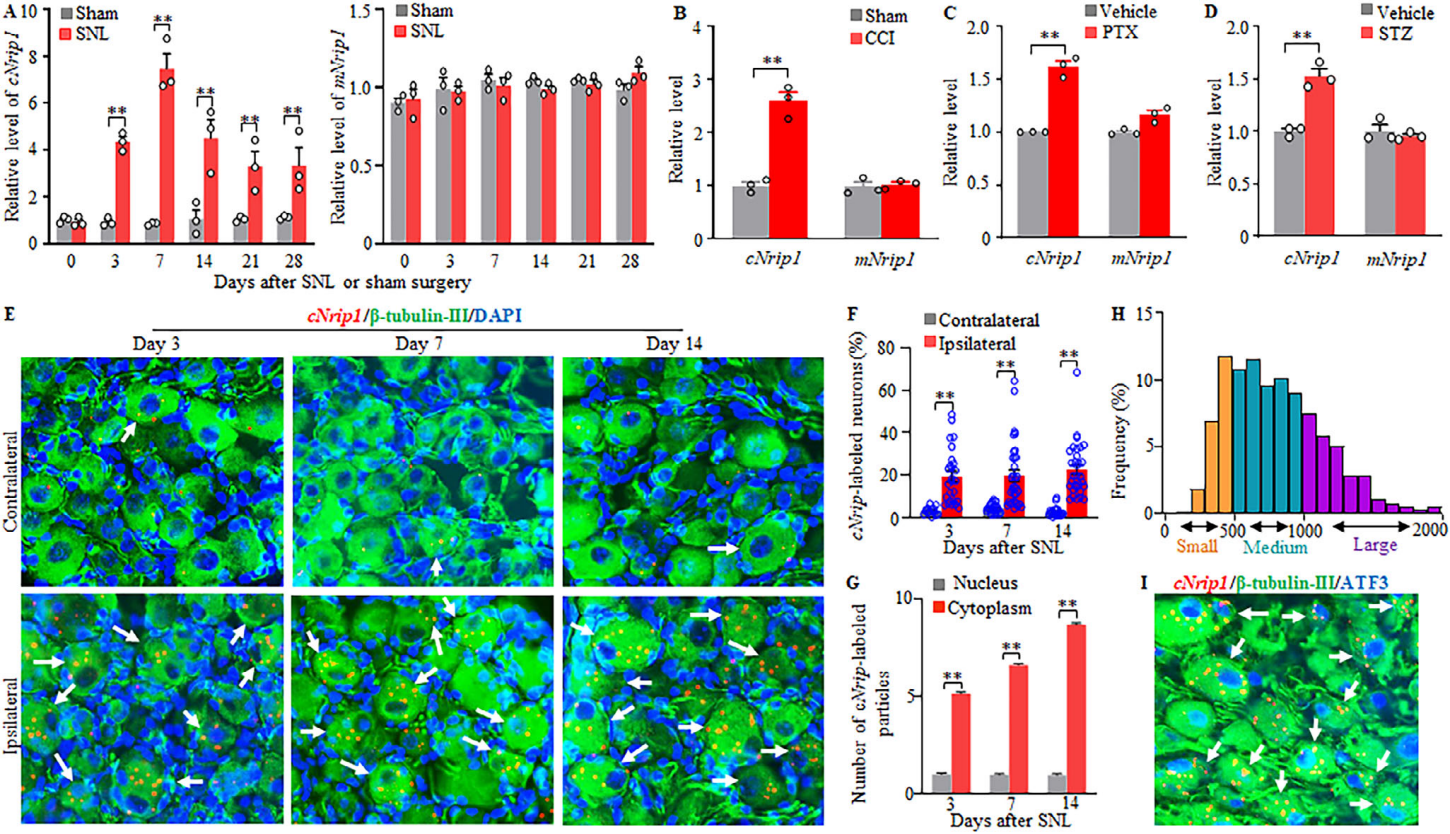
Upregulation of *cNrip1* expression in injured DRG after peripheral nerve injury. (A) Levels of *cNrip1* and *mNrip1* in the ipsilateral L4 DRG at different days after SNL or sham surgery. *n* = 3 repeats (12 mice)/time point/group. **p < 0.01 by 2‐way ANOVA followed by post hoc Tukey's test. (B) Levels of *cNrip1* and *mNrip1* in the ipsilateral L3/4 DRGs 7 days after CCI or sham surgery. *n* = 3 repeats (6 mice)/group. **p < 0.01 by two‐tailed, unpaired Student's *t* test. (C and D) Levels of *cNrip1* and *mNrip1* in bilateral L3/4 DRGs 14 days after intraperitoneal injection of paclitaxel (PTX) or vehicle (C) and 4 weeks after intraperitoneal injection of streptozotocin (STZ) or vehicle (D). *n* = 3 repeats (6 mice)/group. **p < 0.01 by two‐tailed, unpaired Student's *t* test. (E–G), Representative photomicrographs (E) and corresponding statistical analysis on number of *cNrip1*‐labled neurons (F) and number particles per *cNrip1‐*labled cytoplasm (G) in the ipsilateral and contralateral L4 DRG on days 3, 7, and 14 after SNL. Arrows: *cNrip1‐*labled neurons. *n* = 5 mice/time point. **p < 0.01 by 2‐way ANOVA followed by post hoc Tukey's test. Scale bar: 40 µm. (H) Histograms showing the distribution of *cNrip1*‐labeled somas in mouse ipsilateral L4 DRG on day 7 after SNL. Small, 21%; Medium, 51%; Large, 28%. *n* = 5 mice. (I) Representative photomicrographs showing that *cNrip1* (red) was co‐expressed with activating transcriptional factor 3 (ATF3, a marker of neuronal injury, blue) and β‐tubulin III (green) in individual neurons (arrows) from the ipsilateral L4 DRG 7 days after SNL. Scale bar: 40 µm.

To validate whether the expression of DRG *cNrip1* was changed following peripheral nerve injury, we carried out quantitative RT‐PCR assay and found that the level of *cNrip1*, but not *Nrip1* mRNA, was time‐dependently upregulated in the ipsilateral L4 DRG from day 3 to day 28 post‐SNL (Figure 2A). As expected, sham surgery did not alter basal expression of *cNrip1* or *Nrip1* mRNA in the ipsilateral L4 DRG during the observation period (Figure 2A). Neither SNL nor sham surgery changes basal expression of *cNrip1* in the contralateral L4 DRG, ipsilateral L3 DRG and ipsilateral L4 spinal cord (Figure S3A–C). The level of *cNrip1*, but not *Nrip1* mRNA, was also upregulated in the ipsilateral L3/4 DRGs on day 7 after chronic constriction injury (CCI) of unilateral sciatic nerve, another nerve trauma‐induced neuropathic pain model (Figure 2B), and in bilateral L3/4 DRGs on day 14 after intraperitoneal injection of paclitaxel (PTX; a chemotherapy neuropathic pain model) and 4 weeks after intraperitoneal injection of streptozotocin (STZ; a diabetic neuropathic pain model) (Figure 2C,D). Interestingly, this upregulation was not seen in the ipsilateral L3/4 DRGs during the observation periods in animal models of chronic inflammatory pain caused by injection of complete Freund's adjuvant into the unilateral hind paw or intra‐articular injection of sodium monoiodoacetate into the unilateral knee joint (Figure S3D,E) and in an animal model of postoperative pain caused by plantar incision of the unilateral hind paw (Figure S3F).

Consistently, the number of *cNrip1‐*labelled neurons and number of particles in the cytoplasm of each labelled neuron in the ipsilateral L4 DRG were substantially increased compared to those in the corresponding contralateral L4 DRG on days 3, 7, and 14 after SNL (Figure 2E–G). As expected, there were no differences in the numbers of particles in the nucleus of each labelled neuron between the ipsilateral and contralateral L4 DRGs during the observation period after SNL (Figure S4). A cross‐sectional area analysis of labeled neuronal soma revealed that approximately 20.82% of *cNrip1‐*labelled neurons were small in the size, 51.22% medium in the size and 27.96% large in the size in injured DRG (Figure 2H). Importantly, most *cNrip1‐*labelled neurons were positive for activating transcriptional factor 3 (ATF3, an injury marker) in the ipsilateral L4 DRG post‐SNL (Figure 2I). These results suggest that nerve injury‐induced upregulation of *cNrip*1 occurs predominantly in injured DRG neurons and may have a functional role in neuropathic pain.

### 2.3 FUS Participates in Nerve Injury‐Induced Upregulation of DRG *cNrip*1

**FIGURE 3:**
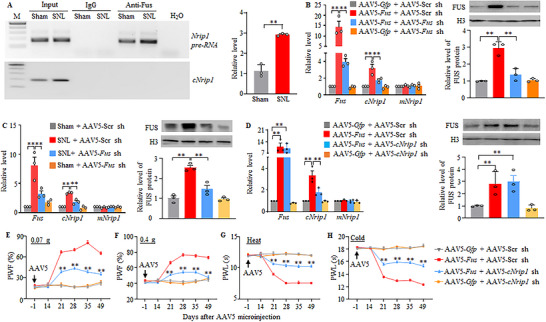
FUS promotes *cNrip1* biogenesis in injured DRG after peripheral nerve injury. (A) The fragment of *Nrip1 pre‐*RNA or *cNrip1* immunoprecipitated by anti‐FUS antibody in the ipsilateral L4 DRG 7 days after SNL or sham surgery. *n* = 3 repeats (30 mice)/group. **p < 0.01 by two‐tailed, unpaired Student's *t* test. (B) Levels of *Fus* mRNA, *cNrip1, mNrip1* and FUS protein in cultured DRG neurons with AAV5 transductions as indicated. AAV5 expressing GFP (AAV5‐*Gfp*), full‐length *Fus* mRNA (AAV5‐*Fus*), control scrambled shRNA (AAV5‐Scr sh) or *Fus* shRNA (AAV5‐*Fus* sh). *n* = 3 repeats/treatment. **p < 0.01 by 1‐way ANOVA followed by post hoc Tukey's test. (C) Levels of *Fus* mRNA, *cNrip1, mNrip1*, and FUS protein in the ipsilateral L4 DRG 14 days after SNL or sham surgery in mice with pre‐microinjection of AAV5‐Scr sh or AAV5‐*Fus* sh into the ipsilateral L4 DRG 35 days before surgery. *n* = 3 repeats (12 mice)/group/assay. **p < 0.01 by 2‐way ANOVA followed by post hoc Tukey's test. (D) Levels of *Fus* mRNA, *cNrip1, mNrip1* and FUS protein in the ipsilateral L3/4 DRG 49 days after microinjection of AAV5‐*Gfp*, AAV5‐*Fus*, AAV5‐Scr sh or AAV5‐*cNrip1* sh into the unilateral L3/4 DRGs. *n* = 3 repeats (6 mice)/group/assay. **P < 0.01 by 1‐way ANOVA followed by post hoc Tukey's test. (E–H), Effect of microinjection of AAV5‐*cNrip1* sh or AAV5‐Scr sh into the ipsilateral L3/4 DRGs on DRG FUS overexpression (through microinjection of AAV5‐*Fus* into the ipsilateral L3/4 DRGs)‐induced increases in paw withdrawal frequencies (PWFs) to 0.07 g (E) and 0.4 g (F) von Frey filaments and reductions in paw withdrawal latencies (PWLs) to heat (G) and cold (H) stimuli on the ipsilateral sides at time points as shown in male mice. n = 9 mice/group. **p < 0.01 versus the AAV5‐*Fus* plus AAV5‐Scr sh group at the corresponding days by 2‐way ANOVA with repeated measures followed by post hoc Tukey test.

We next examined how peripheral nerve injury upregulated *cNrip1* expression in injured DRG. RNA‐binding proteins (RBPs) regulate the formation of circRNA through forming RNA‐protein complexes [24, 25, 26]. To search the RBPs that trans‐act the biogenesis of *cNrip1*, we analyzed RNA binding sites in intron 1 and intron 3 sequences of *Nrip1* gene by using RBPSuite webserver (https://bio.tools/rbpsuite) and identified 9 RBPs (Figure S5). Among these predicted RBPs, only *Fused in sarcoma (Fus)* mRNA was significantly upregulated in the ipsilateral L4 DRG after SNL (Figure S5), consistent with the previous studies [27, 28]. The interaction of FUS with intron 1 of *Nrip1* pre‐RNA was readily detected by electrophoretic mobility shift assay (EMSA; Figure S6). Moreover, our chromatin immunoprecipitation (ChIP) assay further showed that intron 1 or 3 of *Nrip1* pre‐RNA containing FUS‐binding motif could be amplified from the complex immunoprecipitated with FUS antibody from sham DRGs (Figure 3A). SNL increased the binding of FUS to this fragment, as evidenced by a 2.9‐fold increase in binding activity in the ipsilateral L4 DRG on day 7 post‐SNL (Figure 3A). This increase was attributed to time‐dependent elevations of *Fus* mRNA and FUS protein in injured DRG following peripheral nerve trauma including SNL [27, 28]. As expected, FUS did not bind to mature *cNrip1* (Figure 3A).

We further investigated the effect of FUS overexpression on *cNrip1* expression in cultured DRG neurons by transducing them with AAV5 carrying full‐length *Fus* mRNA (AAV5‐*Fus*). AAV5‐*Fus*, not control AAV5‐*Gfp*, significantly increased the level of *cNrip1*, but not *Nrip1* mRNA, in the cultured DRG neurons co‐transduced with AAV5 expressing control scrambled shRNA (AAV5‐Scr shRNA; Figure 3B). This increase was substantially blocked in the cultured DRG neurons co‐transduced with AAV5 expressing *Fus* shRNA (AAV5‐*Fus* shRNA; Figure 3B), suggesting that the *cNrip1* increase was the FUS‐specific response. Consistent with our previous report [28], blocking the SNL‐induced increases of *Fus* mRNA and FUS protein in the ipsilateral L4 DRG through DRG pre‐microinjection of AAV5‐*Fus* shRNA (but not control scrambled shRNA) significantly mitigated the SNL‐induced mechanical allodynia, heat and cold hyperalgesia on the ipsilateral (not contralateral) side and stimulation‐independent ongoing pain on day 14 post‐surgery (Figure 3C; Figure S7A–I). Given that nerve injury‐induced DRG neuronal hyperexcitability triggers the hyperactivation of dorsal horn neurons and astrocytes through enhancing the release of neurotransmitters in primary afferents under neuropathic pain conditions [29], we also found that DRG pre‐microinjection of AAV5‐*Fus* shRNA prevented the SNL‐induced dorsal horn neuronal and astrocyte hyperactivities, as indicated by the absence of SNL‐induced increases in the levels of p‐ERK1/2 (a marker for neuronal hyperactivation) and GFAP (a marker for astrocyte hyperactivation), respectively, on day 14 post‐SNL on the ipsilateral side (Figure S7J). More importantly, DRG pre‐microinjection of AAV5‐*Fus* shRNA attenuated the SNL‐induced increases in the level of *cNrip1* (but not *Nrip1* mRNA) in the ipsilateral L4 DRG on day 14 post‐SNL (Figure 3C). In addition, the mice with DRG pre‐microinjection of AAV5*‐Fus* (but not AAV5*‐Gfp*) plus AAV5‐Scr shRNA displayed the increases in the amounts of *Fus* mRNA, *cNrip1* and FUS protein (but not *Nrip* mRNA) in the microinjected L3/4 DRGs, enhanced responses to mechanical, heat and cold stimuli on the ipsilateral side, ongoing pain and spinal cord dorsal horn neuronal and astrocyte hyperactivity (Figure 3D–H and Figure S8). These changes except for the increases of *Fus* mRNA and FUS protein were significantly attenuated in the mice with DRG pre‐microinjection of AAV5*‐Fus* (but not AAV5*‐Gfp*) plus AAV5 expressing *cNrip1* shRNA (AAV5‐*cNrip1* shRNA; Figure 3D–H; Figure S8). As expected, DRG microinjection of neither virus changed basal paw withdrawal responses on the contralateral side (Figures S7E–G and S8A–C) and locomotor function (Table S1). Given that *Fus* mRNA is co‐expressed with *cNrip1* in the same individual small‐, medium‐, and large‐sized DRG neurons (Figure S9), our findings suggest that nerve injury‐induced upregulation of *cNrip1* is attributable, at least in part, to an increase of FUS expression in injured DRG.

### 2.4 Blocking Upregulated DRG *cNrip1* Mitigates Neuropathic Pain

**FIGURE 4:**
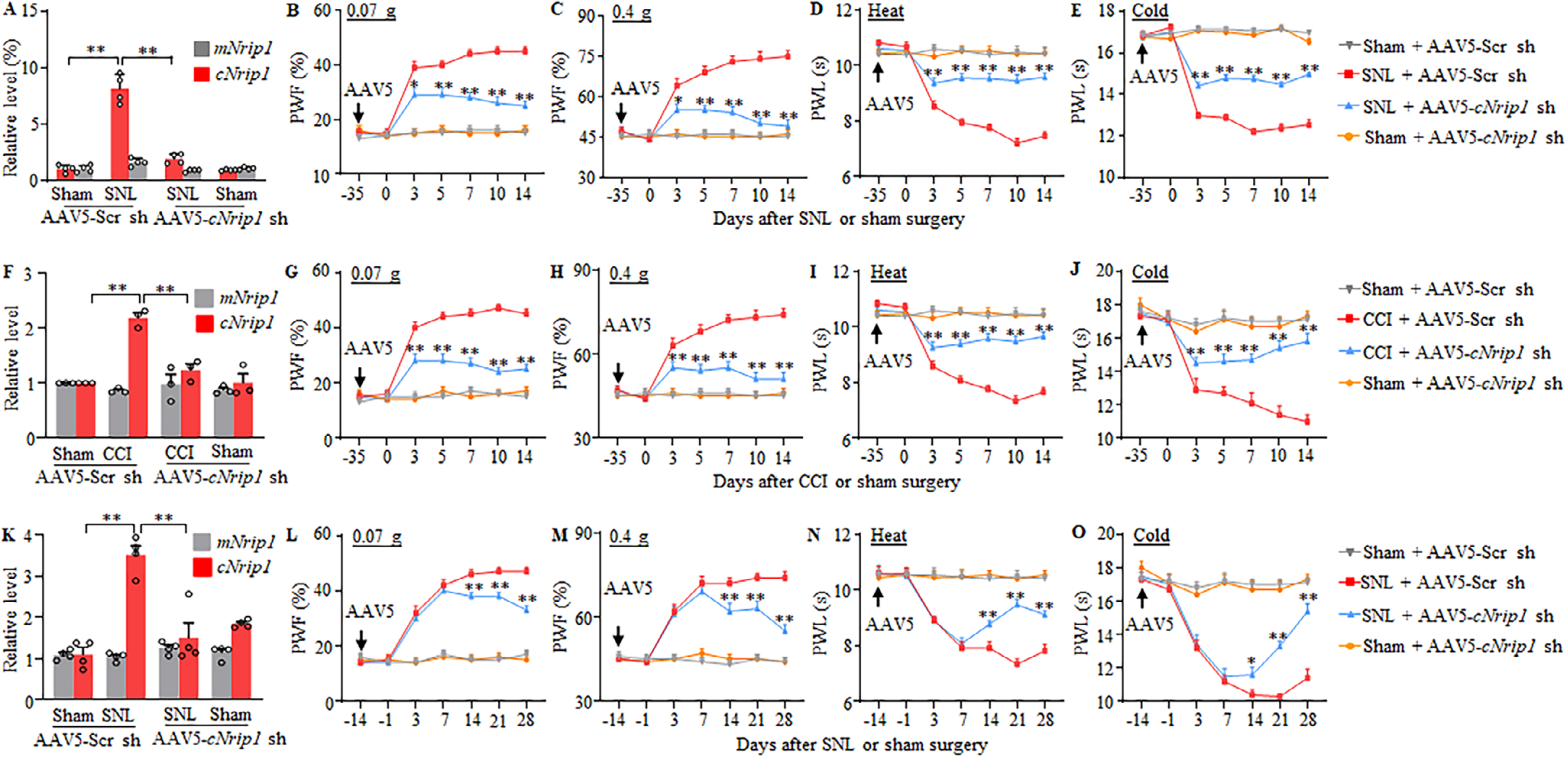
Blocking *cNrip1* upregulation in injured DRG mitigates the development and maintenance of nerve injury‐induced nociceptive hypersensitivity in male mice. (A) Levels of *cNrip1* and *mNrip1* in the ipsilateral L4 DRG 14 days after SNL or sham surgery in mice with pre‐microinjection of AAV5 expressing *cNrip1* shRNA (AAV5‐*cNrip1* sh) or control scrambled shRNA (AAV5‐Scr sh) into the ipsilateral L4 DRG 35 days before surgery. n = 4 repeats (12 mice)/group. **p < 0.01 by 2‐way ANOVA followed by post hoc Tukey's test. (B–E) Effect of pre‐microinjection of AAV5‐*cNrip1* sh or AAV5‐Scr sh into the ipsilateral L4 DRG 35 days before SNL or sham surgery on paw withdrawal frequency (PWF) in response to 0.07 g (B) and 0.4 g (C) von Frey filament stimuli and paw withdrawal latency (PWL) in response to heat (D) and cold (E) stimuli at indicated days after surgery on the ipsilateral side. n = 8 mice /group. *p < 0.05, **p < 0.01 versus the AAV5‐Scr sh‐microinjected SNL mice at the corresponding days by 3‐way ANOVA with repeated measures followed by post hoc Tukey's test. (F) Levels of *cNrip1* and *mNrip1* in the ipsilateral L3/4 DRGs 14 days after CCI or sham surgery in mice with pre‐microinjection of AAV5‐*cNrip1* sh or AAV5‐Scr sh into the ipsilateral L3/4 DRGs 35 days before surgery. n = 3 repeats (6 mice)/group. **P < 0.01 by 2‐way ANOVA followed by post hoc Tukey's test. (G–J) Effect of pre‐microinjection of AAV5‐*cNrip1* sh or AAV5‐Scr sh into the ipsilateral L3/4 DRGs 35 days before CCI or sham surgery on paw withdrawal frequency in response to 0.07 g (G) and 0.4 g (H) von Frey filament stimuli and paw withdrawal latency in response to heat (I) and cold (J) stimuli at indicated days after surgery on the ipsilateral side. n = 8 mice/group. **p < 0.01 versus the AAV5‐Scr sh‐microinjected CCI mice at the corresponding days by 3‐way ANOVA with repeated measures followed by post hoc Tukey's test. (K) Levels of *cNrip1* and *mNrip1* in the ipsilateral L4 DRG 28 days after SNL or sham surgery in mice with pre‐microinjection of AAV5‐*cNrip1* sh or AAV5‐Scr sh into the ipsilateral L3/4 DRGs 14 days before surgery. n = 4 repeats (12 mice)/group. **p < 0.01 by 2‐way ANOVA followed by post hoc Tukey's test. (L–O) Effect of pre‐microinjection of AAV5‐*cNrip1* sh or AAV5‐Scr sh into the ipsilateral L4 DRG 14 days before SNL or sham surgery on paw withdrawal frequency in response to 0.07 g (L) and 0.4 g (M) von Frey filament stimuli and paw withdrawal latency in response to heat (N) and cold (O) stimuli at indicated days after surgery on the ipsilateral side. n = 8 mice /group. *p < 0.05, **p < 0.01 versus the AAV5‐Scr sh‐microinjected SNL mice at the corresponding days by 3‐way ANOVA with repeated measures followed by post hoc Tukey's test.

**FIGURE 5:**
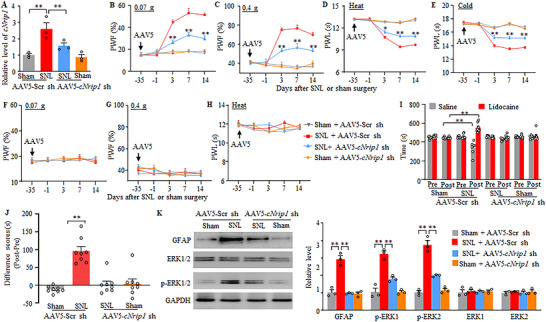
Blocking *cNrip1* upregulation in injured DRG mitigates the development of SNL‐induced nociceptive hypersensitivity in female mice. (A) Level of *cNrip1* in the ipsilateral L4 DRG 14 days after SNL or sham surgery in mice with pre‐microinjection of AAV5‐*cNrip1* sh or AAV5‐Scr sh into the ipsilateral L4 DRG 35 days before surgery. n = 3 repeats (12 mice)/group. **p < 0.01 by 2‐way ANOVA followed by post hoc Tukey's test. (B–H) Effect of pre‐microinjection of AAV5‐*cNrip1* sh or AAV5‐Scr sh into the ipsilateral L4 DRG 35 days before SNL or sham surgery on paw withdrawal frequency (PWF) in response to 0.07 g (B and F) and 0.4 g (C and G) von Frey filament stimuli and paw withdrawal latency (PWL) in response to heat (D and H) and cold (E) stimuli on the ipsilateral (B–E) and contralateral (F–H) sides at indicated days after surgery. n = 6 mice/group. *p < 0.05, **p < 0.01 versus the AAV5‐Scr sh‐microinjected SNL mice at the corresponding days by 3‐way ANOVA with repeated measures followed by post hoc Tukey's test. (I,J) Effect of pre‐microinjection of AAV5‐*cNrip1* sh or AAV5‐Scr sh into the ipsilateral L4 DRG 35 days before SNL or sham surgery on spontaneous ongoing pain as assessed by the conditional place preference paradigm 14 days after surgery. Pre, preconditioning. Post, post‐conditioning. *n =* 6 mice/group. **p < 0.01 by 3‐way (I) or 2‐way (J) ANOVA with repeated measures followed by post hoc Tukey's test. (K) Levels of p‐ERK1/2, total ERK1/2 and GFAP in the ipsilateral L4 dorsal horn on day 14 after SNL or sham surgery in mice with pre‐microinjection of AAV5‐Scr sh or AAV5‐*cNrip1* sh into the ipsilateral L4 DRG 35 days before surgery. n = 3 repeats (3 mice)/group. **p < 0.01 by 2‐way ANOVA followed by Tukey post hoc test.

To investigate whether the upregulated *cNrip1* in injured DRG contributes to the development of neuropathic pain, we blocked the SNL‐induced increase of DRG *cNrip1* through pre‐microinjection of AAV5‐*cNrip1* shRNA into the ipsilateral L4 DRG 35 days before SNL/sham surgery in male mice, as AAV5 takes 3–4 weeks for target genes to be expressed in the DRG [30, 31, 32]. This microinjection not only blocked the SNL‐induced increase of *cNrip1* expression in the ipsilateral L4 DRG on day 14 post‐ SNL (Figure 4A) but also alleviated the SNL‐induced mechanical, heat and cold nociceptive hypersensitivities on days 3, 5, 7, 10, and 14 after SNL (Figure 4B–E) and the SNL‐induced dorsal horn neuronal and astrocyte hyperactivities on day 14 post‐SNL (Figure S10A) on the ipsilateral side. Microinjection of control AAV5‐Scr shRNA did not exhibit these effects (Figure 4B–E; Figure S10A). Neither viral microinjection altered basal levels of *cNrip1* and *Nrip1* mRNA in the ipsilateral L4 DRG of sham‐operated male mice and basal mechanical, heat and cold responses on the contralateral side of the SNL mice and on both sides of the sham‐operated male mice (Figure 4A–E; Figure S10B–D). The similar results were observed in male CCI mice (Figure 4F–J; Figure S11A–C) and female SNL mice (Figure 5A–K) pre‐microinjected with AAV5‐*cNrip1* shRNA or AAV5‐Scr shRNA 35 days before surgery. Locomotor function was normal in these microinjected mice (Table S1). Taken together, our findings suggest that DRG upregulated *cNrip1* participates in the development of nerve injury‐induced neuropathic pain in both male and female mice.

We also examined the role of DRG upregulated *cNrip1* in the maintenance of SNL‐induced neuropathic pain. AAV5‐*cNrip1* shRNA or AAV5‐Scr shRNA was microinjected into the ipsilateral L4 DRG 14 days before SNL or sham surgery in male mice. As expected, SNL‐induced increase of *cNrip1* in the ipsilateral L4 DRG from the AAV5‐Scr shRNA‐microinjected mice was not seen in the AAV5‐*cNrip1* shRNA‐microinjected mice on day 28 post‐SNL (Figure 4K). Mechanical, heat and cold nociceptive hypersensitivities were fully developed on the ipsilateral (not contralateral) side in both AAV5‐*cNrip1* shRNA‐ and AAV5‐Scr shRNA‐microinjected mice on days 3 and 7 post‐SNL (Figure 4L–O; Figure S11D–F). These nociceptive hypersensitivities were significantly alleviated from days 14 to 28 post‐SNL in the AAV5‐ *cNrip1* shRNA‐microinjected mice (Figure 4L–O). Collectively, the evidence indicates an important role of DRG upregulated *cNrip1* in the maintenance of nerve injury‐induced neuropathic pain.

### 2.5 Mimicking Nerve Injury‐Induced DRG *cNrip1* Upregulation Produces Nociceptive Hypersensitivity

**FIGURE 6:**
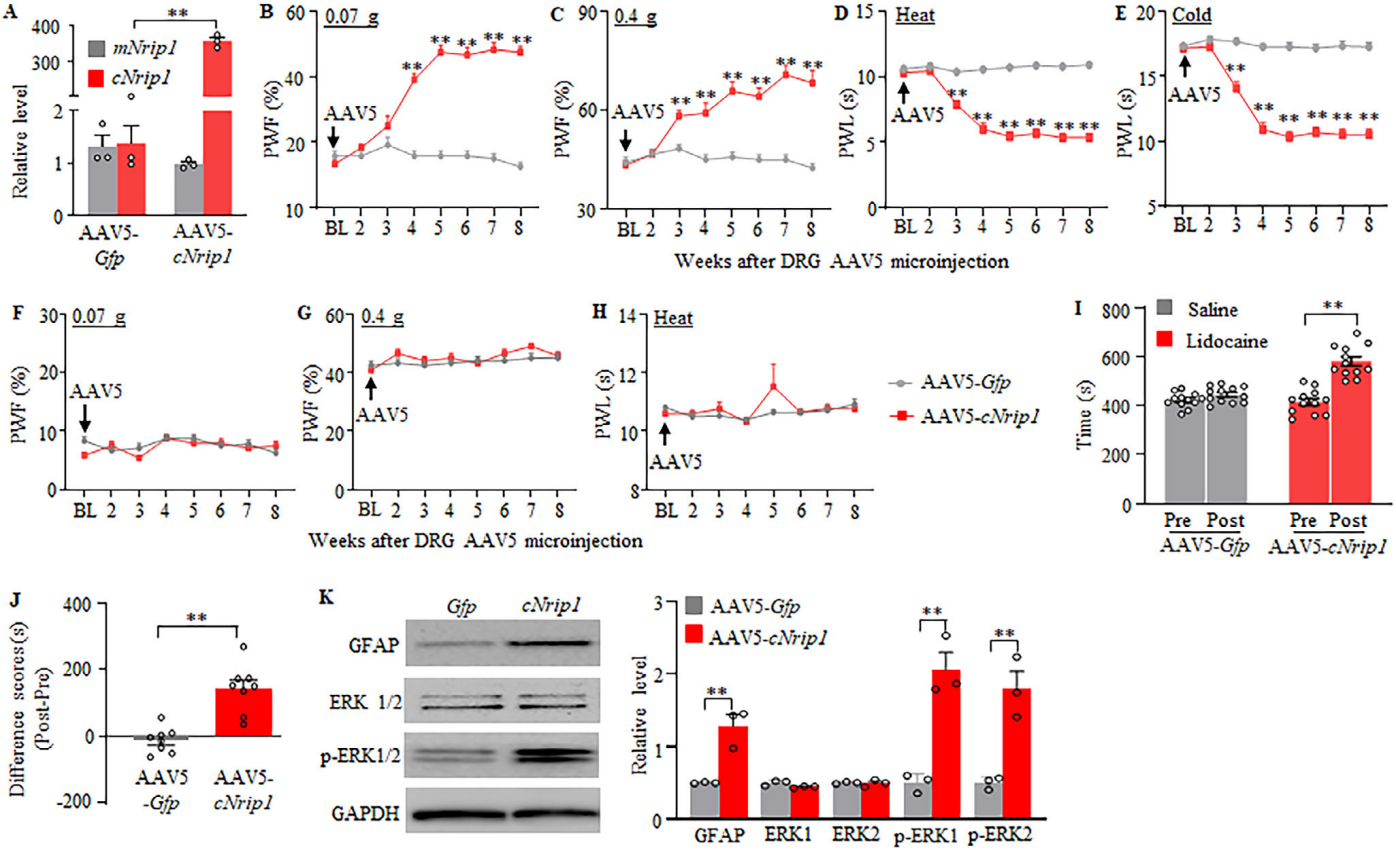
DRG overexpression of *cNrip1* produces neuropathic pain–like symptoms in naive male mice. (A) Levels of *cNrip1* and *mNrip1* in the ipsilateral L3/4 DRGs 8 weeks after microinjection of AAV5 expressing the full‐length *cNrip1* (AAV5‐*cNrip1*) or green fluorescent protein (AAV5‐*Gfp*) into unilateral L3/4 DRGs. *n* = 3 repeats (6 mice)/group. **p < 0.01 by two‐tailed, unpaired Student's *t* test. (B–H) Effect of microinjection of AAV5‐*cNrip1* or AAV5‐*Gfp* into the ipsilateral L3/4 DRGs on paw withdrawal frequency (PWF) in response to 0.07 g (B and F) and 0.4 g (C and G) von Frey filament stimuli and paw withdrawal latency (PWL) in response to heat (D and H) and cold (E) stimuli at indicated weeks after microinjection on the ipsilateral (C–E) and contralateral (F–H) sides. *n* = 12 mice/group. **p < 0.01 versus the AAV5‐*Gfp*‐microinjected mice at the corresponding weeks by 2‐way ANOVA with repeated measures followed by post hoc Tukey's test. (I and J) Effect of microinjection of AAV5‐*cNrip1* or AAV5‐*Gfp* into the ipsilateral L3/4 DRGs on spontaneous ongoing pain as assessed by the conditional place preference paradigm 7 weeks after microinjection. Pre, preconditioning. Post, post‐conditioning. *n* = 12 mice/group. **p < 0.01 by 2‐way ANOVA with repeated measures followed by post hoc Tukey's test (I) or two‐tailed, unpaired Student's *t* test (J). (K) Effect of microinjection of AAV5‐*cNrip1* or AAV5‐*Gfp* into the unilateral L3/4 DRGs on levels of p‐ERK1/2, ERK1/2, and GFAP in the ipsilateral L3/4 dorsal horn 8 weeks after microinjection. *n* = 3 repeats (3 mice)/group. **P < 0.01 by two‐tailed, unpaired Student's *t* test.

**FIGURE 7:**
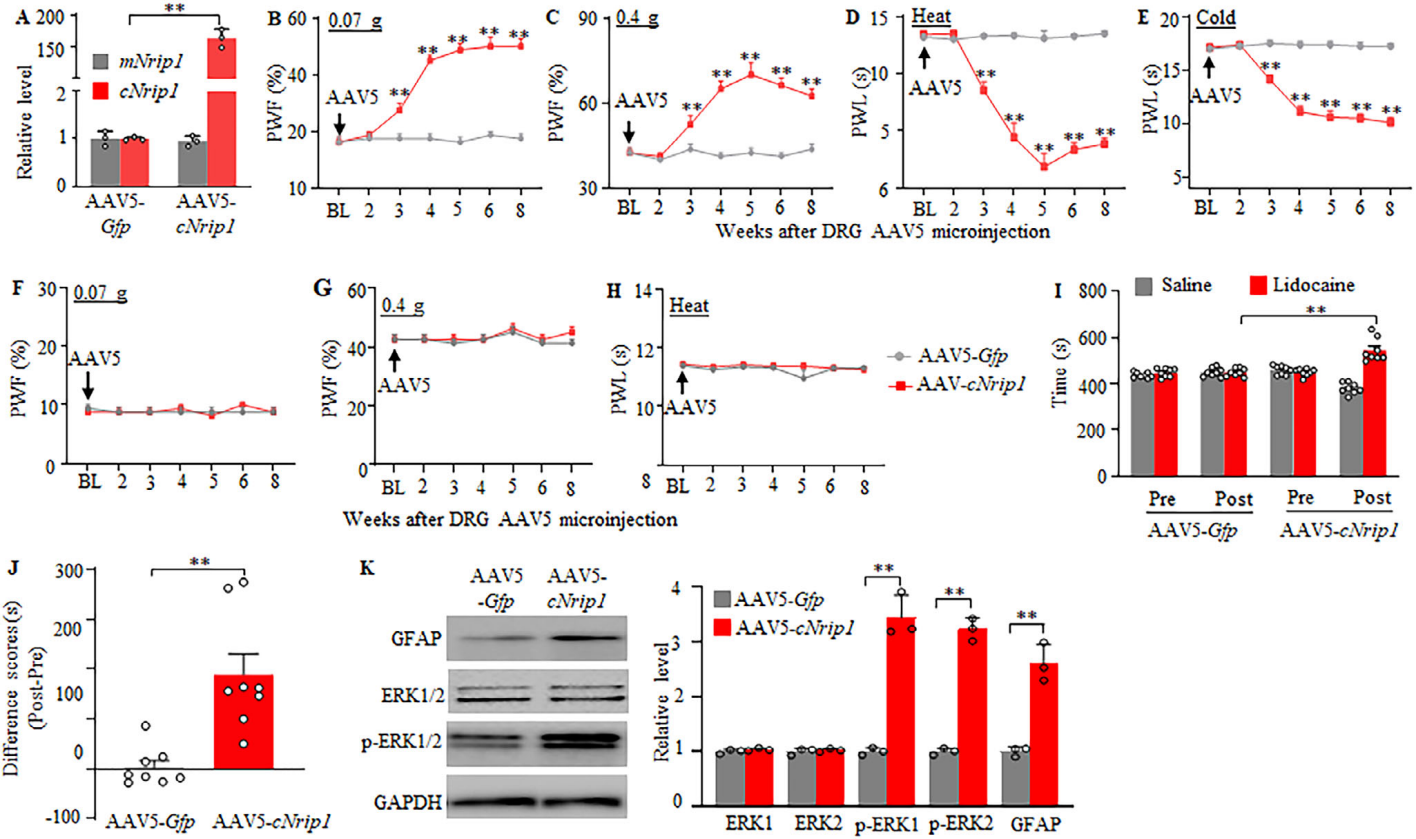
DRG overexpression of *cNrip1* produces neuropathic pain–like symptoms in naive female mice. (A) Levels of *cNrip1* and *mNrip1* in the ipsilateral L3/4 DRGs 8 weeks after microinjection of AAV5‐*Gfp* or AAV5–*cNrip1* into unilateral L3/4 DRGs. *n =* 3 repeats (12 mice)/group. **p < 0.01 by two‐tailed, unpaired Student's *t* test. (B–H) Effect of microinjection of AAV5‐*cNrip1* or AAV5‐*Gfp* into the ipsilateral L3/4 DRGs on paw withdrawal frequency (PWF) in response to 0.07 g (B and F) and 0.4 g (C and G) von Frey filament stimuli and paw withdrawal latency (PWL) in response to heat (D and H) and cold (E) stimuli on the ipsilateral (B–E) and contralateral (F–H) sides at indicated weeks after microinjection. *n =* 12 mice/group. **p < 0.01 versus the AAV5‐*Gfp*–microinjected mice at the corresponding weeks by 2‐way ANOVA with repeated measures followed by post hoc Tukey's test. (I,J) Effect of microinjection of AAV5‐*cNrip1* or AAV5‐*Gfp* into the ipsilateral L3/4 DRGs on spontaneous ongoing pain as assessed by the conditional place preference paradigm 8 weeks after microinjection. Pre, preconditioning; Post, post‐conditioning. *n =* 12 mice/group. **p < 0.01, by 2‐way ANOVA followed by post hoc Tukey's test (I) or two‐tailed, unpaired Student's *t* test (J). (K) Effect of microinjection of AAV5‐*cNrip1* or AAV5‐*Gfp* into the unilateral L3/4 DRGs on levels of p‐ERK1/2, ERK1/2, and GFAP in the ipsilateral L3/4 dorsal horn 8 weeks after microinjection. *n =* 6 mice /group. **p < 0.01 by two‐tailed, unpaired Student's *t* test.

We further asked whether DRG *cNrip1* upregulation was sufficient for neuropathic pain. To this end, we mimicked the nerve injury‐induced upregulation of DRG *cNrip1* through microinjection of AAV5 expressing full‐length *cNrip1* (AAV5‐*cNrip1*) into unilateral L3/4 DRGs of naïve male mice. AAV5*‐Gfp* was used as a control. As expected, the level of *cNrip1*, but not *Nrip1* mRNA, was significantly increased in microinjected DRGs 8 weeks after DRG microinjection of AAV5‐*cNrip1*, compared to the AAV5*‐Gfp*‐treated mice (Figure 6A). DRG microinjection of AAV5‐*cNrip1* led to the increases in paw withdrawal frequencies to mechanical stimuli and the decreases in paw withdrawal latencies to heat or cold stimulation on microinjected side (Figure 6B–E). These behavioral changes occurred between 3 and 4 weeks post‐microinjection and lasted for at least 8 weeks (Figure 6B–E). Neither viral microinjection altered basal paw withdrawal responses on non‐microinjected side (Figure 6F–H) and normal locomotor function (Table S1). DRG microinjection of AAV5‐*cNrip1* also produced stimulation‐independent spontaneous ongoing pain (evidenced by a robust preference for the lidocaine‐paired chamber) 7 weeks post‐microinjection on microinjected side (Figure 6I,J). Neuronal and astrocyte hyperactivities indicated by robust increases in the levels of p‐ERK1/2 and GFAP, respectively, were also detected in the ipsilateral L3/4 dorsal horn 8 weeks after microinjection of AAV5‐*cNrip1* (Figure 6K). The results were similar in female mice with DRG microinjection of AAV5‐*cNrip1* (Figure 7A–K). Together, these results suggest that DRG *cNrip1* upregulation produces neuropathic pain‐like symptoms in the absence of nerve injury.

### 2.6 Upregulated *cNrip1* Is Responsible for Nerve Injury‐Induced TLR2 Activation in Injured DRG

To explore the mechanism of how DRG *cNrip1* upregulation contributes to neuropathic pain, we performed high‐throughput RNA sequencing to identify potential downstream targets regulated by *cNrip1* in injured DRG after SNL. The unbiased gene expression database revealed that about 6,470 genes out of a total of 19,403 identified genes were significantly changed in the ipsilateral L4 DRG from the AAV5‐Scr RNA‐microinjected mice on day 7 post‐SNL as compared to that post‐sham surgery (Figure S12A). Approximately 45.3% of these changed genes were upregulated and 54.7% downregulated (Figure S12A). Among these changed genes, 1,021 upregulated genes were blocked in the ipsilateral L4 DRG from the AAV5‐*cNrip1* shRNA‐microinjected mice on day 7 post‐SNL (Figure S12B). These affected genes are notably enriched for the cytokine/inflammation/Toll‐like receptor signaling pathway (Figure S12C).

**FIGURE 8:**
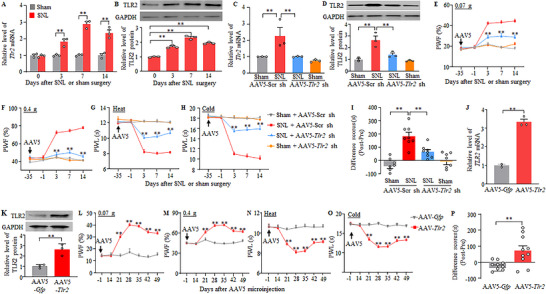
Nerve injury‐induced increase of TLR2 in injured DRG is required for the development of nerve injury‐induced nociceptive hypersensitivity. (A,B) Levels of *Tlr2* mRNA (A) and TLR2 protein (B) in the ipsilateral L4 DRG on days 0, 3, 7 and 14 after SNL and sham surgery. n = 3 repeats (6 mice)/time point/group. **p < 0.01 by two‐way ANOVA followed by Tukey post hoc test. (C,D) Levels of *Tlr2* mRNA (C) and TLR2 protein (D) in the ipsilateral L4 DRG 14 days after SNL or sham surgery in male mice with pre‐microinjection of AAV5 expressing *Tlr2* shRNA (AAV5‐*Tlr2* sh) or control scrambled shRNA (AAV5‐Scr sh) into the ipsilateral L4 DRG 35 days before surgery. *n* = 3 repeats (12 mice)/group/assay. **p < 0.01 by 2‐way ANOVA followed by post hoc Tukey's test. (E–H) Effect of pre‐microinjection of AAV5‐*Tlr2* sh or AAV5‐Scr sh into the ipsilateral L4 DRG 35 days before SNL or sham surgery on paw withdrawal frequency (PWF) in response to 0.07 g (E) and 0.4 g (F) von Frey filament stimuli and paw withdrawal latency (PWL) in response to heat (G) and cold (H) stimuli at indicated days after surgery on the ipsilateral side. n = 9 mice/group. **p < 0.01 versus the AAV5‐Scr sh‐microinjected SNL mice at the corresponding days by 3‐way ANOVA with repeated measures followed by post hoc Tukey's test. (I) Effect of pre‐microinjection of AAV5‐*Tlr2* sh or AAV5‐Scr sh into the ipsilateral L4 DRG 35 days before SNL or sham surgery on spontaneous ongoing pain as assessed by the conditional place preference paradigm 14 days after surgery. *n* = 8 mice/group. **p < 0.01 by 2‐way ANOVA followed by post hoc Tukey's test. (J,K) Levels of *Tlr2* mRNA (J) and TLR2 protein (K) in the ipsilateral L3/4 DRGs 49 days after microinjection of AAV5 expressing the full‐length *Tlr2* mRNA (AAV5‐*Tlr2*) or AAV5‐*Gfp* into the ipsilateral L3/4 DRGs. n = 3 repeats (6 mice)/ group/assay. **p < 0.01 by two‐tailed, unpaired Student's *t* test. (L–O) Effect of microinjection of AAV5–*Tlr2* or AAV5‐*Gfp* into the ipsilateral L3/4 DRGs on paw withdrawal frequency in response to 0.07 g (L) and 0.4 g (M) von Frey filament stimuli and paw withdrawal latency in response to heat (N) and cold (O) stimuli at indicated days after microinjection on the ipsilateral side. *n* = 10 mice/group. **p < 0.01 versus the AAV5‐*Gfp*‐microinjected mice at the corresponding days by 2‐way ANOVA with repeated measures followed by post hoc Tukey's test. (P) Effect of microinjection of AAV5–*Tlr2* or AAV5‐*Gfp* into the ipsilateral L3/4 DRGs on spontaneous ongoing pain as assessed by the conditional place preference paradigm 7 weeks after microinjection. *n* = 10 mice/group. **p < 0.01 by two‐tailed, unpaired Student's *t* test.

TLR2 gene, one most striking gene among these upregulated genes, contributes to neuropathic pain genesis [4, 6, 7, 8]. RNA sequencing analysis showed that the level of *Tlr2* mRNA was significantly elevated in the ipsilateral L4 DRG on day 7 post‐SNL (Figure S12B). Quantitative RT‐PCR and Western blot assays further revealed that the amounts of *Tlr2* mRNA and TLR2 protein were time‐dependently increased in the ipsilateral L4 DRG on days 3, 7, and 14 post‐SNL (Figure 8A,B). Blocking these increases through pre‐microinjection of AAV5‐*Tlr2* shRNA into the ipsilateral L4 DRG 35 days before SNL (Figure 8C,D) alleviated the SNL‐induced mechanical, thermal and cold nociceptive hypersensitivities on the ipsilateral side on days 3, 7, and 14 post‐SNL (Figure 8E–H;) as well as stimulation‐independent spontaneous ongoing pain (Figure 8I; Figure S13A) and dorsal horn neuronal and astrocyte hyperactivities on day 14 post‐SNL (Figure S13B). Neither AAV5‐*Tlr2* shRNA nor AAV5‐scrambled shRNA altered basal expression of *Tlr2* mRNA and TLR2 protein in the ipsilateral L4 DRG of sham mice and basal behavioral responses on the contralateral side of SNL mice and either side of sham mice (Figure 8C–H; Figure S13C–E). Conversely, DRG overexpression of *Tlr2* mRNA and its protein through microinjection of AAV5 expressing full‐length *Tlr2* mRNA (AAV5‐*Tlr2*) into the unilateral L3/4 DRGs (Figure 8J,K) led to enhanced responses to mechanical, thermal and cold stimuli starting from week 3 and persisting for at least 7 weeks post‐viral microinjection on the ipsilateral (not contralateral) side (Figure 8L–O; Figure S14A–C), stimulation‐independent spontaneous ongoing pain (Figure 8P; Figure S14D) and dorsal horn neuronal and astrocyte hyperactivities on week 7 post‐viral microinjection (Figure S14E). As expected, DRG microinjection of control AAV5‐*Gfp* did not produce these effects (Figure 8L–P; Figure S14A–D). None of viral microinjections affected locomotor function (Table S1). These findings suggest that DRG TLR2 may be a key player in neuropathic pain.

**FIGURE 9:**
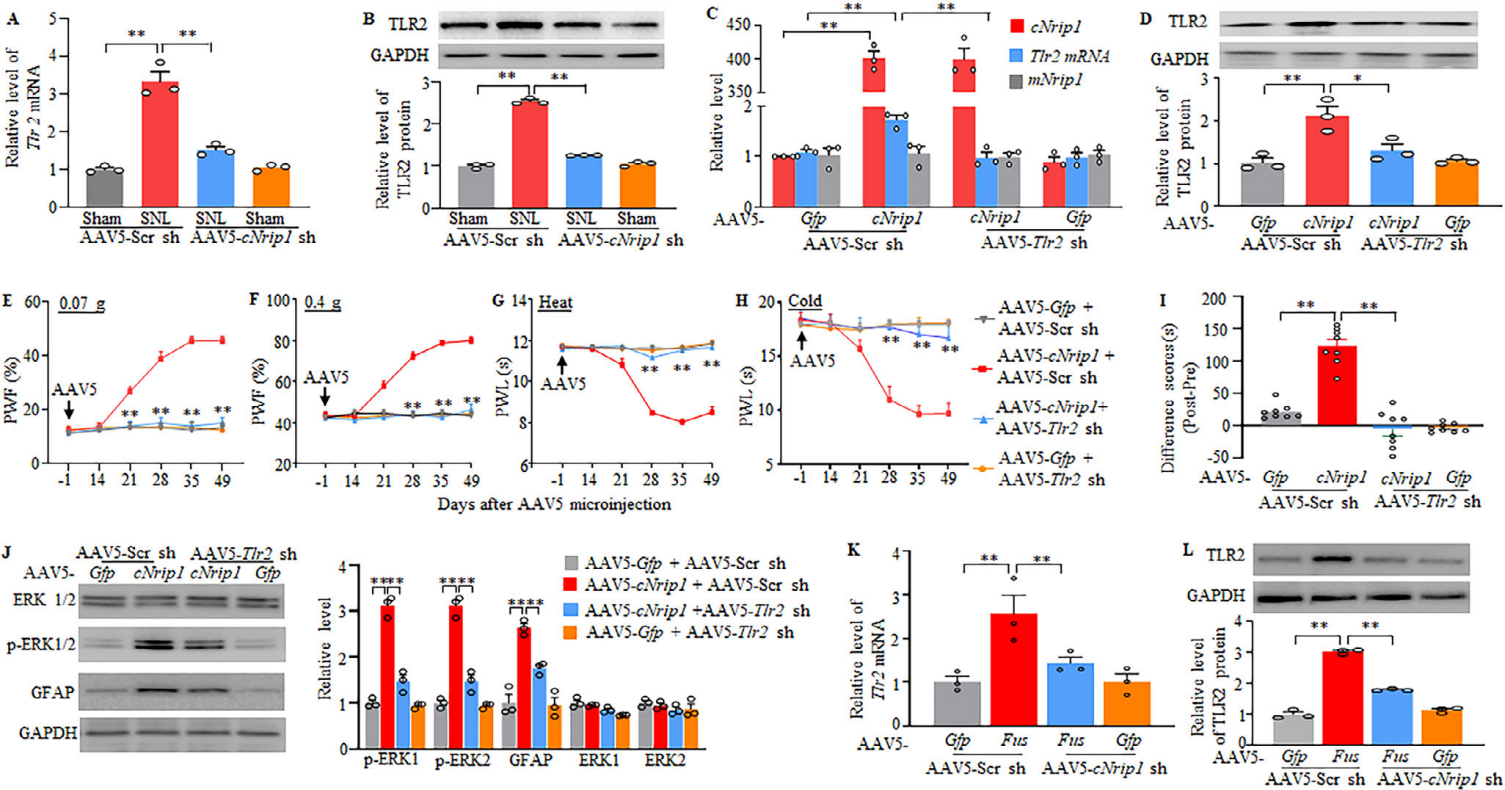
Upregulated *cNrip1* is responsible for nerve injury‐induced increase of TLR2 in injured DRG. (A,B) Levels of *Tlr2* mRNA (A) and TLR2 protein (B) in the ipsilateral L4 DRG on day 14 after SNL or sham surgery with pre‐microinjection of AAV5‐*cNrip1* sh or AAV5‐Scr sh into the ipsilateral L4 DRG 35 days before surgery. n = 3 repeats (12 mice)/ group/assay. **p < 0.01 by 2‐way ANOVA followed by Tukey post hoc test. (C,D) Levels of *Tlr2* mRNA, *cNrip1* and *mNrip1* (C), and TLR2 protein (D) in the ipsilateral L3/4 DRG 49 days after co‐microinjection of different AAV5 as indicated into the unilateral L3/4 DRGs. *n* = 3 repeats (6 mice)/group. *p < 0.05, **p < 0.01 by 1‐way ANOVA followed by post hoc Tukey's test. (E–H) Effect of microinjection of AAV5‐*Tlr2* sh or AAV5‐Scr sh into the ipsilateral L3/4 DRGs on paw withdrawal frequency (PWF) to 0.07 g (E) and 0.4 g (F) von Frey filaments and on paw withdrawal latencies (PWL) to heat (G) and cold (H) stimuli on the ipsilateral sides as shown days post‐*co*‐microinjection of AAV5‐c*Nrip1* or AAV5‐*Gfp*. n = 9 mice/group. **p < 0.01 versus the AAV5‐*cNrip1* plus AAV5‐Scr sh group at the corresponding days by 2‐way ANOVA with repeated measures followed by post hoc Tukey test. (I) Effect of microinjection of AAV5‐*Tlr2* sh or AAV5‐Scr sh into the ipsilateral L3/4 DRGs on spontaneous ongoing pain as assessed by the conditional place preference paradigm 7 weeks after co‐microinjection of AAV5‐c*Nrip1* or AAV5‐*Gfp*. *n* = 8 mice/group. **p < 0.01 by 1‐way ANOVA followed by post hoc Tukey's test. (J) Levels of p‐ERK1/2, total ERK1/2 and GFAP in the ipsilateral L3/4 dorsal horn 49 days after co‐microinjection of different AAV5 as indicated into the unilateral L3/4 DRGs. *n* = 3 repeats (3 mice)/group. **p < 0.01 by 1‐way ANOVA followed by Tukey post hoc test. (K,L) Levels of *Tlr2* mRNA (K) and TLR2 protein (L) in the ipsilateral L3/4 DRG 49 days after co‐microinjection of different AAV5 as indicated into the ipsilateral L3/4 DRGs. *n* = 3 repeats (6 mice)/group. **p < 0.01 by 1‐way ANOVA followed by post hoc Tukey's test.

RNA sequencing analysis revealed that the SNL‐induced increase of *Tlr2* mRNA was significantly attenuated in the ipsilateral L4 DRG of mice pre‐microinjected with AAV5‐*cNrip1* shRNA on day 7 post‐SNL (Figure S12B). This suggests that TLR2 is a downstream target of *cNrip1* in injured DRG under neuropathic pain conditions. Quantitative RT‐PCR and Western blot assays further confirmed that DRG microinjection of AAV5‐*cNrip1* shRNA blocked the SNL‐induced increases in the levels of *Tlr2* mRNA and TLR2 protein in the ipsilateral L4 DRG on day 14 post‐SNL (Figure 9A,B). Co‐microinjection of AAV5‐*Tlr2* shRNA and AAV5‐*cNrip1* into unilateral L3/4 DRGs of naïve mice not only blocked the *cNrip1* overexpression‐induced increases in the levels of *Tlr2* mRNA and TLR2 protein in microinjected L3/4 DRGs on day 49 post‐viral microinjection (Figure 9C,D), but also abolished the *cNrip1* overexpression‐induced mechanical, thermal and cold nociceptive hypersensitivities on weeks 3, 4, 5 and 7 post‐viral microinjection, stimulation‐independent spontaneous ongoing pain and dorsal horn neuronal and astrocyte hyperactivities on week 7 post‐viral microinjection (Figure 9E–J; Figure S15A). As predicted, this microinjection did not change the basal levels of *Tlr2* mRNA and TLR2 protein in the AAV5‐*Gfp*‐microinjected L3/4 DRGs (Figure 9C,D), basal behavioral responses in the AAV5‐*Gfp*‐microinjected mice on the ipsilateral side (Figure 9E–H) and in the AAV5‐*Gfp*‐ or AAV5‐*cNrip1*‐microinjected mice on the contralateral side (Figure S15B–D) and locomotor activity (Table S1). Furthermore, DRG overexpression of *Fus* through pre‐microinjection of AAV5‐*Fus* into the unilateral L3/4 DRGs also increased the amounts of *Tlr2* mRNA and TLR2 protein in microinjected DRGs from control AAV5‐scrambled shRNA‐microinjected mice on day 49 post‐viral microinjection (Figure 9K,L). These increases were significantly attenuated through co‐microinjection of AAV5‐*cNrip1* shRNA into the unilateral L3/4 DRGs (Figure 9K,L). These results strongly indicate that upregulated *cNrip1* contributes to nerve injury‐induced increases of *Tlr2* mRNA and its protein in injured DRG.

### 2.7 *cNrip1* Interacts With SYNCRIP to Stabilize *Tlr2* mRNA Expression in Injured DRG After Nerve Injury

**FIGURE 10:**
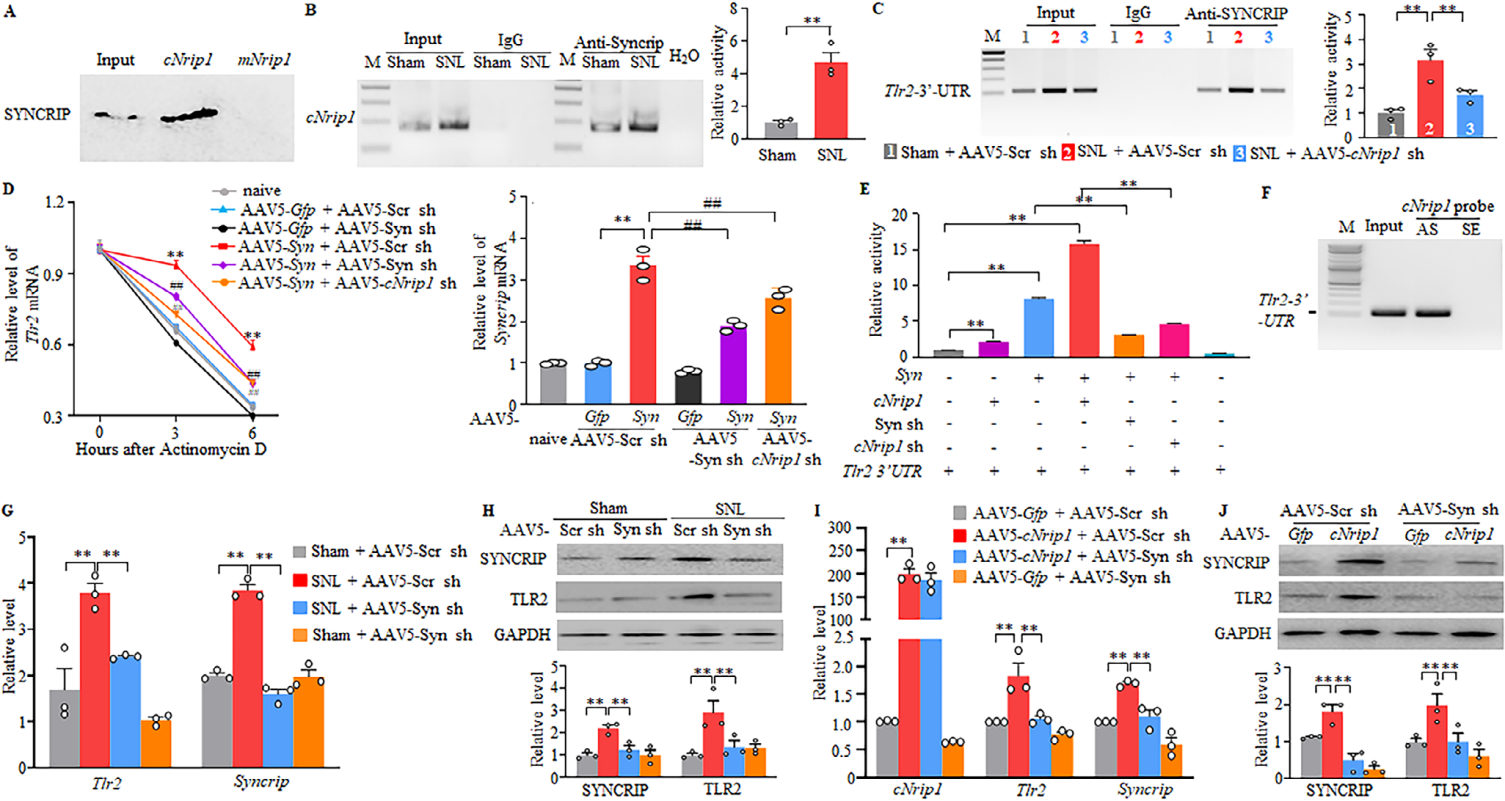
Upregulated *cNrip1* is required for SYNCRIP‐triggered stability of *Tlr2* mRNA expression in injured DRG after nerve injury. (A) SYNCRIP pulled down by the labelled exogenous *cNrip1*, but not the labelled exogenous *mNrip1*, in cultured DRG neurons. *n* = 3 repeats. (B) *cNrip1* immunoprecipitated by anti‐SYNCRIP antibody in the ipsilateral L4 DRG 7 days after SNL or sham surgery. n = 3 repeats (45 mice)/group. **p < 0.01 by 2‐tailed, unpaired Student's *t* test. (C) *Tlr2* 3’‐UTR immunoprecipitated by anti‐SYNCRIP in the ipsilateral L4 DRG 7 days after SNL or sham surgery from male mice with pre‐microinjection of AAV5‐*cNrip1* sh or AAV5‐Scr sh for 28 days before surgery. *n* = 3 repeats (45 mice)/group. **p < 0.01 by 2‐way ANOVA followed by post hoc Tukey's test. (D) CAD cells transduced by different AAV5 as shown were treated with actinomycin D (5 µg/ml) for the indicated hours. Levels of *Tlr2* mRNA at 0, 3 and 6 h and *Syncrip* mRNA at 6 h after the treatment of actinomycin D were analyzed using quantitative RT‐PCR. AAV5‐*Gfp* expressing GFP. AAV5‐*Syn e*xpressing full‐length *Syncrip* mRNA. AAV5‐Scr sh expressing control scrambled shRNA. AAV5‐cNrip1 sh expressing *cNrip1* shRNA. *n* = 3 repeats/group/time point. **p < 0.01 versus naïve group. ##*p* < 0.01 versus the AAV5‐Syn plus AAV5‐Scr sh‐treated group. 1 (for *Syncrip* mRNA) and 2 (for *Tlr2* mRNA)‐way ANOVA followed by post hoc Tukey's test. (E) *Tlr2* 3’‐UTR activities in HEK 293T cells transfected as shown. Syn: vector expressing full‐length *Syncrip* mRNA. c*Nrip1*: vector expressing full‐length *cNrip1*. Syn sh: vector expressing *Syncrip* shRNA. cNrip1 sh: vector expressing *cNrip1* shRNA. *Tlr2* 3’‐UTR: vector expressing *Tlr2* 3’‐UTR. *n* = 3 repeats/group **p < 0.01 1‐way ANOVA followed by post hoc Tukey's test. (F) *Tlr2* 3’‐UTR pulled down by *cNrip1* antisense (AS), but not sense (SE), RNA probes in cultured DRG neurons. (G,H) Levels of *Tlr2* and *Syncrip* mRNAs (G) and TLR2 and SYNCRIP proteins (H) in the ipsilateral L4 DRG 14 days after SNL or sham surgery in male mice with pre‐microinjection of AAV5 expressing *Syncrip* shRNA (AAV5‐Syn sh) or control scrambled shRNA (Scr sh) into the ipsilateral L4 DRG for 35 days before surgery. *n* = 3 repeats (12 mice) /group/assay. **p < 0.01 by 2‐way ANOVA followed by post hoc Tukey's test. (I,J) Levels of *cNrip1, Tlr2* and *Syncrip* mRNAs (I) and TLR2 and SYNCRIP proteins (J) in the ipsilateral L3/4 DRG 5 weeks after microinjection of different AAV5 as indicated into the ipsilateral L3/4 DRGs. *n* = 3 repeats per treatment. **p < 0.01 by 1‐way ANOVA followed by post hoc Tukey's test.

Finally, we asked how the upregulated *cNrip1* elevated the level of *Tlr2* mRNA in injured DRG. RBPs participate in RNA biogenesis by stabilizing RNA expression [33, 34, 35, 36]. To search for *cNrip1*‐associated RBPs, we used the biotinylated full‐length *cNrip1* and captured *cNrip1*‐binding proteins using the comprehensive identification of RNA‐binding proteins by mass spectrometry (ChIRP‐MS) assay. We found that *cNrip1* interacted with 28 proteins (the ratios of *cNrip1/Nrip1* mRNA ≥ 1.5) in cultured DRG neurons (Table S2). SYNCRIP is the most likely binding partner among these RNA‐binding proteins, as its binding motif—predicted using the RBPSuite webserver—was identified in the 3′ untranslated region (UTR) of *Tlr2* mRNA. EMSA demonstrated the interactions between SYNCRIP and *cNrip1* or *Tlr2* 3’‐UTR (Figure S16). The interaction between SYNCRIP and *cNrip1* was further validated by Western blot analysis using biotinylated full‐length *cNrip1* in cultured DRG neurons (Figure 10A). In addition, a fragment of *cNrip1* including the back‐splicing junction was immunoprecipitated by an anti‐SYNCRIP antibody, but not normal purified IgG, in the ipsilateral L4 DRG of sham mice (Figure 10B). SNL significantly increased this immunoprecipitating activity by 4.7‐fold of the value from the sham group in the ipsilateral L4 DRG on day 7 post‐surgery (Figure 10B). This increase was mainly attributed to the SNL‐induced increases in the levels of both *cNrip1* and SYNCRIP [34] in injured DRG.

We further carried out the RIP assay and found the binding of SYNCRIP to the 3’‐UTR of *Tlr2* mRNA in the ipsilateral L4 DRG of the AAV5‐scrambled shRNA‐microinjected mice on day 7 post‐sham surgery (Figure 10C). SNL markedly increased this binding in the AAV5‐scrambled shRNA‐microinjected mice (Figure 10C). However, this increase was absent in the AAV5‐*cNrip1* shRNA‐microinjected SNL mice (Figure 10C), suggesting that the increased binding of SYNCRIP to the 3’‐UTR of *Tlr2* mRNA is *cNrip1*‐dependent in injured DRG. Furthermore, when RNA transcription was halted with actinomycin D, the decay rate of *Tlr2* mRNA was much slower and the level of *Tlr2* mRNA was significantly elevated in the AAV5‐*Syncrip* (expressing the full‐length *Syncrip* mRNA) plus AAV5‐scrambled shRNA‐transduced cultured CAD cells compared with those in naïve cultured CAD cells (Figure 10D). These effects were attenuated in the AAV5‐*Syncrip* plus AAV5‐*Syncrip* shRNA‐ or AAV5‐*cNrip1* shRNA‐transduced cultured CAD cells (Figure 10D). Additionally, we conducted the luciferase reporter assay (in which the 3′‐UTR of *Tlr2* mRNA was cloned downstream of a luciferase reporter gene, and the resulting construct was used to assess luciferase reporter activity) and further verified the role of *cNrip1* in SYNCRIP‐trigged stability of *Tlr2* mRNA 3’‐UTR in the CAD cells. AAV5‐*Syncrip* transduction resulted in a significant increase in the luciferase reporter activity (Figure 10E), suggesting that SYNCRIP overexpression enhances the stability of *Tlr2* mRNA. This increase was markedly blocked in the presence of AAV5*‐Syncrip* shRNA or AAV5‐ *cNrip1* shRNA and enhanced in the presence of AAV5‐*cNrip1* (Figure 10E). AAV5‐*cNrip1* transduction alone led to a slight increase in the luciferase reporter activity. These in vitro data strongly suggest that SYNCRIP promotes the stability of *Tlr2* mRNA 3’‐UTR and that this effect requires *cNrip1*. This conclusion is further supported by evidence demonstrating the binding of *cNrip1* to the 3′‐UTR of *Tlr2* mRNA, as shown by EMSA (Figure S16) and further validated by chromatin isolation by RNA purification assay in cultured DRG neurons (Figure 10F). Our findings strongly indicate that *cNrip1*, unlike previously reported circRNAs associated with pain (acting as microRNA sponge) [37, 38], may act as an RNA adaptor/recruiter to recruit SYNCRIP to *Tlr2* mRNA 3’‐UTR, thereby stabilizing *Tlr2* mRNA expression in injured DRG.

Consistent with previous studies [34, 39], SNL increased the expression of SYNCRIP and TLR2 at both the mRNA and protein levels in the ipsilateral L4 DRG of the AAV5‐scrambled shRNA‐microinjected mice 14 days after surgery (Figure 10G,H). Pre‐microinjection of AAV5‐*Syncrip* shRNA into the ipsilateral L4 DRG not only blocked these increases (Figure 10G,H) but also attenuated the development of the SNL‐induced mechanical, heat and cold nociceptive hypersensitivities on the ipsilateral side (Figure S17A–D). This pre‐microinjection did not alter the basal levels of *Syncrip* mRNA, *Tlr2* mRNA and their corresponding proteins in the ipsilateral L4 DRG of sham mice (Figure 10G,H) and basal behavioral responses on the contralateral side of SNL mice and on either side of sham mice (Figure S17A–G). In addition, co‐microinjection of AAV5‐*cNrip1* and AAV5‐*Syncrip* shRNA into the unilateral L3/4 DRGs blocked the *cNrip1* overexpression‐induced increases of *Tlr2* mRNA and TLR2 protein in the microinjected DRGs and mechanical, thermal and cold nociceptive hypersensitivities on the ipsilateral side on week 5 post‐viral microinjection (Figure 10I,J; Figure S18A–D). Interestingly, AAV5‐*cNrip1* also increased the expression of *Syncrip* mRNA and SYNCRIP protein in the microinjected DRGs of the AAV5‐scrambled shRNA‐microinjected mice (Figure 10I,J). Given that *cNrip1* was co‐expressed with *Syncrip, Tlr2* and *Fus* mRNAs in the same individual small‐, medium‐ and large‐sized neurons of the ipsilateral L4 DRG on day 7 post‐SNL (Figure S9), our findings indicate that both upregulated *cNrip1* and increased SYNCRIP are required for nerve injury‐induced elevation of TLR2 in injured DRG neurons.

## 3 Discussion

Although nerve injury‐induced neuropathic pain has been extensively studied for decades, its underlying mechanisms remain elusive. In this study, we identified *cNrip1* and reported its upregulation triggered by increased FUS expression in injured DRG neurons following peripheral nerve injury. This upregulation contributes to the induction and maintenance of nerve injury‐induced neuropathic pain by promoting SYNCRIP‐triggered stabilization of *Tlr2* mRNA in injured DRG. *cNrip1* is likely an endogenous initiator of neuropathic pain.

**FIGURE 11:**
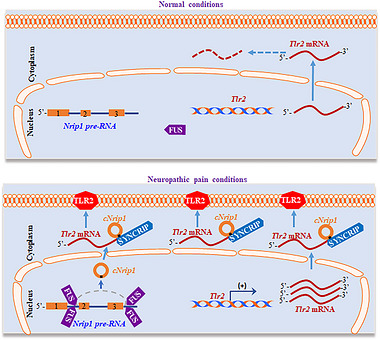
Model of *cNrip1* upregulation driving neuropathic pain mechanisms. Under normal conditions, the expression of *cNrip1*, FUS, TLR2 and SYNCRIP is relatively low in dorsal root ganglion (DRG) neurons. After peripheral nerve injury, increased FUS triggers the formation and upregulation of *cNrip1* in injured DRG neurons. Upregulated *cNrip1* recruits SYNCRIP to the 3′‐UTR of *Tlr2* mRNA by binding to both, thereby promoting SYNCRIP‐triggered *Tlr2* mRNA stability and increasing TLR2 expression on the neuronal membrane.

*cNrip1*, like circRNA‐Filip1l and circAnks1a in the spinal cord [37, 38], is time‐dependently upregulated in injured DRG neurons after peripheral nerve injury. Under normal conditions, the level of *cNrip1* was quite low in the DRGs of both mice and human. Its expression was significantly upregulated in the ipsilateral L4 DRG (but not intact ipsilateral L3 DRG, contralateral L4 DRG and ipsilateral L4 spinal cord) after SNL, ipsilateral L3/4 DRGs after CCI, and bilateral DRGs after PTX or STZ injection. Interestingly, basal level of *cNrip1* remained unchanged in the DRG following peripheral inflammation or incision. How DRG *cNrip1* upregulation is specific to neuropathic pain remains unknown, but the present study demonstrated that this upregulation may be due to nerve injury‐induced increase of FUS in injured DRG. FUS regulates gene expression through its binding to RNA, DNA and protein. Peripheral nerve injury increased the level of FUS in injured DRG [27, 28]. This increase contributed to nerve injury‐induced nociceptive hypersensitivity through activating NF‐kappa B pathway or triggering *Ccl2* promoter activation in primary sensory neurons [27, 28]. The present study further demonstrated that increased FUS participated in back‐splicing of *cNrip1* by binding to *Nrip1* pre‐RNA, thereby promoting *cNrip1* expression in injured DRG neurons (Figure 11). *cNrip1* may mediate the role of FUS in neuropathic pain. The precise mechanisms by which FUS preferentially promotes DRG *cNrip1* back‐splicing following nerve injury remain to be elucidated.

Unlike previous studies that used intrathecal injections to knock down circRNAs in both the DRG and spina cord [17, 18], the present study employed DRG AAV5 microinjection strategy and clearly demonstrated the role of DRG *cNrip1* in neuropathic pain. This microinjection procedure may induce minor tissue damage in the DRG, which could, in turn, potentially influence the outcomes of pain behaviors. However, our previous studies [30, 31, 32] and those of others [40, 41, 42, 43] demonstrated that the microinjected DRG, stained with cresyl‐violet or antibodies against inflammatory markers, retained its structural integrity, displayed no significant changes in the numbers of neurons and satellite cells and revealed no marked expression of inflammatory markers. About 60% of DRG neurons were transduced by AAV5‐*Gfp* 4 weeks after DRG microinjection [32]. GFP expressions is limited in microinjected DRG neuronal bodies and their peripheral afferents and efferent fibers on the injection side [32]. As expected, DRG microinjection with control AAV5‐*Gfp* or AAV5‐Scr shRNA did not alter basal sensitivity, as the microinjected animals displayed similar basal responses to mechanical, heat, and cold stimuli compared with baseline measurements prior to microinjection [30, 31, 32]. More importantly, DRG microinjection of AAV5‐*cNrip1* shRNA almost abolished the nerve injury‐induced increase of *cNrip1*, without affecting basal expression of *Nrip1* mRNA, in injured DRG. This is because *cNrip1* shRNA designed specifically targets the back‐splice junction, a sequence unique to the *cNrip1* and not present in the linear *Nrip1* mRNA. In addition, after blasting this sequence to mRNA/miRNA databases, it shows less than 5 contiguous nucleotides of sequence identity with other mRNAs or miRNAs. Unexpectedly, DRG microinjection of AAV5‐*cNrip1* shRNA failed to markedly knock down basal expression of *cNrip1* in sham DRG. Although the mechanism underlying the lack of effect on basal expression remains unclear, it is likely attributable to the low basal level of *cNrip1* under physiological conditions, which may not be further significantly reduced by AAV5‐*cNrip1* shRNA at the current injection titer and volume.

*cNrip1* promotes the SYNCRIP‐triggered stabilization of *Tlr2* mRNA in injured DRG. SYNCRIP, one of three alternative splicing variants (hnRNP Q1‐3), plays a major role in post‐transcriptional regulation of mRNAs, including mRNA stability [44]. SYNCRIP was reported to stabilize proto‐oncogene c‐fos mRNA in mammalian cultured cells [45] and to upregulate Prospero protein through binding to prospero mRNA 3′‐UTR to increase its stability [46]. SYNCRIP participated in neuropathic pain through stabilizing *Ccr2* mRNA expression in injured DRG [34]. The present study demonstrated that SYNCRIP contributed to nerve injury‐induced nociceptive hypersensitivity through its binding to *Tlr2* mRNA 3’‐UTR, thereby stabilizing *Tlr2* mRNA expression in injured DRG neurons. Furthermore, SYNCRIP‐triggered stabilization of *Tlr2* mRNA requires *cNrip1*. Blocking SNL‐induced upregulation of DRG *cNrip1* attenuated the SNL‐induced increase in the binding of SYNCRIP to *Tlr2* mRNA 3’‐UTR in injured DRG. Knockdown of *cNrip1* blocked the SYNCRIP‐induced delay of *Tlr2* mRNA degradation and the SYNCRIP‐induced increases of *Tlr2* mRNA 3’‐UTR stability. Given that *cNrip1* interacts with both SYNCRIP and *Tlr2* mRNA 3’‐UTR and co‐expresses with *Syncrip* and *Tlr2* mRNAs in individual DRG neurons, *cNrip1*, unlike earlier pain‐associated circRNAs (acting as microRNA sponge) [37, 38], may function as an RNA adaptor/recruiter to recruit SYNCRIP to *Tlr2* mRNA 3’‐UTR, thereby stabilizing *Tlr2* mRNA expression in DRG (Figure 11). In addition to SYNCRIP, the remaining 27 RBPs found by our ChIRP‐MS assay may also bind to *Tlr2* mRNA 3’‐UTR. Potential participation of *cNrip1* in these bindings and associated *Tlr2* mRNA stability in injured DRG cannot be excluded.

Upregulated *cNrip1* contributes to neuropathic pain through stabilizing *Tlr2* mRNA expression in injured DRG. TLR2 was expressed predominantly in small and medium DRG neurons [4]. Consistent with previous studies from TLR2 knockout mice [4, 8], blocking the SNL‐induced increase of TLR2 in injured DRG mitigated the SNL‐induced nociceptive hypersensitivity. Moreover, DRG overexpression of TLR2 in naïve mice produced enhanced responses to mechanical, heat and cold stimuli and spontaneous ongoing pain. These data indicate an important role of DRG increased TLR2 in neuropathic pain genesis. The present study demonstrated that upregulated *cNrip1* was responsible for TLR2 increase in injured DRG. Blocking the nerve injury‐induced upregulation of DRG *cNrip1* not only inhibited the nerve injury‐induced increases of *Tlr2* mRNA and TLR2 protein levels in injured DRG but also alleviated the development and maintenance of SNL‐induced nociceptive hypersensitivity. Conversely, mimicking the nerve injury‐induced upregulation of DRG *cNrip1* increased the levels of *Tlr2* mRNA and TLR2 protein in the DRGs, augmented responses to evoked noxious stimuli and led to spontaneous pain in naïve mice. Moreover, these nociceptive hypersensitivities were abolished after DRG knockdown of TLR2. In addition, knocking down of DRG SYNCRIP not only blocked the nerve injury‐induced increases of *Tlr2* mRNA and TLR2 protein levels in injured DRG but also attenuated *cNrip1* overexpression‐induced increase of *Tlr2* mRNA and TLR2 protein in the DRG and *cNrip1* overexpression‐induced nociceptive hypersensitivity. These findings further imply that both *cNrip1* and SYNCRIP are required for nerve injury‐induced increase of TLR2 expression in injured DRG neurons (Figure 11). It is worth noting that DNA demethylation, histone acetylation, and microRNA downregulation (e.g., miR‐146a, miR‐155) may also participate in DRG *Tlr2* mRNA increase under neuropathic pain conditions [47, 48]. Future studies are needed to determine whether these mechanisms interact with *cNrip1*/SYNCRIP to regulate the nerve injury–induced increase of TLR2 in injured DRG.

Several lines of evidence support the role of small, medium and large DRG neurons in neuropathic pain [49, 50, 51, 52, 53, 54, 55, 56]. Both *cNrip1* and TLR2 were significantly increased in DRG neurons, particularly in medium DRG neurons [4, 5]. TLR2 is considered an endogenous initiator of neuropathic pain likely by indirectly elevating expression of ion channels and receptors (e.g., Nav1.8 and TRPV1), lowering activation threshold of nociceptors and increasing spontaneous activity of DRG neurons [4, 57]. We speculate that upregulated *cNrip1* contributes to neuropathic pain through the SYNCRIP‐triggered stability of *Tlr2* mRNA expression, thereby increasing TLR2 expression in injured DRG neurons. The increased TLR2 triggers MyD88/NF‐κB signaling pathway and/or leads to the local production of inflammatory mediators (e.g., CCL2, IL6, IL‐1β and TNF‐α) [4, 57, 58]. These downstream signal molecules increase DRG neuronal excitability [59, 60, 61, 62, 63] and consequent elevation of neurotransmitter release from primary afferents and central sensitization of spinal cord dorsal horn. Indeed, the present study showed that blocking the nerve injury‐induced upregulation of DRG *cNrip1* diminished the nerve injury‐induced hyperactivity of dorsal horn neurons and astrocytes. Thus, upregulated *cNrip1* expressed in injured small, medium and large DRG neurons contributes to neuropathic pain through SYNCRIP‐triggered stability of DRG TLR2. It should be noted that TLR2 is also expressed in satellite cells of DRG [57] and increased in spinal cord microglia. The role of TLR2 in these glial cells under neuropathic pain conditions cannot be ruled out.

In conclusion, we identified a *cNrip1*‐mediated mechanism by which SYNCRIP stabilizes *Tlr2* mRNA expression in injured DRG neurons under neuropathic pain conditions. Notably, *cNrip1* was selectively upregulated in injured DRG, and blocking this upregulation attenuated both development and maintenance of nerve injury‐induced nociceptive hypersensitivity—without affecting acute or basal pain responses or locomotor function. These findings suggest that *cNrip1* may serve as a promising therapeutic target for the treatment of neuropathic pain. However, more attention to unwanted potential side effects should be paid, because *cNrip1* is expressed in other body tissues. In addition, although *cNrip1* is identified in human DRG, whether *cNrip1* promotes SYNCRIP‐triggered *Tlr2* mRNA stability in human DRG neurons remains to be validated.

## 4 Experimental Section

### 4.1 Animals

Adult male and female CD‐1 mice weighing 25–30 g were used. All mice were kept at 24°C and 50%–60% humidity under a standard 12 h light/dark cycle, with water and food pellets available ad libitum in the central housing facility at Rutgers New Jersey Medical School. All experimental procedures were approved by the Rutgers New Jersey Medical School Animal Care Committee (PROTO202500070) and were conducted according to the guidelines of the US National Institutes of Health and the International Association for the Study of Pain on animal care and with ethical guidelines. The researchers were blinded to animal behavior tests after the treatment.

### 4.2 Animal Models

For nerve trauma‐induced neuropathic pain models, L4 spinal nerve ligation (SNL) and sciatic nerve chronic constriction injury (CCI) in mice were conducted as published previously [27, 31, 53, 64]. All animals were anesthetized with isoflurane. For the SNL model, an incision on the lower back was made. After removal of the L4 transverse process, unilateral L4 spinal nerve was exposed and then ligated with 7–0 silk thread with transection at the distal site. For the CCI model, unilateral sciatic nerve was exposed and loosely ligated with 7‐0 silk thread at four sites at intervals of about 1 mm proximal to trifurcation of the sciatic nerve. Sham animals received the same surgery but without the ligation and/or transection of the respective nerve. For chemotherapy‐induced neuropathic pain model, PTX (4 mg/kg; dissolved in the vehicle) or vehicle (50% Cremophor EL (Sigma‐Aldrich) and 50% ethanol (Sigma‐Aldrich)) was injected intraperitoneally (i.p.) every other day for a total of four injections (day 0, 2, 4, and 6) as described previously [65, 66, 67]. For STZ‐induced diabetic neuropathic pain model, STZ (165 mg/kg; dissolved in the vehicle) or vehicle (10 mM citrate buffer, pH 4.4, Sigma‐Aldrich) was injected i.p. as described [67]. 3 days after STZ or vehicle injection, blood glucose levels were measured in mice fasted for 12 h. Only mice with blood glucose levels greater than 15 mmol/L were included in the study. For chronic inflammatory pain model in mice, 20 µL of complete Freund's adjuvant (CFA, Sigma‐Aldrich) was injected subcutaneously into the plantar surface of one hind paw or 5 µL of mono‐iodoacetate (MIA, Millipore Sigma) at a concentration of 50 µg/µL (diluted in 0.9% saline) was injected into the unilateral knee joint as described previously [27, 53]. For incisional pain model in mice, a 0.8‐cm longitudinal incision was made through the skin, fascia, and muscle of the unilateral plantar aspect of the hind paw as described previously [68, 69].

### 4.3 Behavioral Tests

Mice were habituated for 1 to 2 h every day for 2 to 3 days before basal behavioral testing. The evoked behavioral testing, including mechanical, heat, and cold tests were carried out in sequential order at 1‐h intervals. Conditional place preference (CPP) testing was performed 7 days after surgery or 6 or 7 weeks after viral microinjection. Locomotor function testing was carried out after pain behavioral tests were done and before the tissue collection.

Paw withdrawal thresholds in response to mechanical stimuli were measured with two calibrated von Frey filaments (0.07 and 0.4 g, Stoelting Co., Wood Dale, IL) [65, 66, 67]. Briefly, mice were placed in a Plexiglas chamber on an elevated mesh screen and allowed to habituate for 30 min. Animals received 10 repeated applications (5 min apart) of each von Frey filament to the middle of the plantar surface of each hind paw. A quick withdrawal of the paw was regarded as a positive response. The number of positive responses among 10 applications was recorded as a percentage withdrawal frequency.

Paw withdrawal latencies in response to noxious heat stimulation were examined with a Model 336 Analgesia Meter (IITC Inc. Life Science Instruments. Woodland Hills, CA) [65, 66, 67]. Briefly, mice were placed in a Plexiglas chamber on a glass plate. A beam of light was emitted from a hole in the lightbox was applied to the middle of the plantar surface of each hind paw. The light beam was automatically turned off when the hind paw was quickly lifted. The length of time between the start and the stop of the light beam was defined as the paw withdrawal latency. For each side, 3–4 trials at 5 min intervals were carried out. A cutoff time of 20 sec was used to avoid tissue damage to the hind paw.

Paw withdrawal latencies to noxious cold (0°C) were examined with a cold aluminum plate, which temperature was monitored continuously by a thermometer [65, 66, 67]. The paw withdrawal latency was recorded as the length of time between placement on the plate and the first sign of the mouse jumping and/or flinching. Each test was repeated three times at 10 min intervals. To avoid tissue damage, a cut‐off time of 20 sec was used.

CPP test was conducted in an apparatus with two Plexiglas chambers connected with an internal door (Med Associates Inc., St. Albans, VT) [65, 66, 67]. One of the chambers has a rough floor and walls with horizontal black and white stripes, whereas the other contained a smooth floor and walls with vertical black and white stripes. Movement of the mice and time spent in each chamber were monitored by photobeam detectors installed along the chamber walls and automatically recorded in MED‐PC IV CPP software. Mice were first preconditioned with full access to both chambers to habituate them to the environment for 30 min. At the end of the preconditioning phase, basal duration spent in each chamber was recorded within 15 min to check whether mice had a preexisting chamber bias. The conditioning protocol was performed for the following 3 days with the internal door closed. The mice first received an intrathecal injection of saline (5 µL) specifically paired with one conditioning chamber in the morning. 6 h later, lidocaine (0.8% in 5 µL of saline) was given intrathecally paired with another opposite conditioning chamber. The injection order of saline and lidocaine was switched every day. On the test day, the mice were placed in one chamber with free access to both chambers. The duration of time that each mouse spent in each chamber was recorded for 15 min. Difference scores were calculated by subtracting preconditioning time from test time spent in the lidocaine chamber.

Locomotor function including placing, grasping, and righting reflexes, was examined as described [65, 66, 67]. For the placing reflex, the positions of the hind limbs were slightly lower than those of the forelimbs, and the dorsal surfaces of the hind paws were brought into contact with the edge of a table. Whether the hind paws were placed on the table surface reflexively was recorded. For the grasping reflex, after the mouse was placed on a wire grid, whether the hind paws grasped the wire on contact was recorded. For the righting reflex, when the mice were placed on their backs on a flat surface, whether it immediately assumed the normal upright position was recorded. Each trial was repeated five times at 5 min intervals and the scores for each reflex were recorded based on counts of each normal reflex.

### 4.4 DRG Microinjection

DRG microinjection was carried out with minor modification as described previously [65, 66, 67]. Briefly, after the mouse was anesthetized with isoflurane, a 3‐cm long skin incision was made aseptically at the midline of the lower back. The unilateral L4 and/or L3 articular processes were exposed and then removed. Viral solution (1 µL/DRG, titer ≥ 3 × 10^12^/µL) was carefully injected into the exposed ipsilateral L4 or L3/4 DRGs with the use of a glass micropipette connected to a Hamilton syringe. The micropipette was removed 10 min after the injection. The wound was copiously irrigated with sterile saline and the skin was closed with wound clips.

### 4.5 Cell Culture and Transfection

HEK‐293T or CAD cell cultures and DRG neuronal cultures were prepared as previously described [27, 31, 53, 64]. Briefly, HEK‐293T cells were cultured in Dulbecco's modified Eagle's medium/high glucose medium (Gibco/Thermo Fisher Scientific) containing 10% fetal bovine serum (FBS) and 100 units/ml Penicillin and 100 µg/ml Streptomycin (Quality Biological, Gaithersburg, MD). CAD cells were cultured in Dulbecco's modified Eagle's medium/F‐12 HEPES (Gibco/Thermo Fisher Scientific) containing 8% fetal bovine serum (FBS) and 100 units/ml Penicillin and 100 µg/ml Streptomycin (Quality Biological, Gaithersburg, MD). For primary DRG neuronal cultures, DRGs from the mice (≥ 4 weeks) were collected in cold Neurobasal Medium (Gibco/ThermoFisher Scientific) containing 10% fetal bovine serum (JR Scientific, Woodland, CA), 5 mL L‐glutamine (200 mM) (Gibco/ThermoFisher Scientific), 10 mL B‐27 Supplement (50×) (Gibco/ThermoFisher Scientific), 100 units/ml Penicillin and 100 µg/ml Streptomycin (Quality Biological, Gaithersburg, MD). The DRGs were then treated with enzyme solutions (5 mg/ml dispase, 1 mg/ml collagenase type I) in Hanks’ balanced salt solution (HBSS) without Ca2^+^ and Mg2^+^ (Gibco/ThermoFisher Scientific). After trituration and centrifugation, dissociated cells were resuspended in mixed Neurobasal Medium and plated in a six‐well plate coated with 50 µg/ml poly‐D‐lysine (Sigma, St. Louis, MO) at 1.5–4 × 10^5^ cells. The cells were incubated at 5% CO_2_ and 37°C. On the second day, 4–10 µL of virus (titer ≥ 6–9 × 10^12^/µL) were added to each 2 mL well. Cells were harvested 3 days later.

### 4.6 Quantitative Real‐Time Reverse Transcription (RT)‐PCR Assay

Total RNA from mouse tissues/cells was extracted and purified using the RNeasy mini kit (Qiagen) and treated with excess DNase I (New England Biolabs, Ipswich, MA). Total DNase‐treated RNA from human DRGs was purchased from Clontech Laboratories, Inc. RNA concentration was measured and spectrophotometric ratios of A260/280 nm were between 1.97 and 2.08. cDNA was synthesized by using the Omniscript RT Kit (Qiagen) with specific RT primers or oligo(dT) primers. Quantitative real‐time PCR assays were conducted using SYBR Green real‐time PCR Master Mix. Reactions were carried out in a BIO‐RAD CFX96 real‐time PCR system. Ratios of RNA levels at different time points post‐surgery to basal RNA level (0 day) or ratios of RNA levels in other treated groups to RNA level in the control group were calculated using the ΔCt method (2^−ΔΔCt^). Negative control reactions, which included primers and all other reagents but no template, showed no detectable amplification. The primers used are listed in the Table S2. The melting curve analysis for all qPCR primers used are present in Figure S19 to validate their specificity.

### 4.7 Single‐Cell RT‐PCR Assay

The freshly cultured mouse DRG neurons were first prepared. 4 h after plating, under an inverted microscope fit with a micromanipulator and microinjector, single living large (> 35 µm), medium (25–35 µm), or small (< 25 µm) DRG neurons were collected in a PCR tube with 6–8 µL of cell lysis buffer (Signosis). After centrifugation, the supernatants were collected and divided into PCR tubes for different genes. The remaining RT‐PCR procedure was performed according to the manufacturer's instructions with the Single‐Cell RT‐PCR Assay Kit (Signosis). All nest‐PCR primers used are listed in the Table S2.

### 4.8 Plasmid Constructs and Virus Production

Full‐length *Tlr2, Syncrip, cNrip1* or *Fus* cDNAs were respectively amplified from mouse DRG RNA with the Platinum Taq High Fidelity Kit (Invitrogen/Thermo‐Fisher Scientific) and primers with restriction enzymes (Table S2). After double enzyme digestion, the PCR products were inserted into the corresponding sites of the specific pAAV‐CMV‐circ Expression Vector [70] (for *cNrip*) or regular pHpa‐trs‐SK plasmids (for *Tlr2*, *Syncript* and *Fus*) to replace enhanced GFP sequence at the multiple cloning sites (Cell Biolabs, CA). The resulting vectors expressed the genes under the control of the cytomegalovirus promoter. The designed sense and antisense sequences for *Tlr2*, *Syncrip, cNrip1* and *Fus* shRNA were annealed and inserted between the *Bam HI* and *Xba I* sites of pAAV‐shRNA‐EF1a‐EYFP. In particular, *cNrip1* shRNA was designed to specifically target the back‐splice junction of *cNrip1*, a sequence unique to the *cNrip1* and not present in other mRNAs (including *Nrip1* mRNA) and miRNAs. AAV5 packaging of viral particles carrying the cDNA was carried out using the AAVpro Purification Kit (Takara, Mountain View, CA). The virus titer was evaluated using the AAVpro Titration Kit (Takara).

### 4.9 RNA Sequencing

On day 7 post‐SNL or sham surgery in mice pre‐microinjected with AAV5‐*Gfp* or AAV5‐*cNrip1* into the ipsilateral L4 DRGs for 35 days, the ipsilateral L4 DRGs were harvested and pooled to achieve enough RNA. Total RNA (1.5 µg/sample) extracted was subjected to rRNA depletion by Ribo‐Zero rRNA Removal (Human/Mouse/Rat) Kit (Illumina, San Diego, CA, USA). Strand‐specific RNA libraries were prepared using TruSeq Stranded Total RNA Sample Preparation Kit (Illumina) without poly‐A selection. Sequencing was carried out using the Illumina HiSeq2500 platform High Output Mode, in a 2 × 150 bp paired‐end configuration, with a total of more than 190 M reads per lane (at least 60 M reads per sample)

### 4.10 Northern Blotting Assay

Northern blot analysis was performed as previously described [27, 53]. Briefly, digoxigenin‐dUTP labelled complementary RNA (cRNA) probe of mouse *cNrip1* (including junction site) were generated by PCR using genomic DNA from mouse as the template. RNA (10 µg) extracted from the ipsilateral L4 DRG 7 days after SNL was treated with or without an overdose of RNase R, then separated on a 1.5% agarose‐formaldehyde gel, transferred to BrightStar‐plus positively charged nylon membrane (AM10100, Ambition) and cross‐linked using UV light (150 mJ/cm^2^). The membrane was hybridized with the probe at 68°C overnight. On next day, the membrane was washed in a low salt buffer at room temperature for 2 × 5 min, high salt buffer at 68°C for 2 × 5 min and SSC at 68°C for 1 × 2 min. After being blocked, the membrane was incubated with alkaline phosphatase‐conjugated sheep anti‐digoxigenin (1:500, Roche) for 1 h at room temperature, and washed for 2 × 5 min, incubated by CDP‐Star solution provided in the DIG Northern Starter Kit (Roche) and imaged by the ChemiDoc XRS System with Image Lab software (Bio‐Rad). To clearly display the markers, the membranes loaded with the markers were separately imaged.

### 4.11 Luciferase Reporter Assay

A 148‐bp fragment from the 3’‐UTR region of *Tlr2* was amplified by PCR from genomic DNA using the primers (Table S2) to construct the gene reporter plasmid as described previously [27, 53]. The PCR products were cloned into the *Kpn I* and *Nhe I* restriction sites of psiCheck vector (Promega, Madison, WI). The accuracy of recombinant clones was verified by DNA sequencing. CAD cells were plated on a 12‐well plate and cultured at 37°C in a humidified incubator with 5% CO_2_. Next day after culture, the cells in each well were co‐transfected with 300 ng of plasmid expressing full‐length *cNrip1*, *Syncrip* shRNA, or *cNrip1* shRNA, 300 ng of pGL4‐Basic vector (reporter vector) with or without the 3’‐UTR region of *Tlr2* sequence, and 10 ng of the pRL‐TK (Promega) using Lipofectamine 3000 (Invitrogen), according to the manufacturer's instructions. 2 days after transfection, the cells were collected and lysed in passive lysis buffer. Approximately 10 µL of supernatant was used to measure the luciferase activity using the Dual‐Luciferase Reporter Assay System (Promega). Transfection experiments were repeated three independent times. Relative reporter activity was calculated after normalization of the firefly activity to renilla.

### 4.12 BaseScope In Situ Hybridization and Co‐Immunohistochemistry Assays

Mice were deeply anaesthetized with isoflurane and transcranial perfused with ice‐cold 4% paraformaldehyde in 0.1 M PBS. Following perfusion, bilateral L4 DRG was harvested, post‐fixed for 4–6 h at 4°C in the same fixative and cryoprotected in 30% sucrose overnight. A series of 15‐µm frozen transverse sections were cut on a cryostat (Leica). The BaseScope i*n situ* hybridization (ISH) was carried out by using a protocol tailored to the BaseScope 2.5 HD Detection reagent‐red kit (REF:322360, ACD) with minor modification. The positive control probe for the housekeeping gene Ppib (REF: 320881, ACD) and the negative control probe for the bacterial gene DapB were used to validate the ISH procedure. Briefly, after the sections were treated with 0.3% Triton‐X‐100 in PBS at room temperature (RT) for 30 min, hydrogen peroxide was applied for 10 min at RT, then treated with Protease Plus (REF: 32330, ACD) for 15 min at 40°C, and incubated with the probe directed against mouse *cNrip1* (REF: 713121, ACD) at 62°C over two nights. The signals were revealed following the manufacturer's instructions as follows: AMP 1 for 30 min at 40°C, AMP 2 for 15 min at 40°C, AMP 3 for 30 min at 40°C, AMP 4 for 15 min at 40°C, AMP 5 for 30 min at RT and AMP 6 for 15 min at RT. The sections were washed with wash buffer three times at RT after each step. The signals were revealed by using BASEScope Fast RED assay following the manufacturer's instructions. Following ISH, the immunohistochemistry staining was performed as described previously [27, 53]. After being blocked for 1 h at RT in 0.01 M PBS containing 0.3% Triton X‐100 plus 4% goat serum, the sections were incubated with chicken anti‐β‐tubulin III (1:200, EMD Millipore), rabbit anti‐glutamine synthetase (1:500, Sigma‐Aldrich), rabbit anti‐CD68 (1:100, Abcam) or rabbit anti‐ATF3 (1:100, Abcam) at 4°C overnight, respectively. The fluorescent signals were developed with appropriate fluorescence‐conjugated secondary antibodies. The images were captured with a Leica DMI4000 fluorescence microscope (Leica). Immunoreactive neurons containing three or more particles of *cNrip1* were considered ′co‐expressed' cells, as defined by preceding studies [27, 53].

### 4.13 Bioinformatics Analysis

For identification and quantification of circRNAs, the reads obtained from the sequencing were first filtered to obtain high‐quality clean reads. To obtain effective clean reads, the residual rRNA was mapped and removed using an RNA central database. The remaining reads were used for alignment and analysis. These reads were aligned to mouse reference genome using STAR software [71], and their back‐splice junction reads were identified. Differential expression analysis of the RNA‐seq was performed using DESeq in R with the default parameters. The genomic location and length information of the candidate circRNAs were annotated. The potential binding sites of *cNrip1* to the 3’‐UTR of *Tlr2* mRNA and of FUS to the *Nrip1* pre‐mRNA were predicted by five bioinformatics software programs (IntaRNA, LncRNA, TargetScan, RBPSuite and LncTar).

### 4.14 Western Blotting Assay

The fresh tissues or cells were homogenized and lysed in ice‐cold lysis buffer (10 mM Tris, 1 mM phenylmethylsulfonyl fluoride, 5 mM MgCl2, 5 mM EGTA, 1 mM EDTA, 1 mM DTT, 40 µM leupeptin, 250 mM sucrose) containing proteinase inhibitor and phosphatase inhibitor. After the protein concentration was measured using the Bio‐Rad protein assay (Bio‐Rad), the samples were heated at 99°C for 5 min and loaded onto a 4%–15% stacking/7.5% separating SDS‐polyacrylamide gel (Bio‐Rad Laboratories). The proteins were then electrophoretically transferred onto a polyvinylidene difluoride membrane (Bio‐Rad Laboratories). The membranes were blocked with 3% non‐fat milk plus 0.1% Tween‐20 in Tris‐buffered saline for 1 h and incubated over night at 4°C with the following primary antibodies including rabbit anti‐TLR2 (1:500, Abcam), mouse anti‐SYNCRIP (1:200, Santa Cruz), rabbit anti‐FUS (1:1,000, Abcam), rabbit anti‐phospho‐ERK1/2 (Thr202/Tyr204, 1:800, Cell Signaling), rabbit anti‐ERK1/2 (1:800, Cell Signaling), mouse anti‐GFAP (1:800, Cell Signaling), rabbit anti‐GAPDH (1:2,000, Santa Cruz), and rabbit anti‐histone H3 (1:1,000, Cell Signaling). The proteins were detected by western peroxide reagent and luminol/enhancer reagent (Clarity Western ECL Substrate, Bio‐Rad) and exposed using the ChemiDoc XRS System with Image Lab software (Bio‐Rad). The intensity of blots was quantified with densitometry by using NIH Image J Software. GAPDH and histone H3 were used as loading controls for cytoplasmic and nuclear protein, respectively. Both expressions are not altered in the DRG following peripheral nerve injury [27, 30, 31, 32, 53, 64].

### 4.15 Comprehensive Identification of RNA‐Binding Proteins by Mass Spectrometry Assay

The Pierce Magnetic RNA‐Protein Pull‐Down Kit (Thermo Fisher) was used according to the manufacturer's instructions. The biotinylated full‐length sense *cNrip1* RNA (2 µg) as well as sense linear *Nrip1* RNA (2 µg, used as a negative control) were synthesized by Biotin RNA Labeling Mix (Roche) and purified by the Thermo GeneJET RNA Purification Kit (Thermo Fisher). These sense RNAs were incubated, respectively, with the DRG lysates overnight. The RNA/protein complex was pulled down using Dynabeads MyOne Streptavidin T1 (Invitrogen). The collected proteins were solubilized in Laemmli sample buffer and separated by the 4%–20% Mini‐PROTEAN TGX Precast Protein Gels (Bio‐Rad). Mass spectrometry experiments and data analysis were carried out in the Center for Advanced Proteomics Research, Rutgers New Jersey Medical School. To verify the binding of *cNrip1* to SYNCRIP, Western blot assay was carried out as described above.

### 4.16 RNA Immunoprecipitation (RIP) Assay

The Magna RIP Kit (Upstate/ EMD Millipore, Darmstadt, Germany) was used according to the manufacturer's instructions. Briefly, the DRGs collected were homogenized in the RIP lysis buffer containing the protease inhibitor cocktail and RNase inhibitor. The Magnetic Beads Protein A/G suspension for each IP was washed twice with the RIP wash buffer. Mouse anti‐SYCRIP antibody (2.0 µg; Abcam) or purified mouse IgG was incubated with the Magnetic Beads Protein A/G re‐suspended in RIP wash buffer for 30 min at room temperature. The DRG lysate was incubated with beads‐antibody complex in the RIP immunoprecipitation buffer overnight at 4°C by rotating. After the samples were washed six times with the RIP wash buffer, RNA was eluted from the beads by incubating in the proteinase K buffer at 55°C for 30 min by shaking, purified by phenol/chloroform extraction, and analyzed by quantitative RT‐PCR as described above. The 10% supernatant of the DRG lysate was used as Input. All primers used are listed in Table S3.

### 4.17 Chromatin Isolation by RNA Purification

Briefly, the cultured DRG neurons were prepared as described above. After the cultured neurons were rinsed with chilled PBS, 1% formaldehyde was added for crosslinking for 10 min. The reactions were quenched by adding 0.125 M glycine for 5 min. Cell pellets were collected and dissolved in nuclear lysis buffer. The lysates were sonicated to break DNA into 100–300 bp fragments. The biotinylated antisense RNA probe (1.5 µg) that was complementary to *cNrip1* sequence was designed using an online tool (Singlemoleculefish.com). The corresponding sense RNA probe (1.5 µg) was used as a negative control. The probes were incubated with the cell lysates overnight. The probes/RNA complex were pulled down using streptavidin magnetic C1 beads (Invitrogen). RNA was eluted, purified and analyzed by quantitative RT‐PCR as described above. The 10% supernatant of the cell lysate was used as Input.

### 4.18 Electrophoretic Mobility Shift Assay

The direct recruitment of SYNCRIP to the *Tlr2* 3′‐UTR by *cNrip1* and the interaction between FUS and *pre‐Nrip1* mRNA were assessed by electrophoretic mobility shift assay (EMSA) using the LightShift Chemiluminescent EMSA Kit (Pierce, 20148) according to the manufacturer's instructions. Briefly, FUS protein (6 µg; BOC Sciences, NY, USA) or SYNCRIP protein (6 µg; Bon‐Opus Biosciences, NJ, USA) was incubated at room temperature for 20 min in a 20 µL reaction containing 2 µL binding buffer, 1 µL glycerol, 1 µL poly(dI·dC), and biotin‐labeled RNA probe (30 ng for *Nrip1 pre‐mRNA* intron 1 or 20 ng for *cNrip1* or *Tlr2* 3′‐UTR). After the addition of 5 µL of 5× loading buffer, samples were resolved on a 6% non‐denaturing polyacrylamide gel in 0.5× TBE at 4°C. For competition assays, a 50‐fold excess of unlabeled wild‐type or mutant *Nrip1 pre‐RNA* intron 1, *cNrip1*, or *Tlr2* 3′‐UTR was added prior to incubation with the biotin‐labeled probe. Following electrophoresis, RNA–protein complexes were transferred onto a PVDF membrane and detected using streptavidin‐HRP and a chemiluminescent substrate.

### 4.19 Statistical Analysis

For in vivo experiments, mice were randomly distributed across experimental cohorts. For in vitro experiments, the cells were evenly suspended and then randomly distributed into each well tested. The sample sizes were determined based on our pilot studies, previous reports in the field and power analyses (power of 0.90 at *p* < 0.05). All results are shown as the means ± standard error of the mean (SEM) of at least three independent experiments. Data distribution was assumed to be normal, but this was not formally tested. The data were statistically analyzed using two‐tailed, paired Student's t‐test and a one‐way, two‐way, or three‐way ANOVA. When ANOVA showed a significant difference, pairwise comparison between means was performed using the post hoc Tukey method (SigmaPlot 12.5, San Jose, CA). Significance was set at *p* < 0.05.

## Conflicts of Interest

The authors declare no conflict of interest.

## Supporting information




**Supporting File**: advs75196‐sup‐0001‐SuppMat.pdf.

## Data Availability

The data that support the findings of this study are available from the corresponding author upon reasonable request.
