# Supplementary material for: Circular RNA circNrip1 Interacts with SYNCRIP to Promote Neuropathic Pain by Stabilizing Tlr2 mRNA in Primary Sensory Neurons
Source: Adv Sci (Weinh). 2026 Apr 9:e19740. Online ahead of print. doi: 10.1002/advs.202519740 (PMC13334684; doi:10.1002/advs.202519740)
Supplement: Supplementary file 1 — Supporting File: advs75196‐sup‐0001‐SuppMat.pdf. [file ADVS-9999-e19740-s001.pdf]

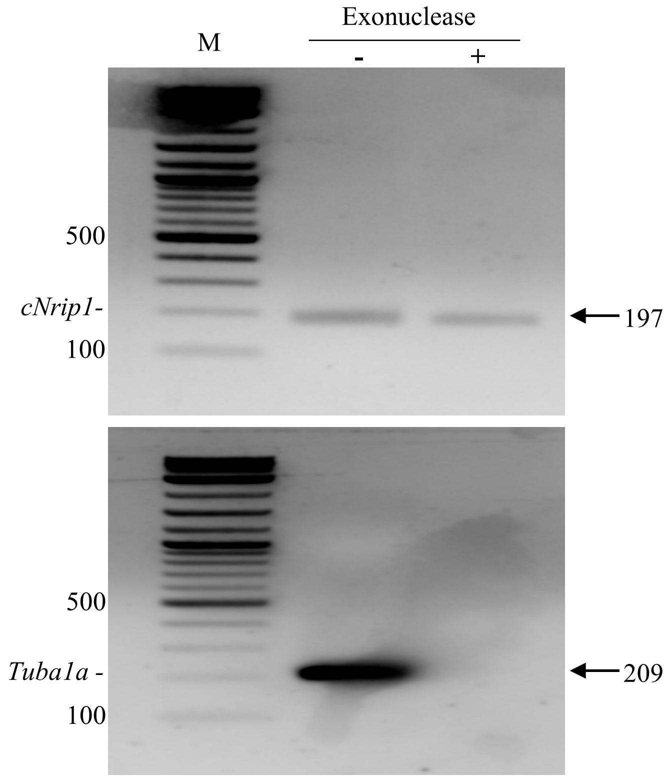

Supplementary Figure 1. *cNrip1* expression in the lumbar DRGs from naive mice treated with or without exonuclease using RT-PCR assay with strand-specific primers. Full-length *cNrip1* was detected as the expected size. Linear *Tuba1a* mRNA was used as a negative control. M: DNA ladder marker.

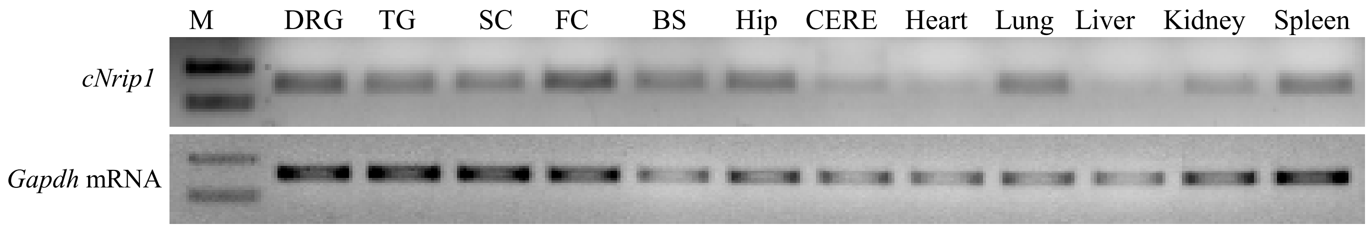

Supplementary Figure 2. Expression of *cNrip1* in different tissues in normal mice. DRG: dorsal root ganglion. TG: trigeminal ganglia. SC: spinal cord. FC: frontal cortex. BS: brain stem. Hip: hippocampus. Cere: cerebellum. *Gapdh* mRNA is used as an internal control.

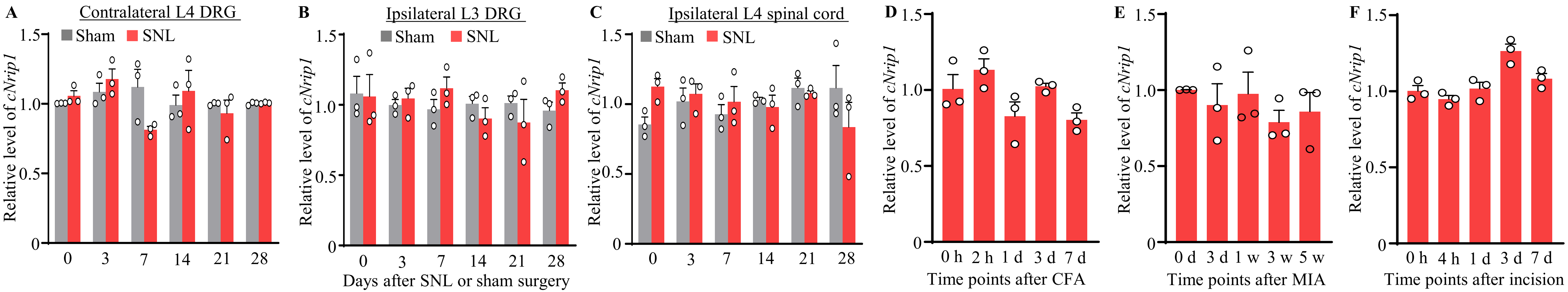

Supplementary Figure 3. Expression of *cNrip1* is not significantly changed under the following conditions: (A-C) Levels of *cNrip1* in the contralateral L4 DRG (A), ipsilateral L3 DRG (B), and ipsilateral L4 spinal cord dorsal horn (C) on days as indicated after SNL or sham surgery. *n* = repeats (3-12 mice)/time point/group. Two-way ANOVA followed by post hoc Tukey's test. (D-F) Levels of *cNrip1* in the ipsilateral L3/4 DRGs after unilateral hind-paw injection of complete Freund's adjuvant (CFA) (D), after unilateral intra-articular injection of sodium monoiodoacetate (MIA) (E), or after unilateral hind-paw incision (F). *n* = 3 repeats (6 mice)/time point/group. One-way ANOVA followed by post hoc Tukey's test.

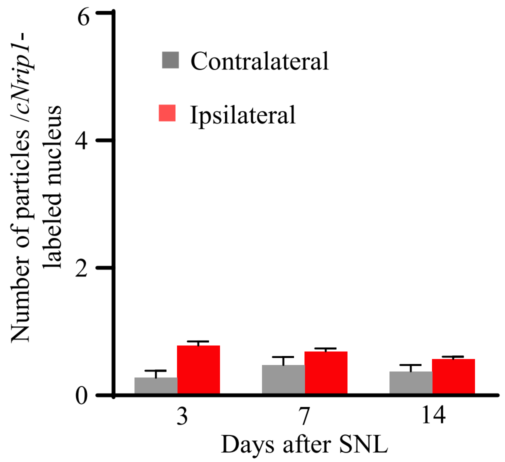

Supplementary Figure 4. Number particles per *cNrip1*-labled nucleus in the ipsilateral and contralateral L4 DRG on days 3, 7, and 14 after SNL. *n* = 5 mice/time point. 2-way ANOVA followed by post hoc Tukey's test.

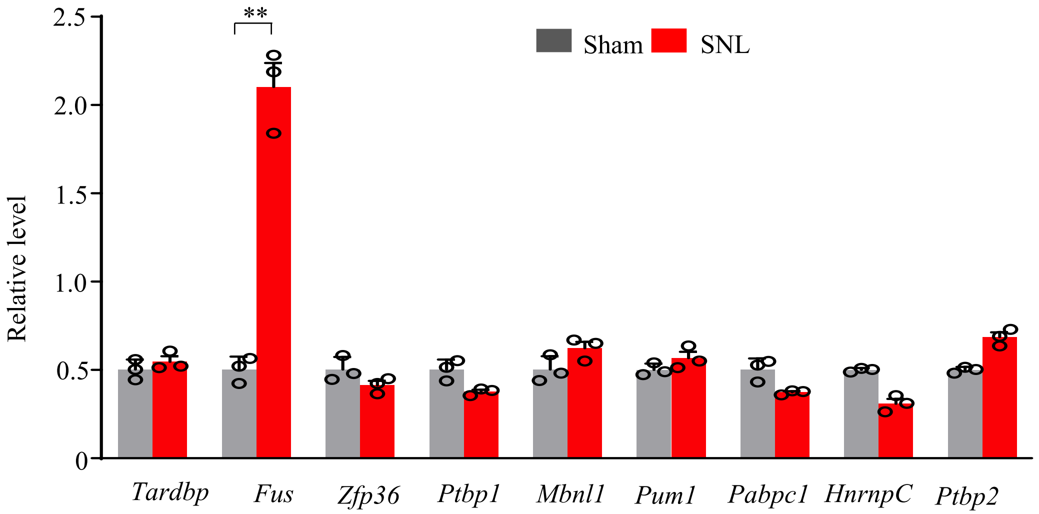

Supplementary Figure 5. Level of *Tardbp, Fus, Zfp36, Ptbp1, Mbnl1, Pum1, Pabpc1, HnrnpC* and *Ptbp2* mRNAs in the ipsilateral L4 DRG after SNL or sham surgery. These RNA-binding proteins are predicted to potentially bind to *Nrip1* pre-RNA by RBPSuite webserver. $n = 3$ repeats (12 mice)/group. $^{**}P < 0.01$ by two-tailed, unpaired Student's t test.

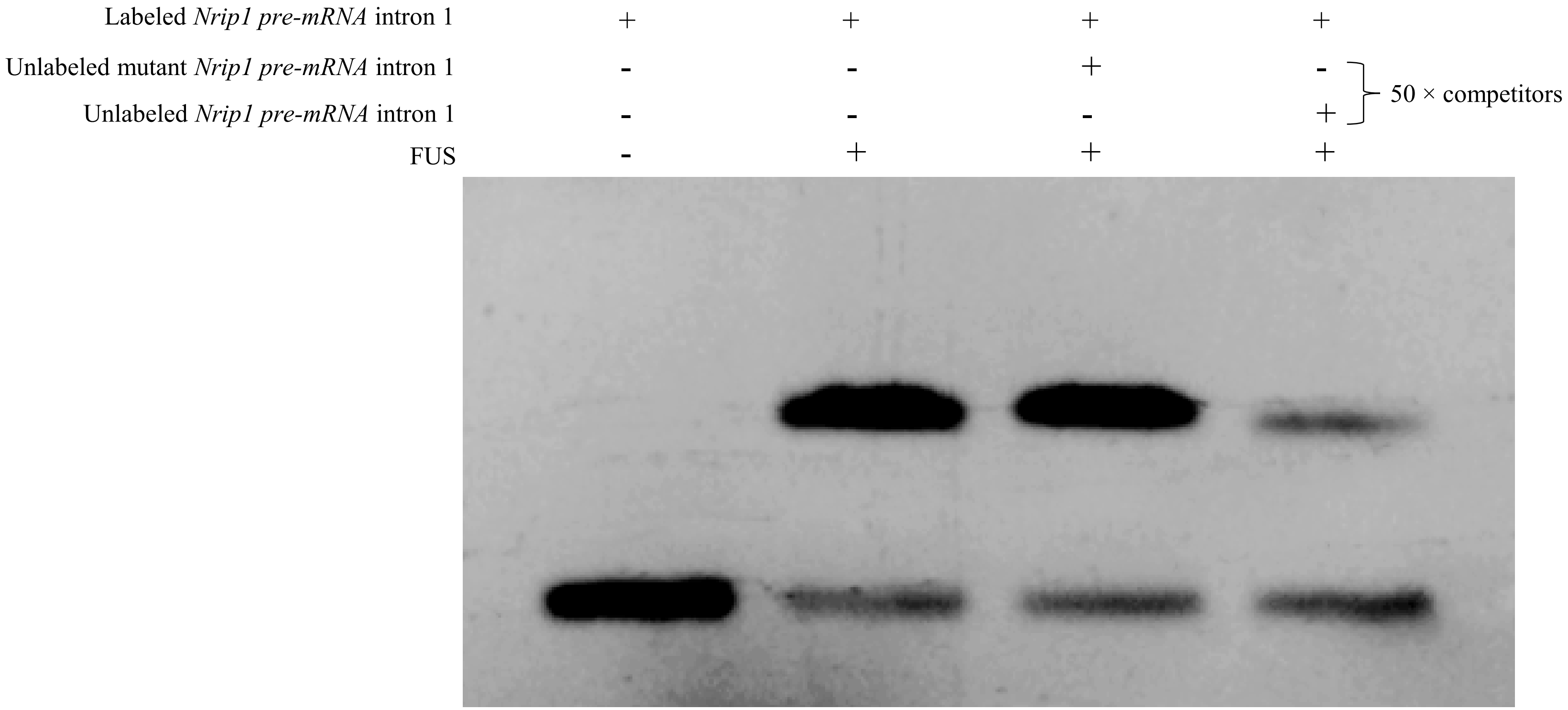

Supplementary Figure 6. Electrophoretic mobility shift analysis of interactions between FUS and intron 1 of *Nrip1 pre-mRNA*. Biotin-labeled *Nrip1 pre-mRNA* intron 1 (30 ng) was incubated with FUS protein (6 µg). 50-fold excess of unlabeled *Nrip1 pre-mRNA* intron 1 and unlabeled mutant *Nrip1 pre-mRNA* intron 1 were used as the competitors. Reactions were subjected to PAGE using native gels. After the biotin-labeled RNA was transferred to nylon membranes, the signal was detected using SA-HRP and ECL

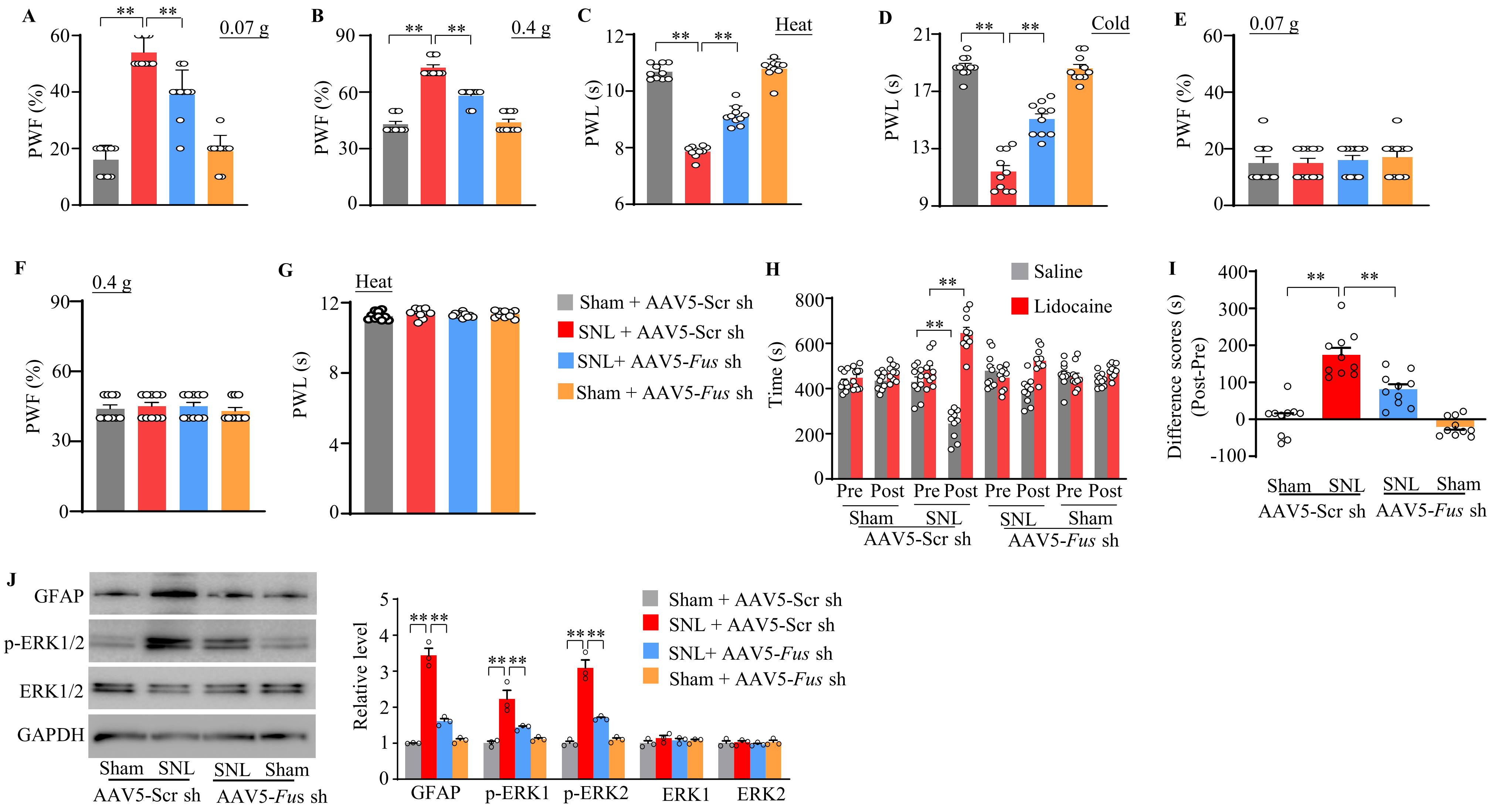

Supplementary Figure 7. Blocking nerve injury-induced increase of FUS in DRG attenuated the development of SNL-induced nociceptive hypersensitivity in male mice. (A-G) Effect of pre-microinjection of AAV5 expressing *Fus* shRNA (AAV5-*Fus* sh) or control scrambled shRNA (AAV5-Scr sh) into the ipsilateral L4 DRG 35 days before SNL or sham surgery on paw withdrawal frequency (PWF) to 0.07 g (A and E) and 0.4 g (B and F) von Frey filaments and on paw withdrawal latencies (PWL) to heat (C and G) and cold (H) stimuli on the ipsilateral (A-D) and contralateral (E-G) sides 14 days after surgery. n = 10 mice/group. $^{**}P < 0.01$ by 2-way ANOVA with repeated measures followed by post hoc Tukey test. (H and I) Effect of pre-microinjection of AAV5-*Fus* sh) or AAV5-Scr sh into the ipsilateral L4 DRG 35 days before SNL or sham surgery on spontaneous ongoing pain as assessed by the conditional place preference paradigm 14 days after surgery. Pre, preconditioning. Post, post-conditioning. n = 8 mice/group. $^{**}P < 0.01$ by 3-way (H) or 2-way (I) ANOVA with repeated measures followed by post hoc Tukey's test. (J) Levels of p-ERK1/2, total ERK1/2 and GFAP in the ipsilateral L4 dorsal horn on day 14 after SNL or sham surgery in the mice with pre-microinjection of AAV5-*Fus* sh or AAV5-Scr sh. n = 3 repeats (3 mice)/group. $^{**}P < 0.01$ by 2-way ANOVA followed by Tukey post hoc test.

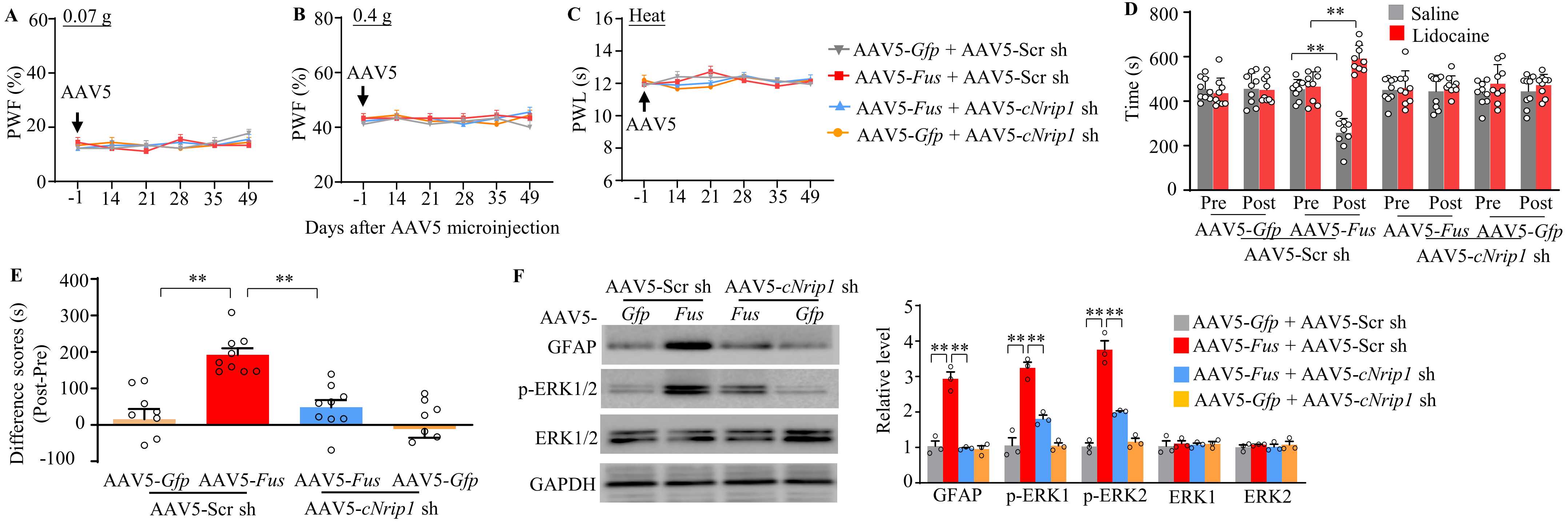

Supplementary Figure 8. Effect of DRG knockdown of *cNrip1* on basal behavioral responses on the contralateral side, spontaneous pain and dorsal horn neuronal and astrocyte hyperactivity in male mice with microinjection of AAV5 expressing full-length *Fus* mRNA (AAV5-*Fus*) or *Gfp* mRNA (AAV5-*Gfp*). (A-C) Effect of microinjection of AAV5-*cNrip1* sh) or AAV5-Scr sh into the ipsilateral L3/4 DRGs on paw withdrawal frequency (PWF) to 0.07 g (A) and 0.4 g (B) von Frey filaments and on paw withdrawal latencies (PWL) to heat (C) on the contralateral sides at time points as shown in the mice with microinjection of AAV5-*Fus* or AAV5-*Gfp* into unilateral L3/4 DRGs. n = 9 mice/group. 2-way ANOVA with repeated measures followed by post hoc Tukey test. (D and E) Effect of microinjection of AAV5-*cNrip1* sh or AAV5-Scr sh into the ipsilateral L3/4 DRGs on spontaneous ongoing pain as assessed by the conditional place preference paradigm 49 days after co-microinjection of AAV5-*Fus* or AAV5-*Gfp*. Pre, preconditioning. Post, post-conditioning. $n = 8$ mice/group. $^{**}P < 0.01$ by 2-way (D) or 1-way (E) ANOVA with repeated measures followed by post hoc Tukey's test. (F) Levels of p-ERK1/2, total ERK1/2 and GFAP in the ipsilateral L3/4 dorsal horn on day 49 after co-microinjection of different AAV5 as indicated. n = 3 repeats (3 mice)/treatment. $^{**}P < 0.01$ by one-way ANOVA followed by Tukey post hoc test.

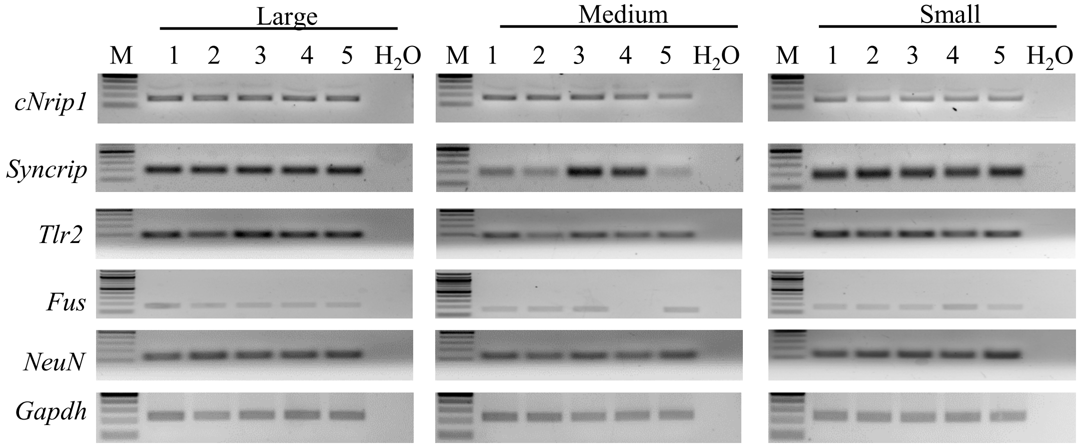

Supplementary Figure 9. Co-expression analysis of *cNrip1*, *Syncrip* mRNA, *Fus* mRNA and *Tlr2* mRNA in individual dorsal root ganglion (DRG) neurons by single-cell RT-PCR assay. *NeuN* mRNA is used as a neuronal marker. *Gapdh* mRNA was used as a loading control. Large DRG neuron: > 35 µm in diameter. Medium DRG neuron: 25-35 µm in diameter. Small DRG neuron: < 25 µm in diameter. n = 5 neurons/size. Number 1–5 indicates five different neurons. M: DNA ladder marker. H_2O, no cDNA, is a negative control.

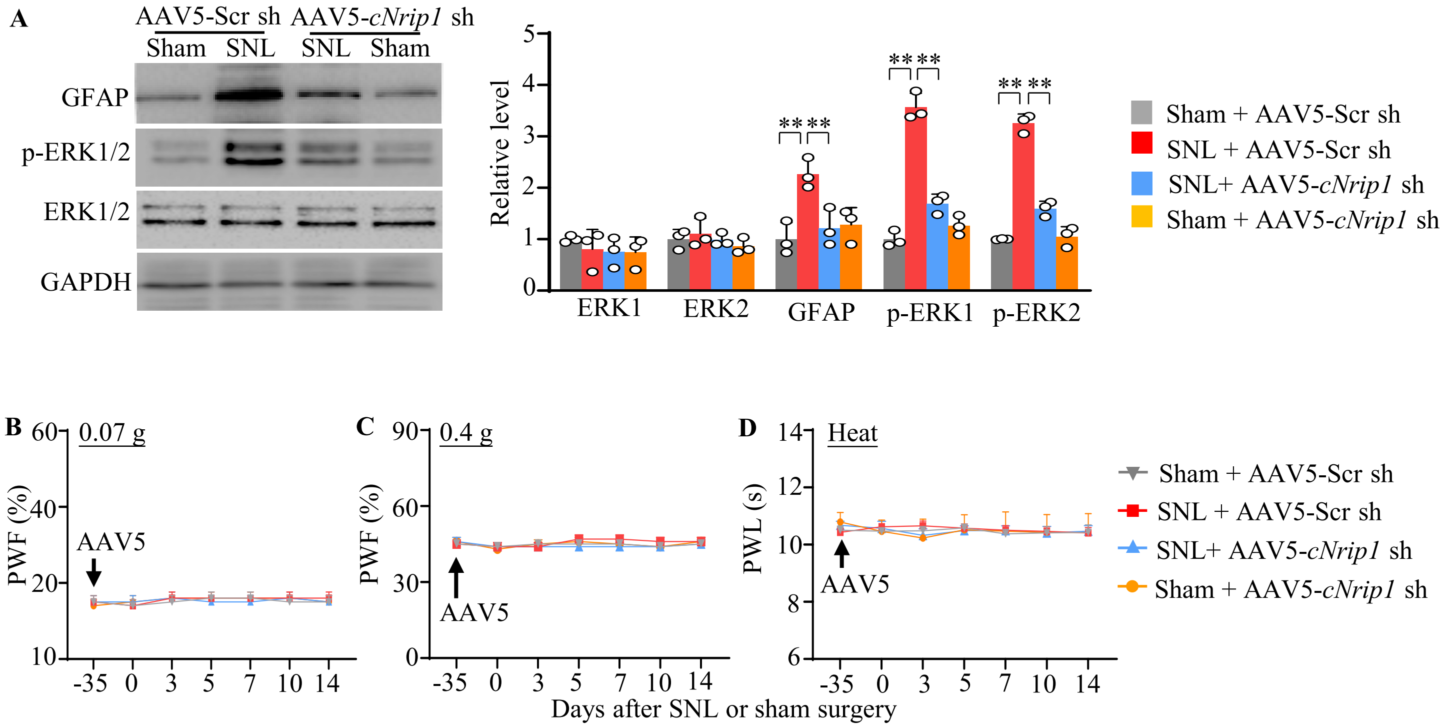

Supplementary Figure 10. Blocking DRG *cNrip1* upregulation mitigates the development of nerve injury-induced nociceptive hypersensitivity. (A) Levels of p-ERK1/2, total ERK1/2 and GFAP in the ipsilateral L4 dorsal horn on day 14 after SNL or sham surgery in male mice with pre-microinjection of AAV5-Scr sh or AAV5-*cNrip1* sh into the ipsilateral L4 DRG 35 days before SNL or sham surgery. n = 3 repeats (3 mice)/group. $^{**}P < 0.01$ by 2-way ANOVA followed by Tukey post hoc test. (B-D) Effect of pre-microinjection of AAV5-*cNrip1* sh or AAV5-Scr sh into the ipsilateral L4 DRG 35 days before SNL or sham surgery on paw withdrawal frequency (PWF) in response to 0.07 g (B) and 0.4 g (C) von Frey filament stimuli and paw withdrawal latency (PWL) in response to heat (D) on the contralateral side at indicated days after surgery. n = 8 mice/group. 3-way ANOVA with repeat measures followed by Tukey post hoc test.

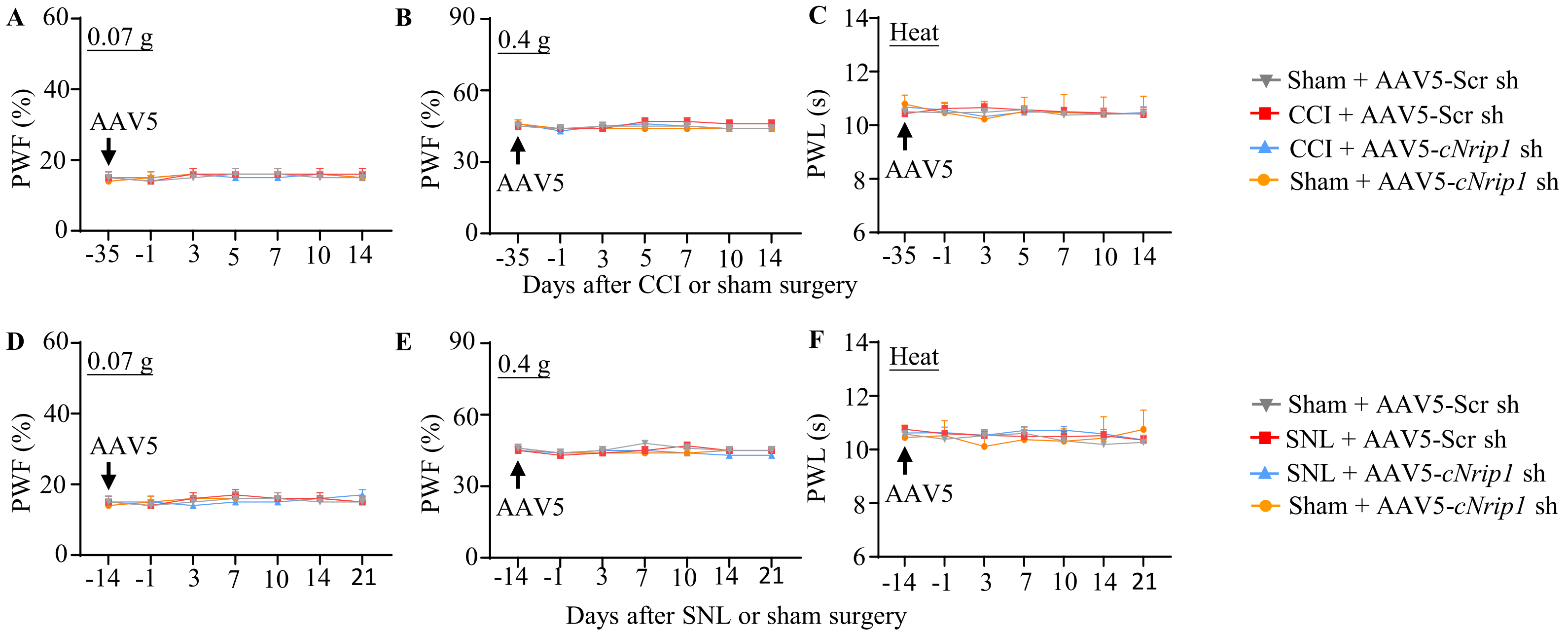

Supplementary Figure 11. Effect of blocking *cNrip1* upregulation in injured DRG on the contralateral paw withdrawal responses in male SNL/CCI mice. (A-C) Effect of pre-microinjection of AAV5-*cNrip1* sh or AAV5-Scr sh into the ipsilateral L3/4 DRGs 35 days before CCI or sham surgery on paw withdrawal frequency (PWF) in response to 0.07 g (A) and 0.4 g (B) von Frey filament stimuli and paw withdrawal latency (PWL) in response to heat (C) stimuli on the contralateral side at indicated days after surgery. n = 8 mice/group. 3-way ANOVA with repeat measures followed by post hoc Tukey's test. (D-F) Effect of pre-microinjection of AAV5-*cNrip1* sh or AAV5-Scr sh into the ipsilateral L3/4 DRGs 14 days before SNL or sham surgery on paw withdrawal frequency (PWF) in response to 0.07 g (D) and 0.4 g (E) von Frey filament stimuli and paw withdrawal latency (PWL) in response to heat (F) stimuli at indicated weeks after microinjection. n = 8 mice/group. 3-way ANOVA with repeat measures followed by post hoc Tukey's test.

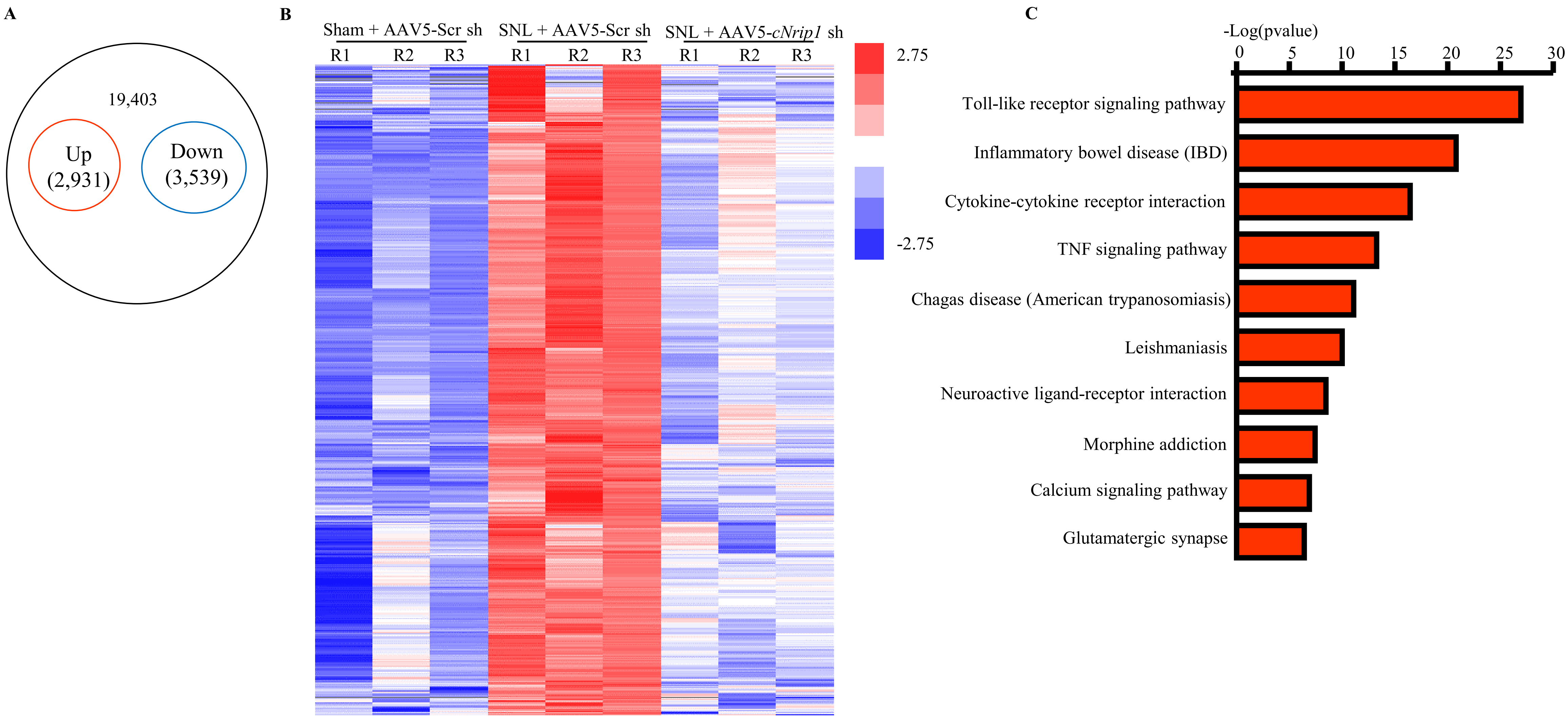

Supplementary Figure 12. Analysis of potential downstream targets of cNrip1 and their involvement in biological processes. (A) RNA sequencing analysis revealed that about 6,470 genes out of a total of 19,403 identified genes were significantly changed in the ipsilateral L4 DRG from the AAV5-Scr RNA-microinjected mice on day 7 post-SNL as compared to that post-sham surgery. Differentially expressed genes were filtered to $P < 0.05$ and $\log_2$fold-change. (B) Heatmaps showed that about 1,021 upregulated genes were reversed in the ipsilateral L4 DRG from the AAV5-*cNrip1* shRNA-microinjected mice on day 7 post-SNL. AAV5-*cNrip1* sh or AAV5-Scr sh was pre-microinjected into the ipsilateral L4 DRG 35 days before SNL or sham surgery. n = 3 repeats (R1, R2 and R3)/group. The scaled heat maps were created using ZA-score values obtained from RNA sequencing. High expression is shown by the red color spectrum, and low expression is shown in blue. (C) Analysis of the Gene Ontology database showed top 10 biological process functions of these differentially expressed genes.

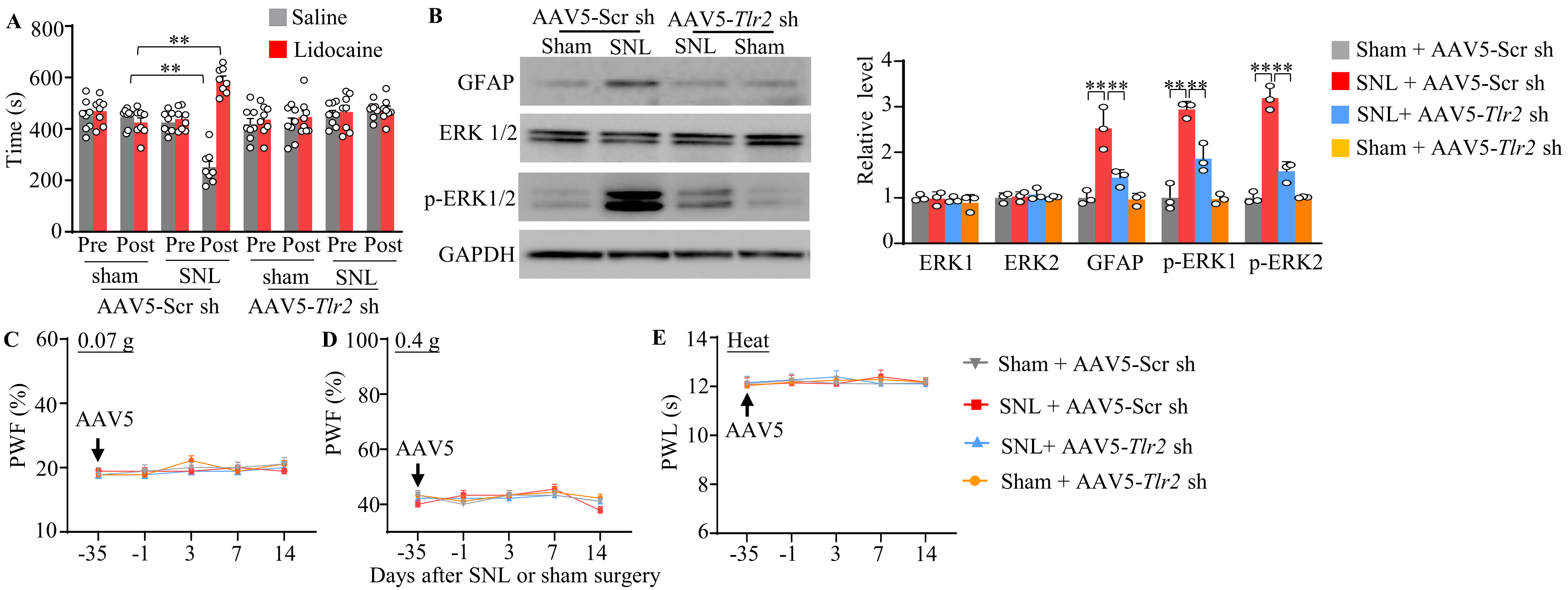

Supplementary Figure 13. Effect of blocking DRG TLR2 increase on SNL-induced spontaneous pain and dorsal horn neuronal and astrocyte hyperactivity as well as contralateral basal behavioral responses in male mice. (A) Effect of pre-microinjection of AAV5-*Tlr2* sh or AAV5-Scr sh into the ipsilateral L4 DRG 35 days before SNL or sham surgery on spontaneous ongoing pain as assessed by the conditional place preference paradigm 14 days after surgery. Pre, preconditioning; Post, post-conditioning. n = 8 mice/group. $^{**}P < 0.01$, by 3-way ANOVA with repeated measures followed by post hoc Tukey's test. (B) Levels of p-ERK1/2, total ERK1/2 and GFAP in the ipsilateral L4 dorsal horn on day 14 after SNL or sham surgery in male mice with pre-microinjection of AAV5-Scr sh or AAV5-*Tlr2* sh 35 days before surgery. n = 3 repeats (3 mice)/group. $^{**}P < 0.01$ by 2-way ANOVA followed by Tukey post hoc test. (C-E) Effect of pre-microinjection of AAV5-*Tlr2* sh or AAV5-Scr sh into the ipsilateral L4 DRG 35 days before SNL or sham surgery on paw withdrawal frequency (PWF) in response to 0.07 g (C) and 0.4 g (D) von Frey filament stimuli and paw withdrawal latency (PWL) in response to heat (E) stimuli on the contralateral side t indicated weeks after surgery. n = 9 mice/group. 3-way ANOVA with repeat measures followed by Tukey post hoc test.

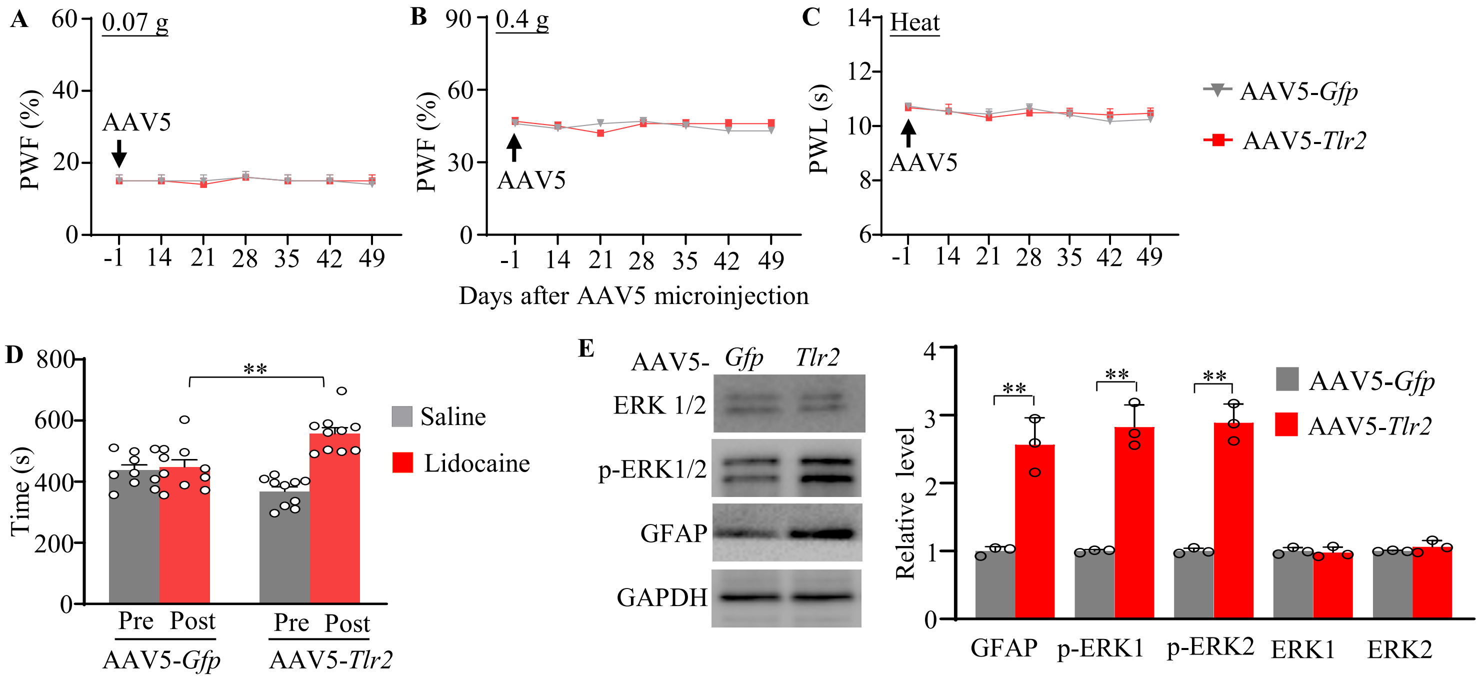

Supplementary Figure 14. Effect of DRG TLR2 overexpression on the contralateral basal behavioral responses and ipsilateral dorsal horn neuronal and astrocyte hyperactivity. (A-C) Effect of microinjection of AAV5-*Tlr2* or AAV5-*Gfp* into the ipsilateral L3/4 DRGs on paw withdrawal frequency (PWF) in response to 0.07 g (A) and 0.4 g (B) von Frey filament stimuli and paw withdrawal latency (PWL) in response to heat (C) stimuli on the contralateral side at indicated days after microinjection. n = 10 mice/group. 2-way ANOVA with repeated measures followed by post hoc Tukey's test. (D) Effect of microinjection of AAV5-*Tlr2* or AAV5-*Gfp* into the ipsilateral L3/4 DRGs on spontaneous ongoing pain as assessed by the conditional place preference paradigm 49 days after microinjection. Pre, preconditioning; Post, post-conditioning. n = 10 mice/group. $^{**}P < 0.01$ by 2-way ANOVA with repeated measures followed by post hoc Tukey's test. (E) Effect of microinjection of AAV5-*Tlr2* or AAV5-*Gfp* into the unilateral L3/4 DRGs on levels of p-ERK1/2, ERK1/2, and GFAP in the ipsilateral L3/4 dorsal horn 49 days after microinjection. n = 3 repeats (3 mice)/group. $^{**}P < 0.01$ by two-tailed, unpaired Student's t test.

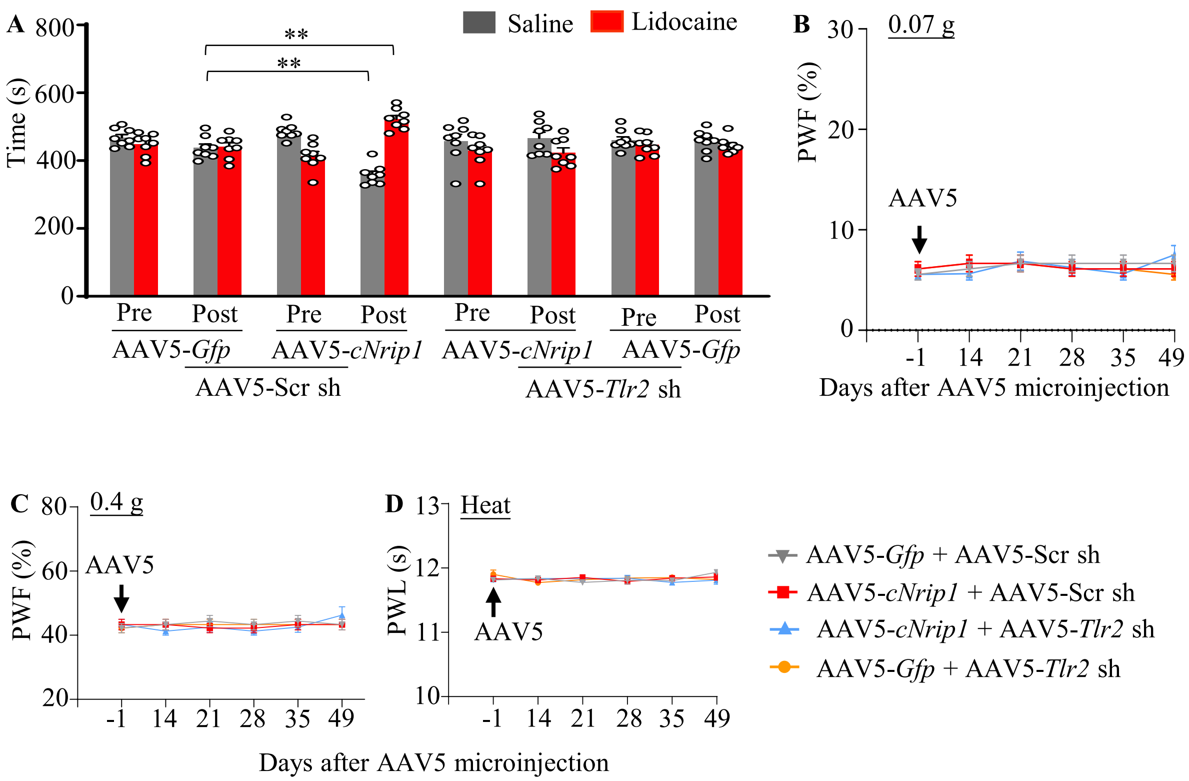

Supplementary Figure 15. Effect of DRG TLR2 knockdown on the *cNrip1* overexpression-induced spontaneous pain and the contralateral basal behavioral responses. (A) Effect of microinjection of AAV5-*Tlr2* sh or AAV5-Scr sh into the ipsilateral L4 DRGs on spontaneous ongoing pain as assessed by the conditional place preference paradigm 49 days after microinjection. Pre, preconditioning; Post, post-conditioning. $n = 8$ mice/group. $^{**}P < 0.01$ by 2-way ANOVA with repeated measures followed by post hoc Tukey's test. (B-D) Effect of microinjection of AAV5-*Tlr2* sh or AAV5-Scr sh into ipsilateral L3/4 DRGs on paw withdrawal frequency (PWF) to 0.07 g (B) and 0.4 g (C) von Frey filaments and on paw withdrawal latencies (PWL) to heat (D) stimuli on the contralateral side at time points as shown in the mice with co-microinjection of AAV5-c*Nrip1* or AAV5-*Gfp*. n = 9 mice/group. 2-way ANOVA with repeated measures followed by post hoc Tukey's test.

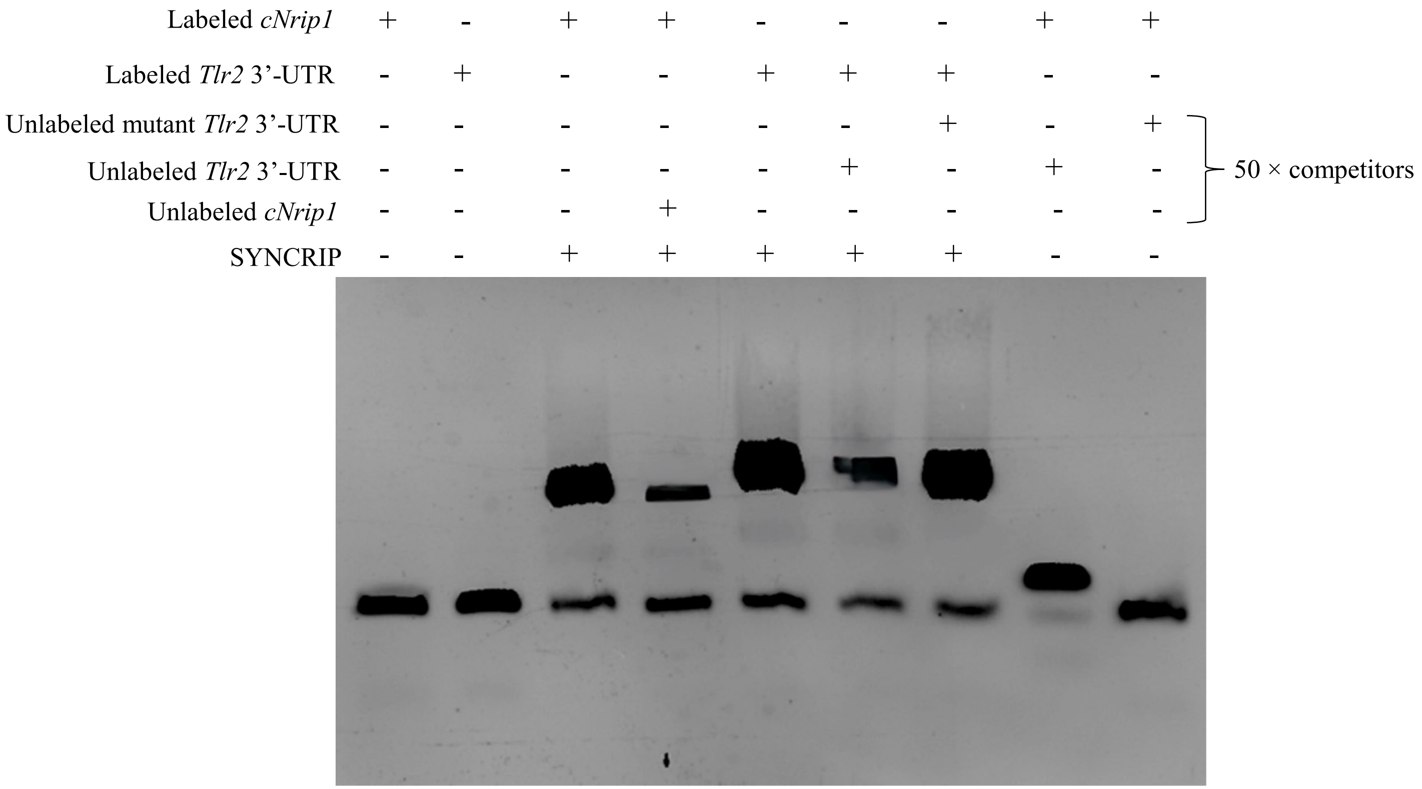

Supplementary Figure 16. Electrophoretic mobility shift analysis of interactions between SYNCRIP and *cNrip1* or *Tlr2* 3'-UTR as well as between *cNrip1* and *Tlr2* 3'-UTR. Biotin-labeled *cNrip1* (20 ng) or biotin-labeled *Tlr2* 3'-UTR (20 ng) was incubated with SYNCRIP protein (6 µg). 50-fold excess of unlabeled *cNrip1*, unlabeled *Tlr2 3'-UTR* and unlabeled mutant *Tlr2* 3'-UTR were used as the competitors. Reactions were subjected to PAGE using native gels. After the biotin-labeled RNA was transferred to nylon membranes, the signal was detected using SA-HRP and ECL

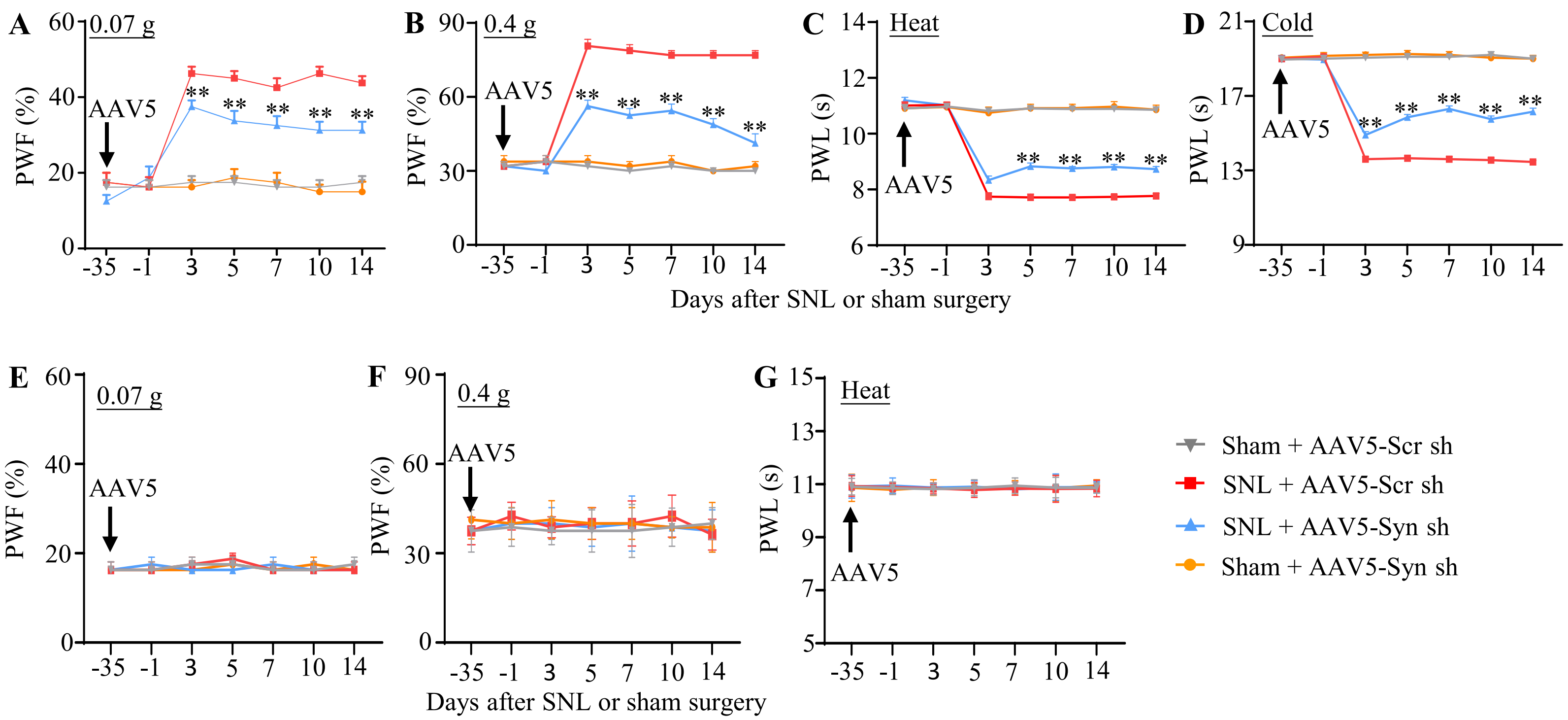

Supplementary Figure 17. Blocking DRG SYNCRIP increase alleviates the development of SNL-induced nociceptive hypersensitivity. (A-G) Effect of pre-microinjection of AAV5-Syn sh or AAV5-Scr sh into the ipsilateral L4 DRG 35 days before SNL or sham surgery on paw withdrawal frequency (PWF) in response to 0.07 g (A and E) and 0.4 g (B and F) von Frey filament stimuli and paw withdrawal latency (PWL) in response to heat (C and G) and cold (D) stimuli on the ipsilateral (A-D) and contralateral (E-G) sides at indicated days after surgery. n = 8 mice/group. $^{**}P < 0.01$ versus the AAV5-Scr sh-microinjected SNL mice at the corresponding days by 3-way ANOVA with repeated measures followed by post hoc Tukey's test.

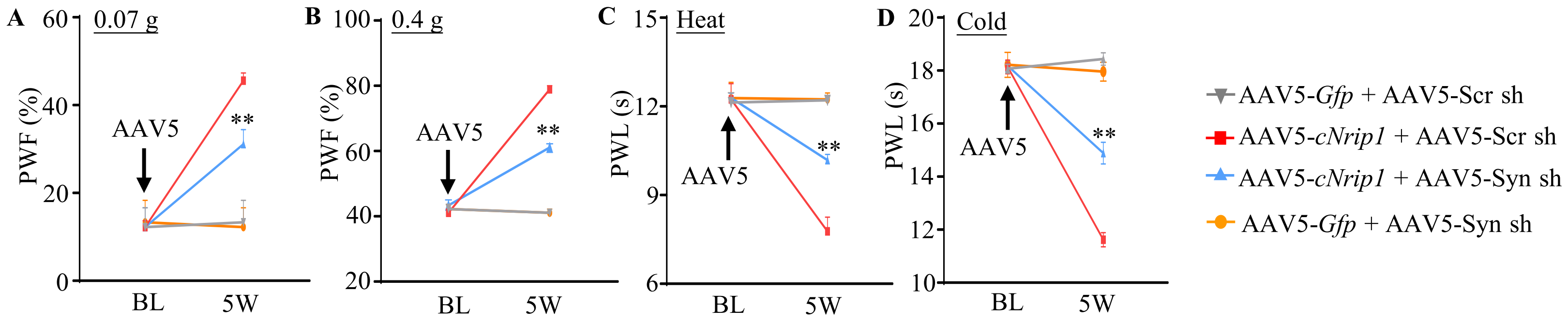

Supplementary Figure 18. DRG SYNCRIP knockdown mitigates nociceptive hypersensitivity induced by DRG *cNrip1* overexpression. (A-D) Effect of microinjection of AAV5-Syn sh or AAV5-Scr sh into the ipsilateral L3/4 DRGs on paw withdrawal frequency (PWF) in response to 0.07 g (A) and 0.4 g (B) von Frey filament stimuli and paw withdrawal latency (PWL) in response to heat (C) and cold (D) stimuli 5 weeks (W) after co-microinjection with AAV5-*cNrip1* or AAV5-*Gfp*. BL: baseline. n = 9 mice/group. **$P < 0.01$ versus the AAV5-*cNrip1* plus AAV5-Scr sh group by 2-way ANOVA with repeated measures followed by post hoc Tukey test.

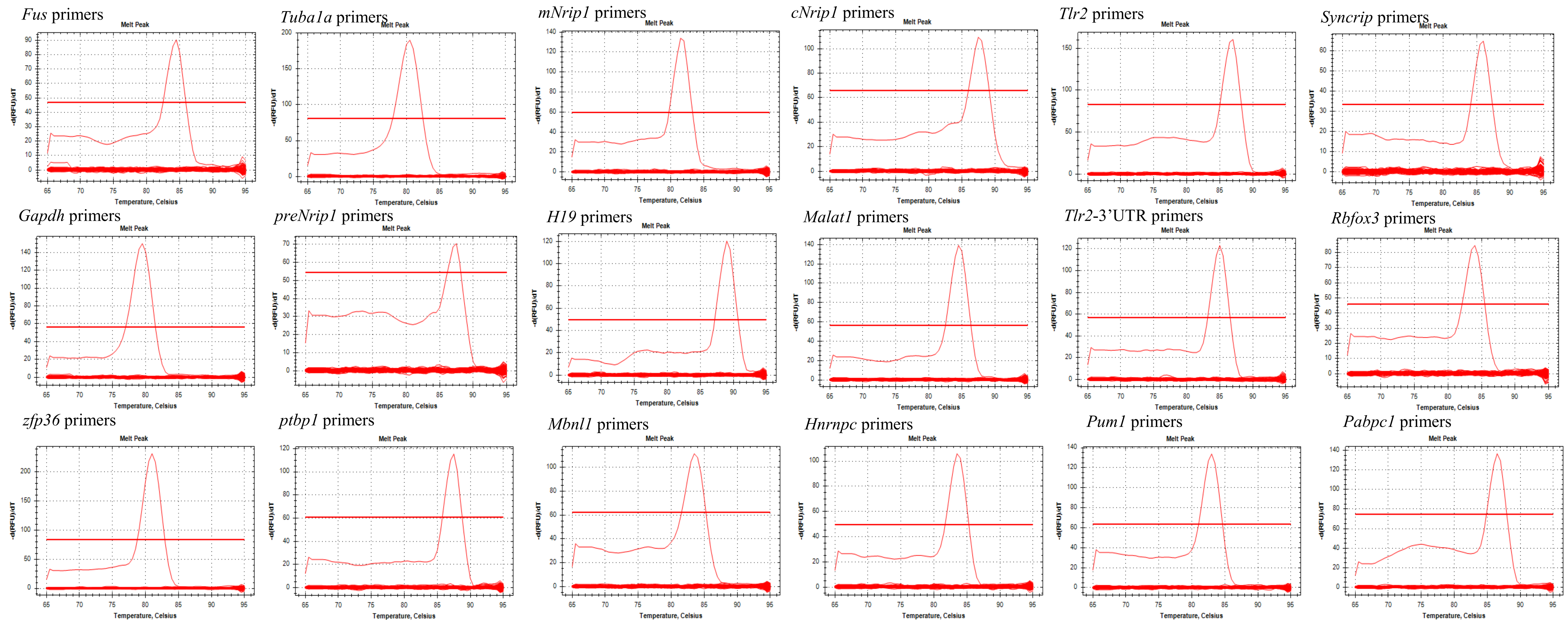

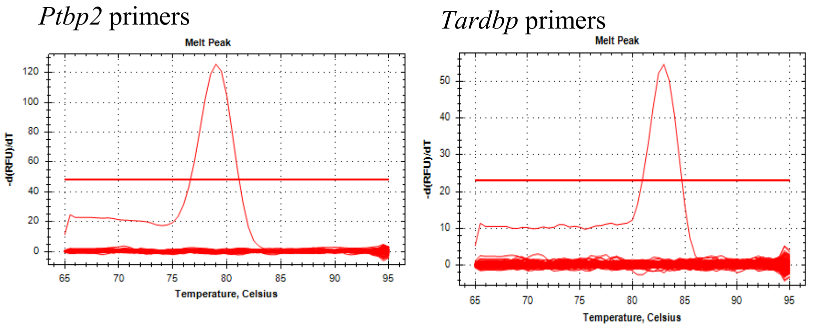

Supplementary Figure 19. Melting curve analysis was performed for all qPCR primers used, revealing a single sharp peak for each gene, which indicates specific amplification without detectable non-specific products or primer-dimer formation.

Supplementary Table 1: Locomotor function

Treatment groups	Placing	Grasping	Righting
Sham + AAV5-Scr sh (male)	5 (0)	5 (0)	5 (0)
Sham + AAV5-Fus sh (male)	5 (0)	5 (0)	5 (0)
SNL + AAV5-Scr sh (male)	5 (0)	5 (0)	5 (0)
SNL + AAV5-Fus sh (male)	5 (0)	5 (0)	5 (0)
AAV5-*Gfp* + AAV5-Scr sh (male)	5 (0)	5 (0)	5 (0)
AAV5-*Fus* + AAV5- Scr sh (male)	5 (0)	5 (0)	5 (0)
AAV5-*Fus* + AAV5-cNrip1 sh (male)	5 (0)	5 (0)	5 (0)
AAV5-*Gfp* + AAV5-cNrip1 sh (male)	5 (0)	5 (0)	5 (0)
Sham + AAV5-Scr sh (male)	5 (0)	5 (0)	5 (0)
Sham + AAV5-cNrip1 sh (male)	5 (0)	5 (0)	5 (0)
SNL + AAV5-Scr sh (male)	5 (0)	5 (0)	5 (0)
SNL + AAV5-cNrip1 sh (male)	5 (0)	5 (0)	5 (0)
Sham + AAV5-Scr sh (male)	5 (0)	5 (0)	5 (0)
Sham + AAV5-cNrip1 sh (male)	5 (0)	5 (0)	5 (0)
CCI + AAV5-Scr sh (male)	5 (0)	5 (0)	5 (0)
CCI + AAV5-cNrip1 sh (male)	5 (0)	5 (0)	5 (0)
Sham + AAV5-Scr sh (female)	5 (0)	5 (0)	5 (0)
Sham + AAV5-cNrip1 sh (female)	5 (0)	5 (0)	5 (0)
SNL + AAV5-Scr sh (female)	5 (0)	5 (0)	5 (0)
SNL + AAV5-cNrip1 sh (female)	5 (0)	5 (0)	5 (0)
AAV5-*Gfp* (male)	5 (0)	5 (0)	5 (0)
AAV5-*cNrip1* (male)	5 (0)	5 (0)	5 (0)
AAV5-*Gfp* (female)	5 (0)	5 (0)	5 (0)
AAV5-*cNrip1* (female)	5 (0)	5 (0)	5 (0)
Sham + AAV5-Scr sh (male)	5 (0)	5 (0)	5 (0)
Sham + AAV5-TLR2 sh (male)	5 (0)	5 (0)	5 (0)
SNL + AAV5-Scr sh (male)	5 (0)	5 (0)	5 (0)
SNL + AAV5- TLR2 sh (male)	5 (0)	5 (0)	5 (0)
AAV5-*Gfp* (male)	5 (0)	5 (0)	5 (0)
AAV-*Tlr2* (male)	5 (0)	5 (0)	5 (0)
AAV5-*Gfp* + AAV5-Scr sh (male)	5 (0)	5 (0)	5 (0)
AAV5-*cNrip1* + AAV5- Scr sh (male)	5 (0)	5 (0)	5 (0)
AAV5-*cNrip1* + AAV5-TLR2 sh (male)	5 (0)	5 (0)	5 (0)
AAV5-*Gfp* + AAV5-TLR2 sh (male)	5 (0)	5 (0)	5 (0)
Sham + AAV5-Scr sh (male)	5 (0)	5 (0)	5 (0)
SNL + AAV5-Scr sh (male)	5 (0)	5 (0)	5 (0)
SNL + AAV5-Syn sh (male)	5 (0)	5 (0)	5 (0)
Sham + AAV5-Syn sh (male)	5 (0)	5 (0)	5 (0)
AAV5-*Gfp* + AAV5-Scr sh (male)	5 (0)	5 (0)	5 (0)
AAV5-*cNrip1* + AAV5-Scr sh (male)	5 (0)	5 (0)	5 (0)
AAV5-*cNrip1*+ AAV5-Syn sh (male)	5 (0)	5 (0)	5 (0)
AAV5-*Gfp* + AAV5-Syn sh (male)	5 (0)	5 (0)	5 (0)

n = 8 mice per group; 5 trials; Mean (SEM).

Supplementary Table 2: Potential *cNrip1* binding proteins identified by using ChIRP-MS assay

Description	*cNrip1*	*mNrip1*	Ratio
hnrnpc	3.5	1.5	2.33
krt31	2	1	2
aldoa	2	1	2
asap2	2	1	2
cep162	2	1	2
Syncrip	1.9	1.18	1.61
Krt10	4	2.67	1.5
Dsp	1.5	1	1.5
Eef1g	1.5	1	1. 5
Rps12	1.5	1	1.5
Ciart	1.5	1	1.5
Sprr1b	1.5	1	1.5
Dsg1a	1.5	1	1.5
Dld	1.5	1	1.5
Eif4h	1.5	1	1.5
Eif5a	1.5	1	1.5
Gpi	1.5	1	1.5
Ilf3	1.5	1	1.5
Lpl	1.5	1	1.5
Map1b	1.5	1	1.5
Mbnl	1.5	1	1.5
Snu	1.5	1	1.5
Pafah1b	1.5	1	1.5
Psmb3	1.5	1	1.5
Rbm	1.5	1	1.5
Strap	1.5	1	1.5
Esd	1.5	1	1.5
Tppp	1.5	1	1.5
C2orf81	1.5	1	1.5

cNrip1: biotinylated full-length sense *cNrip1* RNA, *mNrip1:* biotinylated full-length sense *mNrip1* RNA

Supplemental Table 3. All primers, probes used

Names	Sequences	Names	Sequences
**RT-PCR**		**Vector construction**	
m/h-*cNrip1* F	5′- GCCCGGAGAATCTGAAGAC-3′	AAV5-*cNrip1* F	5′-CTAAATGAAACCTTGCTTCTTACCGTCTGCCTCAGCCTGAGCCT-3′
m/h-*cNrip1* R	5′- GATGTGTTCCTCCCGAATGT-3′	AAV5-*cNrip1* R	5′-AAGGCGTGGACTGTGAGCGGTTGCAGAGCTGTTCTC -3′
M-*mNrip1* F	5′- CTGGGCTTTTGAATGGATGT -3′	AAV5-*Tlr2* F	5′- GGCCTCGAGATGCTACGAGCTCTTTGGCTC-3′
M-*mNrip1* R	5′- GCTCCCTCCAGATGTCTTTG -3′	AAV5-*Tlr2* R	5′- CGCGCGGCCGCCTAGGACTTTATTGCAGTTCTC-3′
m-*Tlr2* F	5′- TTCACCACTGCCCGTAGATG -3′	AAV5-*Syn* F	5′- GGCTCCGGAATGGCTACAGAACATGTTAATG-3′
m-*Tlr2* R	5′- CGCTCACTACGTCTGACTCC -3′	AAV5-*Syn* R	5′- CATGCGGCCGCTCATTGTAACAGGTCAG-3′
m-*Syn* F	5′- GATGAGGCAAAGATTAAG -3′	AAV5-*Fus* F	5′- TAGAAGCTTGCCACCATGGCTTCAAACGACTA -3′
m-*Syn* R	5′- CCATATAGGTCCAGCTTTCTC -3′	AAV5-*Fus* R	5′- GCACTCGAGCTAATATGGCCTCTCCCT -3′
m-*Fus* F	5′- GGCTACTCCCAACAGAGCAG -3′	AAV5-Fus sh F	5′- GATCCGTGGAGGTTATGGTCAACAGAAGCTTGTGTTGACCATAACCTCCACTTTTTTT-3′
m-*Fus* R	5′- ATATCCCTGGGGAGCTGACT -3′	AAV5-Fus sh R	5′- CTAGAAAAAAAGTGGAGGTTATGGTCAACACAAGCTTCTGTTGACCATAACCTCCACG-3′
m-*Gapdh* F	5′- TTGTGGAAGGGCTCATGACC- 3′	AAV5-cNrip1 sh F	5′- GATCCGTCCACGCCTTCGTCTGCCTCGAAGCTTG GAGGCAGACGAAGGCGTGGACTTTTTTT-3′
m-*Gapdh* R	5′-GTTGAAGTCGCAGGAGACAA -3′	AAV5-cNrip1 sh R	5′- CTAGAAAAAAAGTCCACGCCTTCGTCTGCCTC CAAGCTTCCGAGGCAGACGAAGGCGTGGACG -3′
m-*Tuba1a* F	5′- GTGCATCTCCATCCATGTTG-3′	AAV5-TLR2 sh F	5′- GATCCCTGTCTTTCAACAAGAAGTCCGAAGCTTGGGACTTCTTGTTGAAAGACAGTTTTTTT -3′
m-*Tuba1a* R	5′- GTGGGTTCCAGGTCTACGAA-3′	AAV5-TLR2 sh R	5′- CTAGAAAAAAGGTGATCTTGTTGAAAGACAGCAAGCTTCCTGTCTTTCAACAAGATCACCG -3′
m-*Tlr2*-3'UTR F	5′- GTTCTCCACCCAGTTCCTGACTTC -3′	AAV5-Syn sh F	5′- GATCCATACTACTTCAGCAGTTATCCGAAGCTTG GGATAACTGCTGAAGTAGTATTTTTTTT -3′
m-*Tlr2*-3'UTR R	5′- TCCTGATGTCACATATTTATATAA -3′	AAV5-Syn sh R	5′- CTAGAAAAAAAATACTACTTCAGCAGTTATCCCAAGCTTCGGATAACTGCTGAAGTAGTATG -3′
m-*H19* F	5′- CATTCTAGGCTGGGGTCAAA-3′	*psiCheck*-Tlr2-3'UTR F	5′- AGCGGTACCGTTCTCCACCCAGTTCCTGAC -3′
m-*H19* R	5′- GCCCTTCTTTTCCATTCTCC-3′	*psiCheck*-Tlr2-3'UTR R	5′- GGCGCTAGCTCCTGATGTCACATATTTATATAAC -3′
m-*Malat1* F	5′- TCATACCTAACCAGGCATAACA-3′		
m-*Malat1* R	5′- AAGTGCTCACAAGGCAAATC-3′	**Single cell RT-PCR**	
h-*Gapdh* F	5′- TCACCATCTTCCAGGAGCG -3′	m-*cNrip1* F	5′- GCCCGGAGAATCTGAAGAC-3′
h-*Gapdh* R	5′- CTGCTTCACCACCTTCTTGA -3′	m-*cNrip1* R	5′- GATGTGTTCCTCCCGAATGT-3′
m-*Rbfox3* (NeuN) F	5′- AGCCTGGGAACCCATATGCC-3′	m-*Tlr2* F	5′- TTCACCACTGCCCGTAGATG -3′
m-*Rbfox3* (NeuN) R	5′- CATCCTGATACACGACCGCT-3′	m-*Tlr2* R	5′- CGCTCACTACGTCTGACTCC -3′
m- *preNrip1* F	5′- GGTGCCCACCATGTGTACT-3′	m-*Syn* F	5′- GATGAGGCAAAGATTAAG -3′
m- *preNrip1* R	5′- CAACCATTGGACTGAGCACG-3′	m-*Syn* R	5′- CCATATAGGTCCAGCTTTCTC -3′
m- *Tardbp* F	5′- AGCTTTTGCCTTCGTCACCT -3′	m-*Fus* F	5′- GGCTACTCCCAACAGAGCAG -3′
m-*Tardbp* R	5′- TGAGATGAACCTGATTCCCAAAG -3′	m-*Fus* R	5′- ATATCCCTGGGGAGCTGACT -3′
m-*zfp36* F	5′- TACGAGAGCCTCCAGTCGAT -3′	m-*Rbfox3* (NeuN) F	5′- ACATTTCGAGCTGCACCA-3′
m-*zfp36* R	5′- GGGAGCCAAAGGTGCAAAAC -3′	m-*Rbfox3* (NeuN) R	5′- CCGATGGTGTGATGGTAAGG-3′
m-*ptbp1* F	5′- GTCAGCAATCTGAACCCTGAGA -3′	m-*Gapdh* F	5′- TTGTGGAAGGGCTCATGACC- 3′
m-*ptbp1* R	5′- GTAATGCGCACTGACTTCCC -3′	m-*Gapdh* R	5′- GTTGAAGTCGCAGGAGACAA -3′
m-*Mbnl1* F	5′- CATCAGCAGCCTTTAACCCTT -3′		
m-*Mbnl1* R	5′- CCACGCTGGTACTCTCGAC -3′		
m-*Hnrnpc* F	5′- AAGCAGACTTGTCCTTCTCATC -3′	***cNrip1* Probe for Northern Blot**	
m-*Hnrnpc* R	5′- CCTCAGACTCCATCTTCACATTAG -3′	F	5′- TAATACGACTCACTATAGGAGGCCCGGAGAATCTGAA-3'
m-*Pum1* F	5′- GACATTCACTTACGCCACCG -3′	R	5′- TCAGTCAACTGCGCACCTCC -3′
m-*Pum1* R	5′- GGAGGGCATGATGTCAGATCTATT -3′		
m-*Pabpc1* F	5′- CTGCGCCCCCGCAAGA -3′	***cNrip1* full length for Mass**	
m-*Pabpc1* R	5′- CTTTCGCTTGGTGGGCTTGT -3′	F	5′- TAATACGACTCACTATAGGGGATGTGTTCCTCCCGAATGT-3'
m-*Ptbp2* F	5′- CCGGTAAACGCTCAACAAGC -3′	R	5′- GCCCGGAGAATCTGAAGAC-3′
m-*Ptbp2* R	5′- CAGCAATGGCTGGGTCTAAC -3′		

RT: Reverse-transcription; F, Forward; R, Reverse. m-, mice. h-, human. *cNrip1*, *circNrip1*. *mNrip1*, *Nrip1* mRNA. sh, short hairpin RNA. Syn, Syncrip. Mass, Mass Spectrometry

Supplemental Table 4. *p* and *F* values

Fig	*p* value	*F* value
**Main Figurs**		
2A	P<0.0001, P<0.0001, P<0.0001	F_{model}= 144.7, F_{time}= 13.14, $F_{model \times time}$= 11.63
2A	P=0.3913, P=0.6095, P=0.9984	F_{model}= 4.329e-005, F_{time}= 1.090, $F_{model \times time}$= 2.799
2B	P<0.0001	F= 53.35
2C	P<0.0001	F= 79.28
2D	P<0.0001	F= 33.06
2F	P<0.0001, P<0.0001, P<0.0001	$F_{treatment}$ = 84.34, F_{time}= 0.6137, $F_{treatment \times time}$= 0.1941
2G	P<0.0001, P<0.0001, P<0.0001	$F_{treatment}$ = 898.0, F_{time}= 0. 6.892, $F_{treatment \times time}$= 0.1941
3A	P=0.0079	t=11.20
3B	P=0.0002, P=0.0007, P=0.9477	F_{Fus}= 23.62, F_{cNrip1}= 17.82, F_{mNrip1}= 0.1169
3B	P=0.0001, P=0.0005	F= 34.44
3C	P=0.0001, P<0.0001, P=0.2800	F_{Fus}= 18.05, F_{cNrip1}= 48.14, F_{mNrip1}= 1.529
3C	P=0.0001, P=0.0015	F= 32.59
3D	P=0.0008, P=0.0004, P=0.4869	F_{Fus}= 16.73, F_{cNrip1}= 20.86, F_{mNrip1}= 0.8896
3D	P=0.0001, P=0.0015	F= 8.416
3E	P<0.0001, P<0.0001, P<0.0001	F_{time}= 69.23, $F_{treatment}$= 14.87, $F_{treatment \times time}$= 10.14
3F	P<0.0001, P<0.0001, P<0.0001	F_{time}= 48.24, $F_{treatment}$= 10.13, $F_{treatment \times time}$= 11.32
3G	P<0.0001, P<0.0001, P<0.0001	F_{time}= 67.48, $F_{treatment}$= 12.34, $F_{treatment \times time}$= 13.93
3H	P<0.0001, P<0.0001, P<0.0001	F_{time}= 122.6, $F_{treatment}$= 13.88, $F_{treatment \times time}$= 15.68
4A	P<0.0001, P=0.0735	F_{cNrip1}= 95.42, F_{mNrip1}= 2.988
4B	P<0.0001, P<0.0001, P<0.0001	F_{time}= 59.81, $F_{treatment}$= 26.65, $F_{treatment \times time}$= 33.56
4C	P<0.0001, P<0.0001, P<0.0001	F_{time}= 271.9, $F_{treatment}$= 82.72, $F_{treatment \times time}$= 86.39
4D	P<0.0001, P<0.0001, P<0.0001	F_{time}= 86.06, $F_{treatment}$= 9.377, $F_{treatment \times time}$= 9.660
4E	P<0.0001, P<0.0001, P<0.0001	F_{time}= 118, $F_{treatment}$= 10.62, $F_{treatment \times time}$= 10.47
4F	P<0.0001, P=0.6361	F_{cNrip1}= 26.85, F_{mNrip1}= 0.5944
4G	P<0.0001, P<0.0001, P<0.0001	F_{time}= 59.81, $F_{treatment}$= 26.65, $F_{treatment \times time}$= 33.56
4H	P<0.0001, P<0.0001, P<0.0001	F_{time}= 271.9, $F_{treatment}$= 82.72, $F_{treatment \times time}$= 86.39
4I	P<0.0001, P<0.0001, P<0.0001	F_{time}= 86.06, $F_{treatment}$= 9.377, $F_{treatment \times time}$= 9.660
4J	P<0.0001, P<0.0001, P<0.0001	F_{time}= 235.5, $F_{treatment}$= 38.64, $F_{treatment \times time}$= 45.4
4K	P<0.0001, P=0.1946	F_{cNrip1}= 21.87, F_{mNrip1}= 1.834
4L	P<0.0001, P<0.0001, P=0.0002	F_{time}= 54.32, $F_{treatment}$= 17.95, $F_{treatment \times time}$= 14.37
4M	P<0.0001, P<0.0001, P<0.0001	F_{time}= 437.5, $F_{treatment}$= 20.91, $F_{treatment \times time}$= 15.91
4N	P<0.0001, P<0.0001, P<0.0001	F_{time}= 363.2, $F_{treatment}$= 16.9, $F_{treatment \times time}$= 16.64
4O	P<0.0001, P<0.0001, P<0.0001	F_{time}= 471.2, $F_{treatment}$= 15.74, $F_{treatment \times time}$= 17.93
5A	P= 0.0039, P= 0.0024	F_{cNrip1}= 12.8
5B	P<0.0001, P<0.0001, P<0.0001	F_{time}= 181.8, $F_{treatment}$= 32.84, $F_{treatment \times time}$= 32.84
5C	P<0.0001, P<0.0001, P<0.0001	F_{time}= 205.4, $F_{treatment}$= 26.75, $F_{treatment \times time}$= 23.78
5D	P<0.0001, P<0.0001, P<0.0001	F_{time}= 498, $F_{treatment}$= 31.33, $F_{treatment \times time}$= 18.32
5E	P<0.0001, P<0.0001, P<0.0001	F_{time}= 314.1, $F_{treatment}$= 18.37, $F_{treatment \times time}$= 29.85
5F	P>0.9999, P=0.7244, P>0.9999	F_{time}= 0.000, $F_{treatment}$= 0.1250, $F_{treatment \times time}$= 0.000
5G	P= 0.8785, P= 0.6468, P= 0.8785	F_{time}= 0.2113, $F_{treatment}$= 0.02347, $F_{treatment \times time}$= 0.02347
5H	P= 0.5320, P= 0.0769, P= 0.2032	F_{time}= 0.3933, $F_{treatment}$= 3.196, $F_{treatment \times time}$= 0.03933
5I	P<0.0001, P<0.0001	F= 44.68, F= 39.63
5J	P<0.0001, P<0.0001	F= 29.36
5K	p_{GFAP} <0.0001, $p_{p\text{-}ERK1}$ <0.0001, $p_{p\text{-}ERK2}$ <0.0001, p_{ERK1}= 0.9431, p_{ERK2}= 0.8364	F_{GFAP}= 54.03, $F_{p\text{-}ERK1}$=45.63, F_{pERK2}= 73.34, F_{ERK1}= 0.1243, F_{ERK2}= 0.2829
6A	P_{cNrip1}=0.0001, P_{mNrip1}=0.888	t_{cNrip1}= 14.60, t_{mNrip1}=0.1501
6B	P<0.0001, P<0.0001, P<0.0001	F_{time}= 28.11, $F_{treatment}$= 470.4, $F_{treatment \times time}$= 35.29
6C	P<0.0001, P<0.0001, P<0.0001	F_{time}= 12.46, $F_{treatment}$= 183.5, $F_{treatment \times time}$= 10.6
6D	P<0.0001, P<0.0001, P<0.0001	F_{time}= 49.77, $F_{treatment}$= 1028, $F_{treatment \times time}$= 45.76
6E	P<0.0001, P<0.0001, P<0.0001	F_{time}= 38.77, $F_{treatment}$= 661.4, $F_{treatment \times time}$= 31.07
6F	P=0.2533, P=0.1001, P=0.1122	F_{time}= 1.313, $F_{treatment}$=0.740, $F_{treatment \times time}$= 1.698
6G	P=0.4146, P=0.0809, P=0.3062	F_{time}= 1.026, $F_{treatment}$= 1.792, $F_{treatment \times time}$= 1.455
6H	P=0.3860, P=0.1320, P=0.3456	F_{time}= 1.068, $F_{treatment}$= 1.622, $F_{treatment \times time}$= 0.8942
6I	P<0.0001	t= 50.09
6J	P<0.0001	t= 6.907
6K	p_{GFAP}= 0.0071, $p_{p\text{-}ERK1}$=0.0031, $p_{p\text{-}ERK2}$= 0.0060, p_{ERK1}= 0.636, p_{ERK2}= 0.8499	F_{GFAP}= 5.609, $F_{p\text{-}ERK1}$=6.366, F_{pERK2}= 5.315, F_{ERK1}= 2.546, F_{ERK2}= 0.2018
7A	P_{cNrip1}=0.0015, Pm_{Nrip1}=0.6369	F_{cNrip1}= 7.697, Fm_{Nrip1}= 0.5099
7B	P<0.0001, P<0.0001, P<0.0001	F_{time}= 29.86, $F_{treatment}$= 321.4, $F_{treatment \times time}$= 27.64
7C	P<0.0001, P<0.0001, P<0.0001	F_{time}= 15.19, $F_{treatment}$= 163.5, $F_{treatment \times time}$= 13.85
7D	P<0.0001, P<0.0001, P<0.0001	F_{time}= 35.27, $F_{treatment}$= 393.9, $F_{treatment \times time}$= 33.24
7E	P<0.0001, P<0.0001, P<0.0001	F_{time}= 38.77, $F_{treatment}$= 661.4, $F_{treatment \times time}$= 31.07
7F	P=0.8289, P=0.9287, P=0.8928	F_{time}= 0.9287, $F_{treatment}$=0.04698, $F_{treatment \times time}$= 0.8928
7G	P=0.9133, P=0.2733, P=0. 2181	F_{time}= 0.3415, $F_{treatment}$= 1.280, $F_{treatment \times time}$= 1.537
7H	P=0.3123, P=0.2638, P=0.0801	F_{time}= 1.201, $F_{treatment}$= 1301, $F_{treatment \times time}$= 3.106
7I	P<0.0001	F= 44.66
7J	P=0.0008	t= 4.282
7K	p_{GFAP}= 0.0016, $p_{p\text{-}ERK1}$=0.0005, $p_{p\text{-}ERK2}$ <0.0001, p_{ERK1}= 0.2922, p_{ERK2}= 0.6189	F_{GFAP}= 7.655, $F_{p\text{-}ERK1}$=10.32, F_{pERK2}= 17.96, F_{ERK1}= 1.212, F_{ERK2}= 0.5383
8A	P<0.0001, P<0.0001, P<0.0001	F_{model}= 28.25, F_{time}= 27.4, $F_{model \times time}$= 164
8B	P <0.0001	F= 103.6
8C	P=0.0007	F= 17.54
8D	P=0.0017, P=0.0097	F= 16.34
8E	P<0.0001, P<0.0001, P<0.0001	F_{time}= 29.45, $F_{treatment}$= 206.4, $F_{treatment \times time}$= 40.76
8F	P<0.0001, P<0.0001, P<0.0001	F_{time}= 240.4, $F_{treatment}$=98.36, $F_{treatment \times time}$= 109.8
8G	P<0.0001, P<0.0001, P<0.0001	F_{time}= 272.2, $F_{treatment}$= 46.76, $F_{treatment \times time}$= 30.41
8H	P<0.0001, P<0.0001, P<0.0001	F_{time}= 322.7, $F_{treatment}$= 87.52, $F_{treatment \times time}$= 99.30
8I	P<0.0001, P=0.0035	F= 22.15
8J	P=0.0001	F= 14.55
8K	P=0.0086	t= 4.808
8L	P<0.0001, P<0.0001, P<0.0001	F_{time}= 242.6, $F_{treatment}$= 20.55, $F_{treatment \times time}$= 18.76
8M	P<0.0001, P<0.0001, P<0.0001	F_{time}= 157.1, $F_{treatment}$= 16.05, $F_{treatment \times time}$= 14.74
8N	P<0.0001, P<0.0001, P<0.0001	F_{time}= 153.2, $F_{treatment}$= 12.64, $F_{treatment \times time}$= 11.80
8O	P<0.0001, P<0.0001, P<0.0001	F_{time}= 202.8, $F_{treatment}$= 16.85, $F_{treatment \times time}$= 15.48
8P	P=0.0061	t= 3.105

Supplemental Table 4. *p* and *F* values

Fig	*p* value	*F* value
9A	P<0.0001, P<0.0001	F= 61.92
9B	P<0.0001, P<0.0001	F= 518.3
9C	P<0.0001, P<0.0001, P=0. 9188	F_{cNrip1}= 36.09, F_{Tlr2}= 16.97, F_{mNrip1}= 0.06133
9D	P=0.0021	F= 12.72
9E	P<0.0001, P<0.0001, P<0.0001	$F_{treatment}$ = 186.0, F_{time}= 155.7, $F_{treatment \times time}$= 144.1
9F	P<0.0001, P<0.0001, P<0.0001	$F_{treatment}$ = 183.3, F_{time}= 198.0, $F_{treatment \times time}$= 183.3
9G	P<0.0001, P<0.0001, P<0.0001	$F_{treatment}$ = 387.4, F_{time}= 300.9, $F_{treatment \times time}$= 303.8
9H	P<0.0001, P<0.0001, P<0.0001	$F_{treatment}$ = 380.5, F_{time}= 395.4, $F_{treatment \times time}$= 503.9
9I	P<0.0001, P<0.0001	F= 64.63
9J	p_{GFAP} <0.0001, p_{p-ERK1} <0.0001, p_{p-ERK2} <0.0001, p_{ERK1}= 0.0340, p_{ERK2}= 0.4664	F_{GFAP}= 34.25, F_{p-ERK1}=105.1, F_{pERK2}= 103.9, F_{ERK1}= 4.787, F_{ERK2}= 0.9375
9K	P=0.0096, P=0.0098	F= 8.678
9L	P<0.0001, P<0.0001	F= 303.8
10B	P=0.004	t= 5.967
10C	P=0.0046, P=0.0315	F= 14.3
10D	P<0.0001	$F_{treatment}$ = 61.32, F_{time}= 1900, $F_{treatment \times time}$= 16.07
	P<0.0001	F= 84.56
10E	P<0.0001	F= 663.6
10H	P=0.0003, P<0.0001	F_{tlr2} = 21.47, $F_{Syncrip}$= 80.23
10I	P=0.0091, P=0.0031	F_{tlr2} = 7.333, $F_{Syncrip}$= 11.20
10J	P=0.0068, P<0.0001	F_{tlr2} = 8.678, $F_{Syncrip}$= 303.8

Supplementary Figurs

Fig	*p* value	*F* value
3A	P=0.8055, P=0.2514, P=0.6254	$F_{treatment}$ = 0.2446, F_{time}= 1.424, $F_{treatment \times time}$= 2.219
3B	P=0.8216, P=0.6946, P=0.4953	$F_{treatment}$ = 0.05195, F_{time}= 0.6079, $F_{treatment \times time}$= 0.9030
3C	P=0.9009, P=0.7483, P=0.1340	$F_{treatment}$ = 0.01585, F_{time}= 0.5342, $F_{treatment \times time}$= 1.888
3D	P=0.0577	F = 3.864
3E	P=0.6649	F= 0.6099
3F	P=0.2019	F= 1.067
5	P <0.0001	F_{Fus}= 83.22
7A	P_{cNrip1}=0.0015, P_{mNrip1}=0.6369	F_{time}= 879.4, $F_{treatment}$= 47.03, $F_{treatment \times time}$= 52.25
7B	P<0.0001, P<0.0001, P<0.0001	F_{time}= 1287, $F_{treatment}$= 126.1, $F_{treatment \times time}$= 145.2
7C	P<0.0001, P<0.0001, P<0.0001	F_{time}= 3644, $F_{treatment}$= 167.8, $F_{treatment \times time}$= 164.5
7D	P<0.0001, P<0.0001, P<0.0001	F_{time}= 2684, $F_{treatment}$= 222.8, $F_{treatment \times time}$= 189.5
7E	P =0.6458, P =0.6458, P=0.4907	F_{time}= 0.2118, $F_{treatment}$= 0.2118, $F_{treatment \times time}$= 0.4765
7F	P=0.3174, P= 0.8025, P=0.8025	F_{time}= 1.003, $F_{treatment}$=0.06272, $F_{treatment \times time}$ =0.06272
7G	P=0.2048, P=0. 3759, P=0.5233	F_{time}= 1.616, $F_{treatment}$= 0.7869, $F_{treatment \times time}$= 0.3913
7H	P<0.0001	F= 30.99
7I	P<0.0001	F= 36.31
7J	p_{GFAP} <0.0001, p_{p-ERK1} = 0.0006, p_{p-ERK2} <0.0001, p_{ERK1}= 0.4627, p_{ERK2}= 0.8348	F_{GFAP}= 114.9, F_{p-ERK1}=18.52, F_{pERK2}= 65.92, F_{ERK1}= 0.9464, F_{ERK2}= 0.2853
8A	P =0.8888, P =0.4630, P=0.8888	F_{time}= 0.01961, $F_{treatment}$= 0.1765, $F_{treatment \times time}$= 0.01961
8B	P=0.5732, P= 0.2605, P=0.1600	F_{time}= 0.3184, $F_{treatment}$=1.274, $F_{treatment \times time}$ =1.990
8C	P=0.3033, P=0. 4694, P=0.8274	F_{time}= 1.066, $F_{treatment}$= 0.5255, $F_{treatment \times time}$= 0. 04766
8D	P<0.0001	F= 14.91
8E	P<0.0001	F= 15.47

Fig	*p* value	*F* value
8F	p_{GFAP} <0.0001, p_{p-ERK1} <0.0001, p_{p-ERK2} <0.0001, p_{ERK1}= 0.9431, p_{ERK2}= 0.8364	F_{GFAP}= 54.03, F_{p-ERK1}=45.63, F_{pERK2}= 73.34, F_{ERK1}= 0.1243, F_{ERK2}= 0.2829
10A	p_{GFAP} =0.0066, p_{p-ERK1} <0.0001, p_{p-ERK2} <0.0001, p_{ERK1}= 0.6811, p_{ERK2}= 0.5366	F_{GFAP}= 8.770, F_{p-ERK1}=94.73, F_{pERK2}= 146.5, F_{ERK1}= 0.5186, F_{ERK2}= 0.7818
10B	P >0.9999, P =0. 6468, P=0.8187	F_{time}= 0.0000, $F_{treatment}$= 0.2105, $F_{treatment \times time}$= 0.05263
10C	P=0.4881, P= 0.2483, P=0.8171	F_{time}= 0.4821, $F_{treatment}$=1.339, $F_{treatment \times time}$ =0.05357
10D	P=0.8574, P=0. 5813, P=0.7555	F_{time}= 0.03234, $F_{treatment}$= 0.3050, $F_{treatment \times time}$= 0. 09721
11A	P >0.9999, P= 0.8187, P=0.6486	F_{time}= 0.0000, $F_{treatment}$=0.1535, $F_{treatment \times time}$ =0.2105
11B	P=0.8574, P=0. 5813, P=0.7555	F_{time}= 0.8597, $F_{treatment}$= 0.3050, $F_{treatment \times time}$= 0. 09721
11C	P=0.4881, P= 0.2483, P=0.8171	F_{time}= 0.4821, $F_{treatment}$=1.339, $F_{treatment \times time}$ =0.05357
11D	P >0.9999, P=0. 8179, P=0.6453	F_{time}= 0.000, $F_{treatment}$= 0.05310, $F_{treatment \times time}$= 0. 2124
11E	P=0.1636, P= 0. 3525, P=0.8161	F_{time}= 1.952, $F_{treatment}$=0.8675, $F_{treatment \times time}$ =0.05422
11F	P=0.5822, P=0. 5492, P=0.7005	F_{time}= 0.3035, $F_{treatment}$= 0.8279, $F_{treatment \times time}$= 0. 1483
13A	P<0.0001	F= 18.75
13B	p_{GFAP} =0.0003, p_{p-ERK1} <0.0001, p_{p-ERK2} <0.0001, p_{ERK1}= 0.7498, p_{ERK2}= 0.3294	F_{GFAP}= 21.98, F_{p-ERK1}=45.99, F_{pERK2}= 96.43, F_{ERK1}= 0.4107, F_{ERK2}= 0.8045
13C	P =0.9351, P=0. 8179, P=0.6453	F_{time}= 0.9351, $F_{treatment}$=0.1039, $F_{treatment \times time}$ =0.1039
13D	P=0.3350, P= 0.7476, P=0.7476	F_{time}= 0.3035, $F_{treatment}$= 0.8279, $F_{treatment \times time}$= 0. 1483
13E	P=0.7865, P= 0. 7605, P=0.9480	F_{time}= 0.07632, $F_{treatment}$= 0.09317, $F_{treatment \times time}$= 0. 1810
14A	P >0.9999, P= 0.9813, P=0.9991	F_{time}= 0.0000, $F_{treatment}$=0.1821, $F_{treatment \times time}$ =0.06069
14B	P=0.5022, P=0. 5416, P=0.3216	F_{time}= 0.4528, $F_{treatment}$= 0.8396, $F_{treatment \times time}$= 1.179
14C	P=0.7459, P= 0.3533, P=0.9093	F_{time}= 0.1055, $F_{treatment}$=1.119, $F_{treatment \times time}$ =0.3419
15A	P<0.0001	F= 15.82
15B	P =0.9716, P= 0.6855, P=0.9766	F_{time}= 0.4214, $F_{treatment}$= 0.6190, $F_{treatment \times time}$= 0. 06858
15C	P=0.7205, P=0. 8513, P=0.5682	F_{time}= 0.1284, $F_{treatment}$= 0.03522, $F_{treatment \times time}$= 0. 3985
15D	P=0.8809, P= 0.6021, P=0.7493	F_{time}= 0.02252, $F_{treatment}$=0.2728, $F_{treatment \times time}$ =0.1024
17A	P<0.0001, P<0.0001, P<0.0001	F_{time}= 385.4, $F_{treatment}$= 29.54, $F_{treatment \times time}$= 27.18
17B	P<0.0001, P<0.0001, P<0.0001	F_{time}= 772.7, $F_{treatment}$= 128.0, $F_{treatment \times time}$= 167.2
17C	P<0.0001, P<0.0001, P<0.0001	F_{time}= 1746, $F_{treatment}$= 64.08, $F_{treatment \times time}$= 67.17
17D	P =0.6458, P =0.6458, P=0.4907	F_{time}= 2671, $F_{treatment}$= 177.3, $F_{treatment \times time}$= 147.6
17E	P >0.9999, P= 0.7885, P >0.9999	F_{time}= 0.000, $F_{treatment}$= 0.07612, $F_{treatment \times time}$= 0.000
17F	P=0.8272, P=0. 5127, P=0.1276	F_{time}= 0.04778, $F_{treatment}$=0.4300, $F_{treatment \times time}$ =2.341
17G	P=0.7127, P=0. 5771, P=0.9537	F_{time}= 0.1360, $F_{treatment}$= 0.3119, $F_{treatment \times time}$= 0.2623
18A	P<0.0001, P=0.0013, P=0.0013	F_{time}=135.0, $F_{treatment}$= 11.27, $F_{treatment \times time}$= 11.27
18B	P<0.0001, P<0.0001, P<0.0001	F_{time}= 251.5, $F_{treatment}$= 18.23, $F_{treatment \times time}$= 18.23
18C	P<0.0001, P<0.0001, P<0.0001	F_{time}= 236.9, $F_{treatment}$= 40.60, $F_{treatment \times time}$= 30.22
18D	P<0.0001, P<0.0001, P<0.0001	F_{time}= 1129, $F_{treatment}$= 102.5, $F_{treatment \times time}$= 153.6
